# A Case for estradiol: younger brains in women with earlier menarche and later menopause

**DOI:** 10.1093/gigascience/giaf060

**Published:** 2025-05-23

**Authors:** Eileen Luders, Inger Sundström Poromaa, Claudia Barth, Christian Gaser

**Affiliations:** Department of Women’s and Children’s Health, Uppsala University, Uppsala 75185, Sweden; Swedish Collegium for Advanced Study (SCAS), Uppsala 75236, Sweden; School of Psychology, University of Auckland, Auckland 1010, New Zealand; Laboratory of Neuro Imaging, School of Medicine, University of Southern California, Los Angeles, CA 90033, USA; Department of Women’s and Children’s Health, Uppsala University, Uppsala 75185, Sweden; Department of Psychiatric Research, Diakonhjemmet Hospital, Oslo 0370, Norway; Department of Psychiatry and Psychotherapy, Jena University Hospital, Jena 07743, Germany; Department of Neurology, Jena University Hospital, Jena 07743, Germany; German Center for Mental Health (DZPG), Germany

**Keywords:** brain age, estradiol, machine learning, menarche, MRI, menopause, structural neuroimaging

## Abstract

The transition to menopause is marked by a gradual decrease of estradiol. Concurrently, the risk of dementia in women increases around menopause, suggesting that estradiol (or the lack thereof) plays a role in the development of dementia and other age-related neuropathologies. Here, we set out to investigate whether there is a link between brain aging and estradiol-associated events, such as menarche and menopause. For this purpose, we applied a well-validated machine learning approach to analyze both cross-sectional and longitudinal data from a sample of 1,006 postmenopausal women who underwent structural magnetic resonance imaging twice, approximately 2 years apart. We observed less brain aging in women with an earlier menarche, a later menopause, and a longer reproductive span (i.e., the time interval between menarche and menopause). These effects were evident both cross-sectionally and longitudinally, supporting the notion that estradiol has neuroprotective properties and contributes to brain preservation. However, further research is required because the observed effects were small, estradiol was not directly measured, and other factors may modulate female brain health. Future studies might benefit from incorporating actual estradiol (and other hormone) measures, as well as considering genetic predispositions and lifestyle factors alongside indicators of brain aging to deepen our understanding of estradiol’s role in maintaining brain health. Additionally, including more diverse study populations (e.g., varying in ethnicity, socioeconomic status, and health status) in follow-up research would enhance the generalizability and applicability of these findings.

## Background

Estradiol is the most potent and prevalent form of estrogen during the reproductive life of a woman [1]. Generally speaking, estradiol levels start increasing just before the first menstrual period (menarche) and then plateau on a high level until they start decreasing during perimenopause. After the final menstrual period (i.e., menopause), estradiol levels decrease further and eventually reach plateauing low levels during postmenopause [2]. The risk for dementia in women is known to increase around menopause [3–6], and thus it stands to reason that estradiol plays a role for the development of dementia and other age-related neuropathologies. Indeed, studies using animal models have demonstrated that estradiol promotes synaptic plasticity, enhances neurogenesis, and protects against oxidative stress and neuroinflammation [7–13]—mechanisms that are critical for maintaining brain health and mitigating age-related brain degeneration. While extensive research has also been conducted in humans, focusing on specific phases (e.g., menarche, pregnancy, menopause) or interventions (e.g., hormonal contraceptives, menopausal estrogen therapy and antiestrogen therapy), definitive evidence for the neuroprotective role of estradiol remains elusive [4, 14–29]. Specifically, in the context of menarche and menopause, both early and late onset have been associated with an increased risk of dementia as well as with markers of brain aging and cognitive function [4, 14–21, 24–26].

To further advance this field of research, the current study set out to determine if there is a link between a woman’s estimated brain age (a biological marker of brain health [30]) and the reproductive span (i.e., the interval between menarche and menopause when estradiol levels are high). If a lack of estradiol is among the driving factors for diminished brain health later in life, brain age and reproductive span should be inversely related (negative correlation). To be able to relate our findings to others in the literature [14–16] and to provide a frame of reference for future studies, we additionally investigated if there is a significant link between estimated brain age and the age at menarche as well as the age at menopause. Assuming a neuroprotective effect of estradiol, we expected that a lower brain age would be linked to an earlier menarche (positive correlation) and to a later menopause (negative correlation). Importantly, our study comprises both cross-sectional and longitudinal components, with follow-up data acquired approximately 2 years after the initial brain scan.

To estimate brain age, we used structural brain images and a well-validated high-dimensional pattern recognition approach, as detailed elsewhere. Briefly, the difference between the estimated brain age and the chronological age yields a so-called brain age gap estimate (BrainAGE) in years. The BrainAGE index is negative if a brain is estimated younger than its chronological age; it is positive if a brain is estimated older than its chronological age. For example, a 50-year-old woman with a BrainAGE index of −3 years shows the aging pattern of a 47-year-old. The BrainAGE algorithm has been shown to be robust and reliable across datasets, age ranges, and scanner types [31, 33]; it has been successfully applied in a wide range of studies [31, 32, 34–36], including those capturing hormonal changes in women [37, 38]. Moreover, the BrainAGE index has been demonstrated to work as a predictor of dementia as well as age-related cognitive decline [34, 39]. A major advantage of the BrainAGE approach is its ability to aggregate complex, spatially distributed age-related changes in brain structure into a single, interpretable biomarker. Such brain age metrics provide a powerful way to study the influence of biological factors across the female lifespan, including the effects of cumulative estrogen exposure and genetic risk for age-related brain degeneration [14].

## Materials and Methods

### Data description

**Figure 1:**
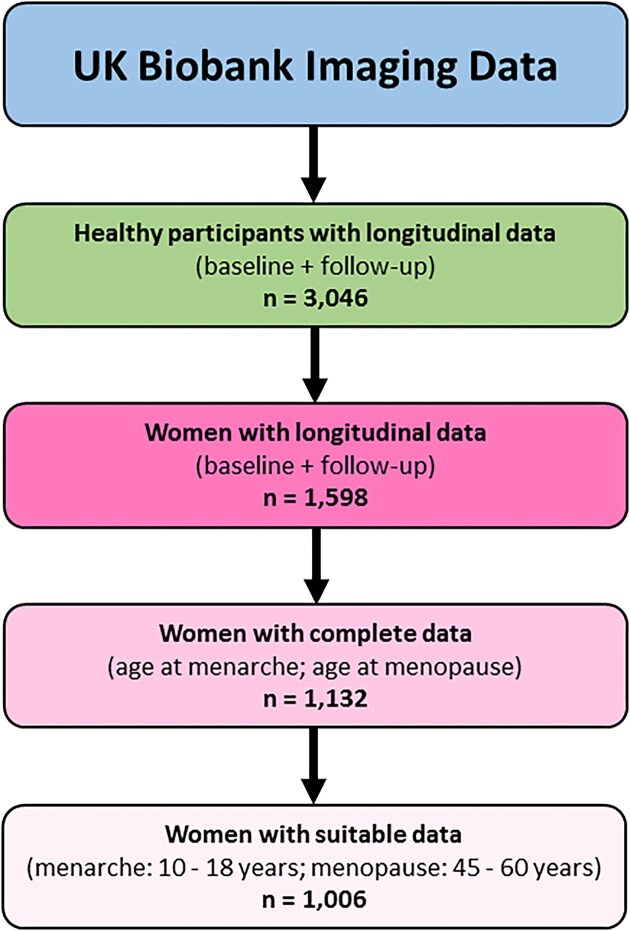
Flowchart of sample selection.

The study is based on a carefully selected sample of 1,006 postmenopausal women from the UK Biobank [40], which was accessed under application number #41,655. The UK Biobank is a biomedical database and research resource that contains genetic, lifestyle, and health information from half a million people. In the UK Biobank cohort, 94.6% of participants are of white ethnicity [41]. For general ethnic information, see [42]; for ethnic information on all women with available longitudinal data, see Supplemental Table S3. The UK Biobank holds the ethical approval from the North West Multi-Centre Research Ethics Committee and is in possession of the informed consents. Written informed consent was obtained from all participants. Inclusion criteria for the current study were women with available longitudinal data as well as information on age at menarche and age at menopause. Exclusion criteria for the current study were preexisting neurological or psychiatric diagnoses as per UK Biobank data fields #41,202–0.0 to #41,202–0.78. In addition, to further increase the homogeneity of the sample, we excluded women whose age at menarche was younger than 10 or older than 18, or whose age at menopause was younger than 45 or older than 60. This resulted in a final sample size of 1,006 women. Table 1 provides information on this final sample; Fig. 1 summarizes the steps related to the sample selection. For each woman, 1 initial brain scan and 1 follow-up brain scan—approximately 2 years apart (mean ± SD: 2.35 ± 6.12 years)—were obtained *after* menopause. These T1-weighted brain images were acquired on a 3 Tesla Siemens Skyra scanner using a 32-channel head coil, as described elsewhere [43, 44].

**Table 1:** Sample characteristics

Variable	Descriptive Statistics
Age at the initial brain scan	Mean ± SD: 63.20 ± 6.42 years
Age at the follow-up brain scan	Mean ± SD: 65.54 ± 6.37 years
Age at menarche	Mean ± SD: 13.02 ± 1.53 years
Age at menopause	Mean ± SD: 51.41 ± 3.23 years
Reproductive span	Mean ± SD: 38.39 ± 3.55 years
Number of live births	Mean ± SD: 1.75 ± 1.16
Number of women with hormone replacement therapy	Yes: 306 (30.42%) | no: 700 (69.58%)
Number of women with hysterectomy	Yes: 48 (4.77%) | no: 958 (95.23%)
Number of women with bilateral oophorectomy	Yes: 37 (3.68%)| no: 969 (96.32%)

SD: standard deviation.

### Data analyses

Using the aforementioned T1-weighted images, we applied a number of processing routines implemented in the CAT12 toolbox [45] (version 12.8), which resulted in bias-corrected, spatially normalized, and tissue-classified brain images, as detailed elsewhere [31, 38]. The normalized gray and white matter partitions were smoothed using a 4- and 8-mm full-width-at-half-maximum Gaussian kernel, and image resolution was set to 4 and 8 mm. For further data reduction, we applied a principal component analysis (PCA) using singular value decomposition to all the models using *n* – 1 PCA components (*n* = minimum of voxel number or sample size). Prior to applying the PCA, the data were normalized by scaling the values between 0 and 1 and subtracting the mean. The transformation matrix derived from the training data PCA was used to project the normalized test data onto this principal component space.

For the estimation of the BrainAGE index, we employed a Gaussian process regression [46] that uses a linear covariance function, a constant mean function, and a Gaussian likelihood function. Hyperparameters were set to 100 for the constant mean function and to −1 for the likelihood function based on prior exploratory analyses [33]. As training data, we selected 3,046 individuals from the UK Biobank where 2 time points were available. To avoid overfitting and ensure generalizability, we employed 10-fold cross-validation separately for the initial and follow-up brain scan, where the dataset was randomly partitioned into 10 equally sized subsets. In each iteration, 1-fold was used as the test set and the remaining 9 as the training set. This process was repeated 10 times, and performance metrics (e.g., mean absolute error) were averaged across folds. To estimate the individual brain ages, 8 models based on the aforementioned sets of images (i.e., gray matter/white matter, 4-mm/8-mm Gaussian kernel, and 4-mm/8-mm image resolution) were combined using a general linear model where the weights of the models were derived by maximizing the variance to the parameter of interest (e.g., menopause). The difference between the resulting estimated brain age and the chronological age was then calculated as the BrainAGE index (in years).

### Statistical analyses

#### Main analyses

After computing the BrainAGE index for all 1,006 women at initial and follow-up scan, we first removed the linear age trend that is typically seen in BrainAGE estimation. Then, we conducted 2 analysis streams using linear regressions in MATLAB (version R2023b; RRID:SCR_001622), one cross-sectional and one longitudinal. For all analyses, alpha was set at 0.05 (2-tailed). For the cross-sectional stream, we tested if there is a significant link between the BrainAGE index at the initial brain scan and the reproductive span. In addition, we tested if there is a significant link between the BrainAGE index at the initial brain scan and the age at menarche as well as the age at menopause. For the longitudinal stream, we first subtracted the BrainAGE index at the initial brain scan from the BrainAGE index at the follow-up brain scan, which resulted in a ∆ BrainAGE index for each woman. This method, often referred to as “change score” analysis, produces statistical results comparable to those resulting from a repeated-measures analysis of variance with 2 time points. Using the ∆ BrainAGE index, we then tested for significant links with the reproductive span, the age at menarche, and the age at menopause.

#### Sensitivity analyses

The aforementioned main analyses were repeated while accounting for potential confounds known to affect brain health. More specifically, we removed the variance associated with the number of live births [47] (UK Biobank data field #2734), hormone replacement therapy [14] (#2814), hysterectomy [48] (#3591), bilateral oophorectomy [48] (#834), body mass index [49] (#21,001), diastolic and systolic blood pressure [50] (#4079 and #4080), diabetes [51] (#2443), education [52] (#6138), income [53] (#738), and a composite lifestyle factor [54]. The latter was expressed as a general lifestyle score that was calculated based on a number of factors (see Supplemental Table S1), known to increase/decrease the risk of adverse cardiovascular events. Since not all women had information on all potential confounds (see Supplemental Table S2), we applied an imputation method using the MATLAB function “fillmissing.” That is, missing entries were replaced with the corresponding values from the nearest neighbor rows, calculated based on the pairwise Euclidean distance between rows. Imputation was applied to up to 295 women, depending on the potential confound. For the cross-sectional stream, we tested if there is a significant link between the BrainAGE index at the initial brain scan and the reproductive span (age at menarche and age at menopause, respectively). Likewise, for the longitudinal stream, we tested if there is a significant link between the ∆ BrainAGE index and the reproductive span (age at menarche and age at menopause, respectively).

## Results

### Main analyses

**Figure 2:**
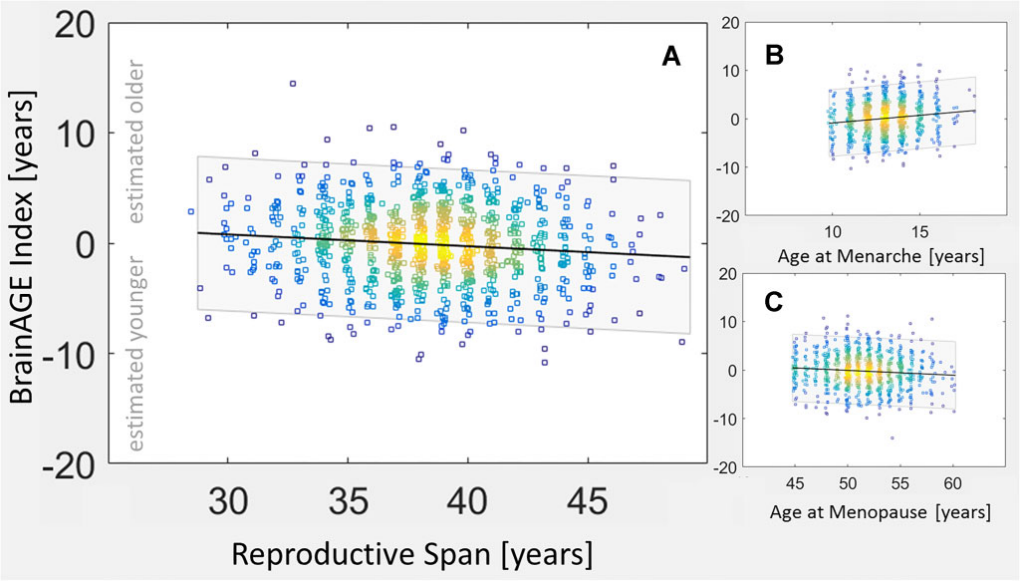
Correlations with BrainAGE at the initial brain scan. The x-axes show the reproductive span (age, respectively) in years. Of note, age in the UK Biobank has been rounded to the year, so we added a small random jitter to the x-axes to give a better overview about the age distribution. The y-axes show the BrainAGE index in years, with negative values indicating that brains are estimated younger than their chronological age and positive values indicating that brains are estimated older than their chronological age. Panel A displays a negative link between the BrainAGE index and the reproductive span (the longer the reproductive span, the younger the estimated brain age). Panel B displays a positive link between the BrainAGE index and the age at menarche (the earlier the onset of menarche, the younger the estimated brain age). Panel C displays a negative link between the BrainAGE index and the age at menopause (the later the onset of menopause, the younger the estimated brain age). The squares in the density plot represent the individual measures (*n* = 1,006); hot colors indicate a larger overlay of measures; cool colors indicate a smaller overlay. The shaded band is the 95% confidence interval.

As shown in Fig. 2 (left), our cross-sectional analyses revealed a significant negative association between BrainAGE and the reproductive span. In other words, brains of women with longer reproductive spans were estimated younger than brains of women with shorter reproductive spans. As also shown in Fig. 2 (right), there was a significant positive association between BrainAGE and age at menarche (i.e., the earlier the menarche, the younger the brain) and a significant negative association between BrainAGE and age at menopause (i.e., the later the menopause, the younger the brain). As shown in Table 2 (main analyses), effect sizes were small [55], with *r*-values of −0.11, 0.14, and −0.09 for reproductive span, menarche, and menopause, respectively. The slopes of the regression indicate different rates of change for menarche and menopause (0.32 and −0.10, respectively). More specifically, for each year younger at menarche, brains are estimated 0.32 years younger (which corresponds to 3.2 years younger for each 10 years). In contrast, for each year older at menopause, brains are estimated 0.1 year younger (which corresponds to 1 year younger for each 10 years).

**Table 2:** Associations with BrainAGE at the initial brain scan

	Main analyses					Sensitivity analyses*				
	*R* ^2^	*r*	*P*	Slope	95% CI	*R* ^2^	*r*	*P*	Slope	95% CI
Reproductive span	0.01	−0.11	<0.001	−0.11	−0.17 to −0.05	0.01	−0.11	<0.001	−0.11	−0.17 to −0.05
Age at menarche	0.02	0.14	<0.001	0.32	0.18 to 0.46	0.02	0.14	<0.001	0.33	0.19 to 0.47
Age at menopause	0.01	−0.09	<0.005	−0.10	−0.17 to −0.03	0.01	−0.09	<0.01	−0.09	−0.16 to −0.03

* While removing the variance associated with the number of live births, hormone replacement therapy, hysterectomy, bilateral oophorectomy, body mass index, diastolic and systolic blood pressure, diabetes, education, income, and a composite lifestyle factor.

**Figure 3:**
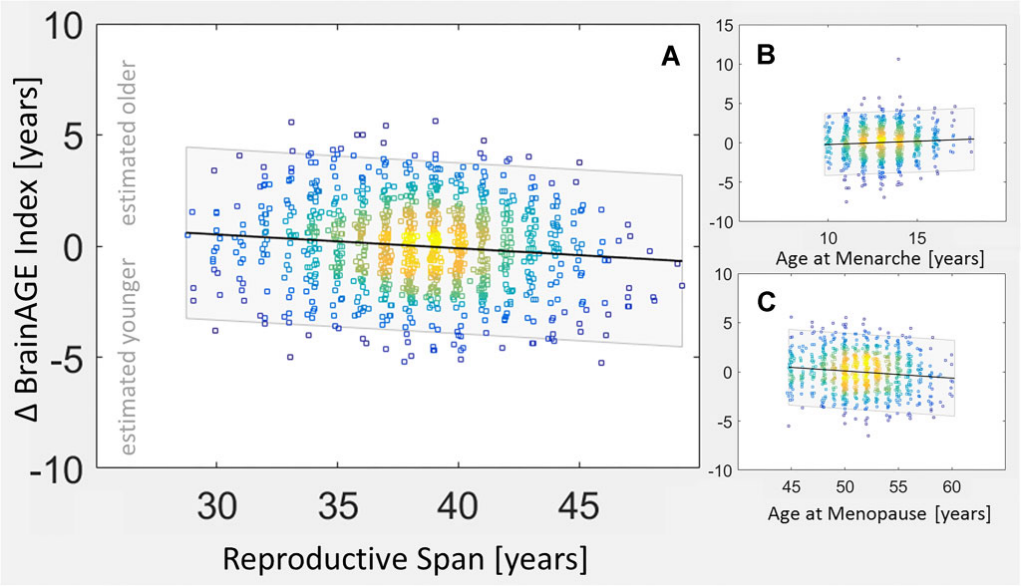
Correlations with BrainAGE over 2.35 years (∆ BrainAGE). Panel A displays a negative link between the BrainAGE index and reproductive span (the longer the reproductive span, the younger the estimated brain age). Panel B displays a positive link between the BrainAGE index and the age at menarche (the earlier the onset of menarche, the younger the estimated brain age). Panel C displays a negative link between the BrainAGE index and the age at menopause (the later the onset of menopause, the younger the estimated brain age). The squares in the density plot represent the individual measures (*n* = 1,006); hot colors indicate a larger overlay of measures; cool colors indicate a smaller overlay. The shaded band is the 95% confidence interval.

As shown in Fig. 3 and Table 3 (main analyses), our longitudinal findings confirm the observed cross-sectional relationships. More specifically, ∆ BrainAGE was negatively linked to reproductive span and menopause, and positively linked to age at menarche. All associations were significant. Again, effect sizes were small, with *r*-values of −0.12, 0.06, and −0.12 for reproductive span, menarche, and menopause, respectively. The slopes of the regression are still somewhat different for menarche and menopause (0.08 and −0.06, respectively), albeit more similar than in the cross-sectional analysis: for each year younger at menarche, brains are estimated 0.08 years younger (0.8 years over 10 years), whereas for each year older at menopause, brains are estimated 0.06 years younger (0.6 years over 10 years).

**Table 3:** Associations with changes in BrainAGE over 2.35 years

	Main analyses					Sensitivity analyses*				
	*R* ^2^	*r*	*P*	Slope	95% CI	*R* ^2^	*r*	*P*	Slope	95% CI
Reproductive span	0.01	−0.12	<0.001	−0.06	−0.10 to −0.03	0.01	−0.11	<0.001	−0.06	−0.09 to −0.03
Age at menarche	<0.01	0.06	<0.05	0.08	0.0 to 0.16	<0.01	0.06	n.s.	0.08	0.0 to 0.16
Age at menopause	0.01	−0.12	<0.001	−0.07	−0.10 to −0.03	0.01	−0.12	<0.001	−0.07	−0.11 to −0.03

* While removing the variance associated with the number of live births, hormone replacement therapy, hysterectomy, bilateral oophorectomy, body mass index, diastolic and systolic blood pressure, diabetes, education, income, and a composite lifestyle factor.

n.s.: not significant.

### Sensitivity analyses

The results described above remained comparable when removing the variance associated with the number of live births, hormone replacement therapy, hysterectomy, bilateral oophorectomy, body mass index, diastolic and systolic blood pressure, diabetes, education, income, and a composite lifestyle factor. In other words, when examining the association between BrainAGE and reproductive span, we observed a negative association. Likewise, there was a positive association between BrainAGE and age at menarche and a negative association between BrainAGE and age at menopause. The effects were significant for reproductive span, menarche, and menopause for the cross-sectional analyses (see Table 2, Sensitivity analyses) and for reproductive span and menopause for the longitudinal analyses (see Table 3, Sensitivity analyses).

## Discussion

Here we assessed links between estimated brain age and milestones in a woman’s reproductive life in a well-powered sample of more than a thousand postmenopausal women. We detected less brain aging in women with longer reproductive spans, earlier menarche, and later menopause (see Figs. 2 and 3 and Tables 2 and 3).

### Correspondence with previous findings

Our findings are in line with the outcomes of other studies suggesting a longer reproductive span, an earlier menarche [20, 21], and a later menopause to be associated with a lower risk of developing dementia or better retained cognitive function. Furthermore, given that the BrainAGE index is based on the weighted distribution of gray and whiter matter tissue in the brain, our findings are also in agreement with reports of lower brain volumes as well as higher rates of brain tissue loss during menopause compared to premenopause or in postmenopausal women compared to premenopausal women [56–58]. In addition, our findings agree with observed effects across the menstrual cycle linking high estradiol levels at ovulation to lower BrainAGE estimates [37]. Altogether, the outcomes of our study seem to suggest that estradiol contributes to brain health, which is in agreement with other studies reporting positive effects of estradiol on brain health and cognition within the framework of aging and/or menopausal hormone therapy [59–63].

### Menarche versus menopause

The outcomes of the main analyses indicate that both an earlier menarche and a later menopause are significantly associated with less brain aging. However, menarche and menopause differ with respect to the strength of their relationship with age (which is reflected in the correlation coefficient) and their rate of change with age (which is reflected in the slope of the regression line). This might indicate somewhat different underlying biological mechanisms and/or confounds for menarche and menopause. For example, during menopause, in addition to decreasing levels of estradiol, increasing levels of follicle-stimulating hormones may cause an accelerated deposition of amyloid-β and Tau [64], which enhances brain atrophy. Moreover, menopause is marked by disadvantageous alterations in cytokine and T-cell profiles [65], which are linked to an enhanced inflammation. Alternatively, the less strong link pertaining to menarche could also reflect the fact that, later in life, it is probably more challenging to accurately remember the onset of menarche than the onset of menopause.

### Potential implications

Given that estradiol levels start decreasing during perimenopause and further decrease after menopause, our findings may explain why the risk for dementia in women is known to increase around menopause [3–6] and why there is an increased age-independent prevalence of Alzheimer’s disease in women compared to men [63]. Moreover, our findings seem to support the concept of the “window of opportunity,” spanning the years leading up to menopause to the years immediately after menopause, where health interventions (e.g., menopausal hormone treatment) may combat the increased risk for Alzheimer’s disease in some women [5, 66–68]. In fact, several large-scale projects have investigated the effects of menopausal hormone treatment on cognitive function and Alzheimer’s risk, but results are inconclusive (potentially relevant modulators of treatment outcomes are discussed elsewhere [59, 69–74]).

The current findings seem to suggest a protective effect of estradiol and, as such, seem promising in the framework of prevention and intervention. However, further research is required, as the effect sizes for the observed associations were small (albeit smaller effect sizes are not uncommon in studies with larger sample sizes [75]), and various factors, such as genetics, lifestyle, or hormones other than estradiol, could play a greater (or at least an additional) role in preserving brain health [2, 76, 77]. Moreover, our study did not measure estradiol directly, and links between estradiol and brain aging seem to be rather complex, as indicated by the outcomes of other studies. For example, it was reported that, compared to no exposure or no dose, exposure to low concentrations of estradiol or low doses of estrogen enhanced neuronal survival and increased anti-inflammatory markers (i.e., positive links), while exposure to high concentrations of estradiol as well as high doses of estrogen had the opposite effect (i.e., negative links) [27, 28]. Another study reported U-shaped curves suggesting that both early and late menarche are associated with an increased risk for dementia (i.e., positive and negative links) [15], and yet another study reported either negative links or missing links between age at menarche and brain aging depending on the potential confounds accounted for [14]. Interestingly, this latter study also reported that, in carriers of the apolipoprotein E type 4 allele (APOE e4), higher levels of estradiol at menopause were associated with increased brain aging (positive link). In contrast, in noncarriers, higher levels of estradiol at menopause were associated with decreased brain aging (negative link) [14].

## Conclusion

Our study revealed less brain aging in women with a larger reproductive span, earlier menarche, and later menopause. Thus, sex hormones—potentially estradiol—may contribute to brain health. However, follow-up research is required because the effects observed in the current study were small, estradiol was not directly examined, and female brain health is likely also modulated by factors other than estradiol. Future studies might benefit from incorporating actual estradiol (and other hormone) measurements, as well as considering genetic predispositions and lifestyle factors alongside structural brain measures. Moreover, to build a more comprehensive understanding and expand this understudied field, future research focusing on specific time frames surrounding menopause—such as perimenopause (i.e., the time preceding the final menstrual period) or early postmenopause (e.g., the initial year after menopause) versus late menopause (e.g., 10 years after menopause)—would be valuable. Lastly, the UK Biobank (i.e., the source of the current sample) is biased toward healthy and more socioeconomically privileged individuals with a predominantly white ethnic background [41]. Thus, conducting research in more diverse populations, including individuals from different ethnic, socioeconomic, and health backgrounds, would improve the generalizability of findings and provide a broader understanding of the relationship between estradiol and brain health.

## Availability of Source Code and Requirements

Project name: BrainAGE-UKBiobank

Project homepage: https://github.com/ChristianGaser/BrainAGE-UKBiobank

Operating system(s): Platform independent

Programming language: MATLAB

Other requirements: SPM12, CAT12, BrainAGE

License: GNU GPL-3.0

## Supplementary Material

giaf060_Supplemental_File

giaf060_Authors_Response_To_Reviewer_Comments_original_submission

giaf060_Authors_Response_To_Reviewer_Comments_Revision_1

giaf060_GIGA-D-24-00418_original_submission

giaf060_GIGA-D-24-00418_Revision_1

giaf060_GIGA-D-24-00418_Revision_2

giaf060_Reviewer_1_Report_original_submission Leonardo Bertolin Furstenau -- 12/15/2024

giaf060_Reviewer_1_Report_Revision_1 Leonardo Bertolin Furstenau -- 4/9/2025

giaf060_Reviewer_2_Report_original_submission Michael Lombardo -- 1/9/2025

## Data Availability

The individual-level proxy measures obtained from the prediction models in this work will be shared after publication in agreement with UK Biobank regulations. Snapshots of our GitHub are in Software Heritage [78], and the DOME-ML annotations for this work can be found in the DOME Registry [79]. The input data are available to other researchers through the UK Biobank’s controlled access scheme. The procedure to apply for access [80] requires registering with the UK Biobank and completion of an application form detailing: A summary of the planned research The UK Biobank data fields required for the project A description of derivatives (data, variables) generated by the project

## Additional Files

**Supplemental Table S1**. A general lifestyle score was calculated based on 16 variables.

**Supplemental Table S2**. Number of women with missing information (imputed values).

**Supplemental Table S3**. Ethnic background of women with longitudinal MRI data (*n* = 1,598).

## Abbreviations

BrainAGE: brain age gap estimate; MRI: magnetic resonance imaging; PCA: principal component analysis.
