# Supplementary material for: A Case for estradiol: younger brains in women with earlier menarche and later menopause
Source: Gigascience. 2025 May 23;14:giaf060. doi: 10.1093/gigascience/giaf060 (PMC12099614; doi:10.1093/gigascience/giaf060)
Supplement: giaf060_Supplemental_File [file giaf060_supplemental_file.docx]

**Supplemental Tables**

**Supplemental Table 1.** A general lifestyle score was calculated based on 16 variables.

| **Variables** | **UK Biobank Data Field Number** |
| --- | --- |
| 1. Time spend watching TV | #1070 |
| 1. Sleep duration | #1160 |
| 1. Current tobacco smoking | #1239 |
| 1. Past tobacco smoking | #1249 |
| 1. Cooked vegetable intake | #1289 |
| 1. Salad / raw vegetable intake | #1299 |
| 1. Fresh fruit intake | #1309 |
| 1. Dried fruit intake | #1319 |
| 1. Oily fish intake | #1329 |
| 1. Processed meat intake | #1349 |
| 1. Beef intake | #1369 |
| 1. Lamb mutton intake | #1379 |
| 1. Pork intake | #1389 |
| 1. Alcohol intake frequency | #1558 |
| 1. Moderate activity | #884 |
| 1. Vigorous activity | #904 |

**Supplemental Table 2.** Number of women with missing information (imputed values)

| number of live births | 8 |
| --- | --- |
| hormone replacement therapy | 12 |
| hysterectomy | 146 |
| bilateral oophorectomy | 16 |
| body mass index | 95 |
| diastolic blood pressure | 474 |
| systolic blood pressure | 474 |
| diabetes | 9 |
| education | 90 |
| income | 174 |
| composite lifestyle factor | 147 |

**Supplemental Table 3.** Ethnic background of women with longitudinal MRI data (n=1,598)

| **Ethnicity** | **Number of Women** |
| --- | --- |
| British | 1497 (93.68%) |
| Any other white background | 42 (2.63%) |
| Irish | 25 (1.56%) |
| Chinese | 9 (0.56%) |
| Other ethnic group | 5 (0.31%) |
| Caribbean | 5 (0.31%) |
| Indian | 3 (19%) |
| Pakistani | 3 (19%) |
| African | 3 (19%) |
| Any other mixed background | 3 (19%) |
| White and Black Caribbean | 2 (13%) |
| Any other Asian background | 1 (0.06%) |
| White and Black African | 0 (0%) |

Information on ethnicity was collected based on self-reports using a touchscreen questionnaire at the UK Biobank Assessment Centre.
