# Supplementary material for: A Case for estradiol: younger brains in women with earlier menarche and later menopause
Source: Gigascience. 2025 May 23;14:giaf060. doi: 10.1093/gigascience/giaf060 (PMC12099614; doi:10.1093/gigascience/giaf060)
Supplement: giaf060_GIGA-D-24-00418_original_submission [file giaf060_giga-d-24-00418_original_submission.pdf]

GigaScience

A Case for Estradiol: Younger Brains in Women with Earlier Menarche and Later Menopause

--Manuscript Draft--

Manuscript Number:	GIGA-D-24-00418
Full Title:	A Case for Estradiol: Younger Brains in Women with Earlier Menarche and Later Menopause
Article Type:	Research
Funding Information:	Helse Sør-Øst RHF (2023037, 2022103) — Dr. Claudia Barth
Abstract:	The transition to menopause is marked by a gradual decrease of estradiol. At the same time, the risk of dementia increases around menopause and it stands to reason that estradiol (or the lack thereof) plays a role for the development of dementia and other age-related neuropathologies. Here we investigated if there is a link between brain aging and estradiol-associated events, such as menarche and menopause. For this purpose, we applied a well-validated machine learning approach in a sample of 1,006 postmenopausal women who were scanned twice approximately two years apart. We observed less brain aging in women with an earlier menarche, a later menopause, and a longer reproductive span (i.e., the time interval between menarche and menopause). These effects were evident both cross-sectionally and longitudinally, which supports the notion that estradiol contributes to brain preservation. However, further research is required because no direct measures of estradiol were obtained and effects were small overall.
Corresponding Author:	Eileen Luders<br>Uppsala Universitet<br>Uppsala, SWEDEN
Corresponding Author Secondary Information:	
Corresponding Author's Institution:	Uppsala Universitet
Corresponding Author's Secondary Institution:	
First Author:	Eileen Luders
First Author Secondary Information:	
Order of Authors:	Eileen Luders
	Inger Sundström Poromaa
	Claudia Barth
	Christian Gaser
Order of Authors Secondary Information:	
Additional Information:	
Question	Response
Are you submitting this manuscript to a special series or article collection?	No
**Experimental design and statistics**<br><br>Full details of the experimental design and statistical methods used should be given in the Methods section, as detailed in our	Yes

Minimum Standards Reporting Checklist. Information essential to interpreting the data presented should be made available in the figure legends.<br><br>Have you included all the information requested in your manuscript?	
**Resources**<br><br>A description of all resources used, including antibodies, cell lines, animals and software tools, with enough information to allow them to be uniquely identified, should be included in the Methods section. Authors are strongly encouraged to cite Research Resource Identifiers (RRIDs) for antibodies, model organisms and tools, where possible.<br><br>Have you included the information requested as detailed in our Minimum Standards Reporting Checklist?	Yes
**Availability of data and materials**<br><br>All datasets and code on which the conclusions of the paper rely must be either included in your submission or deposited in publicly available repositories (where available and ethically appropriate), referencing such data using a unique identifier in the references and in the "Availability of Data and Materials" section of your manuscript.<br><br>Have you have met the above requirement as detailed in our Minimum Standards Reporting Checklist?	Yes
GigaScience has policies and guidelines in place for the use of generative AI-writing tools such as ChatGPT. If you have used such writing tools to assist with writing the manuscript this must be declared and cited in the text. Authors should not list AI-writing tools and other	No

AI-assisted technologies as an author or co-author and should acknowledge that they are fully responsible for text generated or refined by AI-writing tools.<br><br>A summary of use (particularly in the introduction or among methods) needs to be included at the end of the paper, and the outputs should also be included as a supplementary file hosted in GigaDB or other open repositories. Please read our guidelines for more information. <br>By submitting to GigaScience, you are aware of the journal's AI-writing tools policy, and if you have declared use of such tools below, you have acknowledged this where appropriate in your manuscript and have made a summary of use and outputs available. <br><br>**AI-assisted writing tools have been used in the preparation of this manuscript?<br>** 	

Manuscript
Click here to access/download;Manuscript;O_Submitted Manuscript_updated.docx

Title:

A Case for Estradiol: Younger Brains in Women with Earlier Menarche and Later Menopause

Authors and Affiliations:

Eileen Luders[1,2,3,4*] | Inger Sundström Poromaa[1] | Claudia Barth[5] | Christian Gaser[6,7,8]

[1]Department of Women's and Children's Health, Uppsala University, Uppsala, Sweden
[2]Swedish Collegium for Advanced Study (SCAS), Uppsala, Sweden
[3]School of Psychology, University of Auckland, Auckland, New Zealand
[4]Laboratory of Neuro Imaging, School of Medicine, University of Southern California, Los Angeles, USA
[5]Department of Psychiatric Research, Diakonhjemmet Hospital, Oslo, Norway
[6]Department of Psychiatry and Psychotherapy, Jena University Hospital, Jena, Germany
[7]Department of Neurology, Jena University Hospital, Jena, Germany
[8]German Center for Mental Health (DZPG)

**Correspondence should be addressed to:*

Eileen Luders, Ph.D.

Department of Women's and Children's Health, Uppsala University, Uppsala, Sweden
Email: eileen.lueders@uu.se

Number of Words in Abstract:	**≤250**
Number of Figures:	**3**
Number of Tables:	**3**
Number of Pages:	**21**
Supplemental Material:	**2 Tables**

Date of Submission: **September 27th, 2024**

Abstract

The transition to menopause is marked by a gradual decrease of estradiol. At the same time, the risk of dementia increases around menopause and it stands to reason that estradiol (or the lack thereof) plays a role for the development of dementia and other age-related neuropathologies. Here we investigated if there is a link between brain aging and estradiol-associated events, such as menarche and menopause. For this purpose, we applied a well-validated machine learning approach in a sample of 1,006 postmenopausal women who were scanned twice approximately two years apart. We observed less brain aging in women with an earlier menarche, a later menopause, and a longer reproductive span (i.e., the time interval between menarche and menopause). These effects were evident both cross-sectionally and longitudinally, which supports the notion that estradiol contributes to brain preservation. However, further research is required because no direct measures of estradiol were obtained and effects were small overall.

Keywords:

brain age; estradiol; machine learning; menarche; MRI; menopause; structural neuroimaging

Background

Estradiol is the most potent and prevalent form of estrogen during the reproductive life of a woman[1]. Generally speaking, estradiol levels start increasing just before the first menstrual period (menarche) and then plateau on a high level until they start decreasing during perimenopause. After the final menstrual period (i.e., menopause) estradiol levels decrease further and eventually reach plateauing low levels during postmenopause[2]. The risk for dementia in women is known to increase around menopause[3-6] and thus it stands to reason that estradiol plays a role for the development of dementia and other age-related neuropathologies. However, scientific studies focused on specific phases (e.g., menarche, pregnancy, menopause) or interventions (oral contraceptives, estrogen modulation therapy, estrogen replacement therapy) paint a rather complex picture, with no consistent evidence that more (estradiol) is always beneficial[4,7-22]. For example, with particular respect to menarche and menopause, both early onset and late onset have been shown to increase the risk for dementia or to be positively associated with brain aging and cognitive functioning[4,7-14,18-20].

To further advance this field of research, the current study set out to determine if there is a link between a woman's estimated brain age (a biological marker of brain health[23]) and the reproductive span (i.e., the interval between menarche and menopause when estradiol levels are high). If a lack of estradiol is among the driving factors for diminished brain health later in life, brain age and reproductive span should be inversely related (negative correlation). To be able to relate our findings to others in the literature[7-9] and to provide a frame of reference for future studies, we additionally investigated if there is a significant link between estimated brain age and the age at menarche as well as the age at menopause. Assuming a neuroprotective

effect of estradiol, we expected that a lower brain age would be linked to an earlier menarche (positive correlation) and to a later menopause (negative correlation). Importantly, our study comprises both cross-sectional and longitudinal components, with follow-up data acquired approximately two years after the initial brain scan. This constitutes a critical extension to existing studies from the UK biobank with a related focus as those studies are solely cross-sectional in nature [8,9,11,1718,19].

To estimate brain age, we used structural brain images and a well-validated high-dimensional pattern recognition approach, as detailed elsewhere [24,25]. Briefly, the difference between the estimated brain age and the chronological age yields a so-called brain age gap estimate (BrainAGE) in years. The BrainAGE index is negative if a brain is estimated younger than its chronological age; it is positive if a brain is estimated older than its chronological age. For example, a 50-year-old woman with a BrainAGE index of -3 years shows the aging pattern of a 47-year-old. The BrainAGE algorithm has been shown to be robust and reliable across datasets, age-ranges, and scanner types[24,26]; it has been successfully applied in a wide range of studies[24,25,27-29] including those capturing hormonal changes in women[30,31]. Moreover, the BrainAGE index has been demonstrated to work as a predictor of dementia as well as age-related cognitive decline[27,32].

Materials and Methods

Data Description

The study is based on a carefully selected sample of 1,006 postmenopausal women from the UK Biobank (https://www.ukbiobank.ac.uk/) which was accessed under application number

#41655. The UK Biobank is a biomedical database and research resource that contains genetic, lifestyle and health information from half a million people. The UK Biobank holds the ethical approval from the North West Multi-Centre Research Ethics Committee (MREC) and is in possession of the informed consents. Written informed consent was obtained from all participants. Inclusion criteria for the current study were women with available longitudinal data as well as information on age at menarche and age at menopause. Exclusion criteria for the current study were pre-existing neurological or psychiatric diagnoses as per UK Biobank data fields #41202-0.0 to #41202-0.78. In addition, to further increase the homogeneity of the sample, we excluded women whose age at menarche was younger than 10 or older than 18, or whose age at menopause was younger than 45 or older than 60. This resulted in a final sample size of 1,006 women. **Table 1** provides information on this final sample; **Figure 1** summarizes the steps related to the sample selection. For each woman, one initial brain scan and one follow-up brain scan – approximately two years apart (mean ± SD: 2.35 ± 6.12 years) – were obtained *after* menopause. These T1-weighted brain images were acquired on a 3 Tesla Siemens Skyra scanner using a 32-channel head coil, as described elsewhere[33] (see also: http://biobank.ctsu.ox.ac.uk/crystal/crystal/docs/bmri_V4_23092014.pdf).

– Table 1 –

– Figure 1 –

Data Analyses

Using the aforementioned T1-weighted images, we applied a number of processing routines implemented in the CAT12 toolbox[34] (version 12.8), which resulted in bias-corrected, spatially normalized, and tissue-classified brain images, as detailed elsewhere[24,31]. The normalized gray and white matter partitions were smoothed using a 4 and 8 mm full-width-at-half-maximum (FWHM) Gaussian kernel, and image resolution was set to 4 and 8 mm. For further data reduction, we applied a principal component analysis (PCA) using singular value decomposition to all the models using n–1 PCA components (n = minimum of voxel number or sample size). For the estimation of the BrainAGE index, we employed a Gaussian Process Regression (GPR) that uses a linear covariance function, a constant mean function, and a Gaussian likelihood function; hyperparameters were set to 100 for the constant mean function and to -1 for the likelihood function[35]. As training data, we selected 3,046 individuals from the UK Biobank where two time points were available and applied a 10-fold validation approach separately for the initial and follow-up brain scan. To estimate the individual brain ages, eight models based on the aforementioned sets of images (i.e., gray matter/white matter, 4 mm/8 mm Gaussian kernel, and 4 mm/8 mm image resolution) were combined using a general linear model where the weights of the models were derived by maximizing the variance to the parameter of interest (e.g., menopause). The difference between the resulting estimated brain age and the chronological age was then calculated as the BrainAGE index (in years).

Statistical Analyses

Main Analyses

After computing the BrainAGE index for all 1,006 women at initial and follow-up scan, we first removed the linear age trend that is typically seen in BrainAGE estimation. Then, we conducted two analysis streams using linear regressions in Matlab (version R2023b), one cross-sectional and one longitudinal. For all analyses, alpha was set at 0.05 (two-tailed). For the cross-sectional stream, we tested if there is a significant link between the BrainAGE index at the initial brain scan and the reproductive span. In addition, we tested if there is a significant link between the BrainAGE index at the initial brain scan and the age at menarche as well as the age at menopause. For the longitudinal stream, we first subtracted the BrainAGE index at the initial brain scan from the BrainAGE index at the follow-up brain scan, which resulted in a Δ BrainAGE index for each individual. This method, often referred to as "change score" analysis, produces statistical results that are comparable to those resulting from a repeated-measures ANOVA with two time points. Using the Δ BrainAGE index, we then tested for significant links with the reproductive span, the age at menarche, and the age at menopause.

Sensitivity Analyses

The aforementioned main analyses were repeated while accounting for potential confounds known to affect brain health. More specifically, we removed the variance associated with the number of live births[36] (UK Biobank data field #2734), hormone replacement therapy[7] (#2814), hysterectomy[37] (#3591), bilateral oophorectomy[37] (#834), body mass index[38] (#21001), diastolic and systolic blood pressure[39] (#4079 and #4080), diabetes[40] (#2443), education[41] (#6138), income[42] (#738), and a composite lifestyle factor[43]. The latter was expressed as a general lifestyle score that was calculated based on a number of factors (see **Supplemental Table 1**),

known to increase / decrease the risk of adverse cardiovascular events. Since not all women had information on all potential confounds (see **Supplemental Table 2**), we applied an imputation method using the Matlab function 'fillmissing'. That is, missing entries were replaced with the corresponding values from the nearest neighbor rows, calculated based on the pairwise Euclidean distance between rows. Imputation was applied to up to 295 women, depending on the potential confound. For the cross-sectional stream, we tested if there is a significant link between the BrainAGE index at the initial brain scan and the reproductive span (age at menarche and age at menopause, respectively). Likewise, for the longitudinal stream, we tested if there is a significant link between the Δ BrainAGE index and the reproductive span (age at menarche and age at menopause, respectively).

Results

Main Analyses

As shown in **Figure 2** (left), our cross-sectional analyses revealed a significant negative association between BrainAGE and the reproductive span. In other words, brains of women with longer reproductive spans were estimated younger than brains of women with shorter reproductive spans. As also shown in **Figure 2** (right), there was a significant positive association between BrainAGE and age at menarche (i.e., the earlier the menarche, the younger the brain) and a significant negative association between BrainAGE and age at menopause (i.e., the later the menopause, the younger the brain). As shown in **Table 2** (main analyses), effect sizes were small[44], with r-values at -0.11, 0.14, and -0.09 for reproductive span, menarche, and

menopause, respectively). The slopes of the regression indicate different rates of change for menarche and menopause (0.32 and -0.10, respectively). More specifically, for each year younger at menarche, brains are estimated 0.32 years younger (which corresponds to 3.2 years younger for each 10 years). In contrast, for each year older at menopause, brains are estimated 0.1 year younger (which corresponds to 1 year younger for each 10 years).

– Figure 2 –

– Table 2 –

As shown in **Figure 3** and **Table 3** (main analyses), our longitudinal findings confirm the observed cross-sectional relationships. More specifically, Δ BrainAGE was negatively linked to reproductive span and menopause, and positively linked to age at menarche. All associations were significant. Again, effect sizes were small, with r-values at -0.12, 0.06, and -0.12 for reproductive span, menarche, and menopause, respectively. The slopes of the regression are still somewhat different for menarche and menopause (0.08 and -0.06, respectively), albeit more similar than in the cross-sectional analysis: For each year younger at menarche, brains are estimated 0.08 years younger (0.8 years over 10 years), whereas for each year older at menopause, brains are estimated 0.06 years younger (0.6 years over 10 years).

– Figure 3 –

– Table 3 –

Sensitivity Analyses

The results described above remained comparable when removing the variance associated with the number of live births, hormone replacement therapy, hysterectomy, bilateral oophorectomy, body mass index, diastolic and systolic blood pressure, diabetes, education, income, and a composite lifestyle factor. In other words, when examining the association between BrainAGE and reproductive span, we observed a negative association. Likewise, there was a positive association between BrainAGE and age at menarche and a negative association between BrainAGE and age at menopause. The effects were significant for reproductive span, menarche, and menopause for the cross-sectional analyses (see **Table 2**; Sensitivity Analyses), and for reproductive span and menopause for the longitudinal (see **Table 3**; Sensitivity Analyses).

Discussion

Here we assessed links between estimated brain age and milestones in a woman's reproductive life in a well-powered sample of more than a thousand postmenopausal women. We detected less brain aging in women with longer reproductive spans, earlier menarche, and later menopause.

Correspondence with Previous Findings

Our findings are in line with the outcomes of other studies suggesting a longer reproductive span [8,10,13], an earlier menarche[13,14], as well as a later menopause [4,8,10-12] to be associated with a

lower risk of developing dementia or better retained cognitive function. Furthermore, given that the BrainAGE index is based on the weighted distribution of gray and whiter matter tissue in the brain, our findings are also in agreement with reports of lower brain volumes as well as higher rates of brain tissue loss during menopause compared to premenopause or in postmenopausal women compared to premenopausal women[45-47]. In addition, our findings agree with observed effects across the menstrual cycle linking high estradiol levels at ovulation to lower BrainAGE estimates[30]. Altogether, the outcomes of our study seem to suggest that estradiol contributes to brain health, which is in agreement with other studies reporting positive effects of estradiol on brain health and cognition within the framework of aging and/or menopausal hormone therapy[48-52].

Menarche versus Menopause

The outcomes of the main analyses indicate that both, an earlier menarche and a later menopause, are significantly associated with less brain aging. However, menarche and menopause differ with respect to the strength of their relationship with age (which is reflected in the correlation coefficient) and their rate of change with age (which is reflected in the slope of the regression line). This might indicate somewhat different underlying biological mechanisms and/or confounds for menarche and menopause. For example, during menopause, in addition to decreasing levels of estradiol, increasing levels of follicle-stimulating hormones may cause an accelerated deposition of amyloid-β and Tau[53], which enhances brain atrophy. Moreover, menopause is marked by disadvantageous alterations in cytokine and T cell profiles[54] , which are linked to an enhanced inflammation. Alternatively, the less strong link

pertaining to menarche could also reflect the fact that, later in life, it is probably more challenging to accurately remember the onset of menarche than the onset of menopause.

Potential Implications

Given that estradiol levels start decreasing during perimenopause and further decrease after menopause, our findings may explain why the risk for dementia in women is known to increase around menopause[3-6] and why there is an increased age-independent prevalence of Alzheimer's disease in women compared to men[52]. Moreover, our findings seem to support to the concept of the "window of opportunity", spanning the years leading up to menopause to the years immediately after menopause, where health interventions (e.g., menopausal hormone treatment) may combat the increased risk for Alzheimer's disease in some women[5,55-57]. In fact, several large-scale projects have investigated the effects of menopausal hormone treatment on cognitive function and Alzheimer's risk, but results are inconclusive (potentially relevant modulators of treatment outcomes are discussed elsewhere[48,58-63]).

The current findings seem to suggest a protective effect of estradiol and as such seem promising in the framework of prevention and intervention. However, further research is required, as the effect sizes for the observed associations were small (albeit smaller effect sizes are not uncommon in studies with larger sample sizes [64]) and other factors, such as genetics, life style, or hormones other than estradiol, could play a greater (or at least an additional) role in preserving brain health[2,65,66]. Moreover, our study did not measure estradiol directly, and links between estradiol and brain aging seem to be rather complex as indicated by the outcomes of other studies. For example, it was reported that, compared to no exposure or no

dose, exposure to low concentrations of estradiol or low doses of estrogen enhanced neuronal survival and increased anti-inflammatory markers (i.e., positive links), while exposure to high concentrations of estradiol as well as high doses of estrogen had the opposite effect (i.e., negative links)[21,22]. Another study reported U-shaped curves suggesting that both early and late menarche are associated with an increased risk for dementia (i.e., positive and negative links)[8]. And yet another study reported either negative links or missing links between age at menarche and brain aging depending on the potential confounds accounted for[7]. Interestingly, this latter study also reported that, in carriers of the apolipoprotein E type 4 allele (APOE e4), higher levels of estradiol at menopause were associated with increased brain aging (positive link). In contrast, in non-carriers, higher levels of estradiol at menopause were associated with decreased brain aging (negative link)[7].

Conclusion

Our study revealed less brain aging in women with a larger reproductive span, earlier menarche, and later menopause. Thus, sex hormones – potentially estradiol – may contribute to brain health. However, follow-up research is required because effects in the current study were small, estradiol was not directly examined, and female brain health may be modulated by other factors than estradiol. Moreover, to paint a more complete picture and expand an understudied field research, future research focussing on specific time frames surrounding menopause, such as perimenopause (i.e., the time preceding the final menstrual period) or early postmenopause (e.g., the initial year after menopause) versus late menopause (e.g., ten years after menopause) would be desirable. Last but not least, the UK Biobank is biased

towards healthy[65] and more socioeconomically privileged individuals[65] with a predominant white ethnic background[65], which affects the generalizability of the findings.

Acknowledgments

EL was supported by the Swedish Collegium for Advanced Study (SCAS) and Erling-Persson Family Foundation. The data of the UK Biobank were accessed under application number #41655. The article was composed using the STROBE cohort checklist[66].

Funding

CB received funding from the South-Eastern Norway Regional Health Authority (2023037, 2022103).

Data Availability

The individual-level proxy measures obtained from the prediction models in this work will be shared after publication in agreement with UK Biobank regulations. The input data are available to other researchers through the UK Biobank's controlled access scheme [1]. The procedure to apply for access [2] requires registering with the UK Biobank and completion of an application form detailing:

- A summary of the planned research
- The UK Biobank data fields required for the project
- A description of derivatives (data, variables) generated by the project

[1] Link to UK Biobank website: http://www.ukbiobank.ac.uk

[2] Link to UK Biobank access procedure: https://www.ukbiobank.ac.uk/enable-your-research/apply-for-access.

Availability of Source Code and Requirements

Project name: BrainAGE-UKBiobank

Project home page: https://github.com/ChristianGaser/BrainAGE-UKBiobank

Operating system(s): Platform independent

Programming language: Matlab

Other requirements: SPM12, CAT12, BrainAGE

License: GNU GPL-3.0

Competing Interests

The authors declare that the research was conducted in the absence of any commercial or financial relationships that could be construed as a potential conflict of interest.

References

1. Thomas, M.P. & Potter, B.V. The structural biology of oestrogen metabolism. *The Journal of steroid biochemistry and molecular biology* **137**, 27-49 (2013).
2. Barth, C., Crestol, A., de Lange, A.G. & Galea, L.A.M. Sex steroids and the female brain across the lifespan: insights into risk of depression and Alzheimer's disease. *Lancet Diabetes Endocrinol* **11**, 926-941 (2023).
3. Farrer, L.A.*, et al.* Effects of age, sex, and ethnicity on the association between apolipoprotein E genotype and Alzheimer disease. A meta-analysis. APOE and Alzheimer Disease Meta Analysis Consortium. *JAMA* **278**, 1349-1356 (1997).
4. Brinton, R.D., Yao, J., Yin, F., Mack, W.J. & Cadenas, E. Perimenopause as a neurological transition state. *Nat Rev Endocrinol* **11**, 393-405 (2015).
5. Mosconi, L.*, et al.* Increased Alzheimer's risk during the menopause transition: A 3-year longitudinal brain imaging study. *PLoS One* **13**, e0207885 (2018).
6. Rahman, A.*, et al.* Sex-driven modifiers of Alzheimer risk: A multimodality brain imaging study. *Neurology* **95**, e166-e178 (2020).
7. de Lange, A.G.*, et al.* Women's brain aging: Effects of sex-hormone exposure, pregnancies, and genetic risk for Alzheimer's disease. *Hum Brain Mapp* **41**, 5141-5150 (2020).
8. Gong, J., Harris, K., Peters, S.A.E. & Woodward, M. Reproductive factors and the risk of incident dementia: A cohort study of UK Biobank participants. *PLoS Med* **19**, e1003955 (2022).
9. Jani, M.*, et al.* Birth outcomes, puberty onset, and obesity as long-term predictors of biological aging in young adulthood. *Front Nutr* **9**, 1100237 (2022).
10. Lindseth, L.R.S.*, et al.* Associations between reproductive history, hormone use, APOE epsilon4 genotype and cognition in middle- to older-aged women from the UK Biobank. *Front Aging Neurosci* **14**, 1014605 (2022).
11. Kuh, D., Cooper, R., Moore, A., Richards, M. & Hardy, R. Age at menopause and lifetime cognition: Findings from a British birth cohort study. *Neurology* **90**, e1673-e1681 (2018).
12. McLay, R.N., Maki, P.M. & Lyketsos, C.G. Nulliparity and late menopause are associated with decreased cognitive decline. *J Neuropsychiatry Clin Neurosci* **15**, 161-167 (2003).
13. Karim, R.*, et al.* Effect of Reproductive History and Exogenous Hormone Use on Cognitive Function in Mid- and Late Life. *J Am Geriatr Soc* **64**, 2448-2456 (2016).
14. Ryan, J., Carriere, I., Scali, J., Ritchie, K. & Ancelin, M.L. Life-time estrogen exposure and cognitive functioning in later life. *Psychoneuroendocrinology* **34**, 287-298 (2009).
15. Branigan, G.L., Soto, M., Neumayer, L., Rodgers, K. & Brinton, R.D. Association Between Hormone-Modulating Breast Cancer Therapies and Incidence of Neurodegenerative Outcomes for Women With Breast Cancer. *JAMA Netw Open* **3**, e201541 (2020).
16. Comasco, E., Frokjaer, V.G. & Sundstrom-Poromaa, I. Functional and molecular neuroimaging of menopause and hormone replacement therapy. *Frontiers in neuroscience* **8**, 388 (2014).
17. Nabulsi, L.*, et al.* Exogenous Sex Hormone Effects on Brain Microstructure in Women: A diffusion MRI Study in the UK Biobank. *bioRxiv : the preprint server for biology*, 2020.2009.2018.304154 (2023).

18. Georgakis, M.K., *et al.* Age at menopause and duration of reproductive period in association with dementia and cognitive function: A systematic review and meta-analysis. *Psychoneuroendocrinology* **73**, 224-243 (2016).
19. Ambikairajah, A., Tabatabaei-Jafari, H., Hornberger, M. & Cherbuin, N. Age, menstruation history, and the brain. *Menopause* **28**, 167-174 (2020).
20. Gilsanz, P., *et al.* Reproductive period and risk of dementia in a diverse cohort of health care members. *Neurology* **92**, e2005-e2014 (2019).
21. Prestwood, K.M., Unson, C., Kulldorff, M. & Cushman, M. The effect of different doses of micronized 17beta-estradiol on C-reactive protein, interleukin-6, and lipids in older women. *J Gerontol A Biol Sci Med Sci* **59**, 827-832 (2004).
22. Chen, S., Nilsen, J. & Brinton, R.D. Dose and temporal pattern of estrogen exposure determines neuroprotective outcome in hippocampal neurons: therapeutic implications. *Endocrinology* **147**, 5303-5313 (2006).
23. Franke, K. & Gaser, C. Ten Years of BrainAGE as a Neuroimaging Biomarker of Brain Aging: What Insights Have We Gained? *Front Neurol* **10**, 789 (2019).
24. Franke, K., Ziegler, G., Kloppel, S. & Gaser, C. Estimating the age of healthy subjects from T1-weighted MRI scans using kernel methods: exploring the influence of various parameters. *Neuroimage* **50**, 883-892 (2010).
25. Franke, K., Luders, E., May, A., Wilke, M. & Gaser, C. Brain maturation: Predicting individual BrainAGE in children and adolescents using structural MRI. *Neuroimage* **63**, 1305-1312 (2012).
26. Kalc, P., Dahnke, R., Hoffstaedter, F. & Gaser, C. BrainAGE: Revisited and reframed machine learning workflow. *Hum Brain Mapp* **45**, e26632 (2024).
27. Gaser, C., Franke, K., Kloppel, S., Koutsouleris, N. & Sauer, H. BrainAGE in Mild Cognitive Impaired Patients: Predicting the Conversion to Alzheimer's Disease. *PLoS One* **8**, e67346 (2013).
28. Franke, K., Ristow, M. & Gaser, C. Gender-specific impact of personal health parameters on individual brain aging in cognitively unimpaired elderly subjects. *Front Aging Neurosci* **6**, 94 (2014).
29. Luders, E., Cherbuin, N. & Gaser, C. Estimating brain age using high-resolution pattern recognition: Younger brains in long-term meditation practitioners. *NeuroImage* **134**, 508-513 (2016).
30. Franke, K., Hagemann, G., Schleussner, E. & Gaser, C. Changes of individual BrainAGE during the course of the menstrual cycle. *NeuroImage* **115**, 1-6 (2015).
31. Luders, E., *et al.* Potential Brain Age Reversal after Pregnancy: Younger Brains at 4-6Weeks Postpartum. *Neuroscience* **386**, 309-314 (2018).
32. Giannakopoulos, P., *et al.* Alzheimer resemblance atrophy index, BrainAGE, and normal pressure hydrocephalus score in the prediction of subtle cognitive decline: added value compared to existing MR imaging markers. *Eur Radiol* (2022).
33. Alfaro-Almagro, F., *et al.* Image processing and Quality Control for the first 10,000 brain imaging datasets from UK Biobank. *NeuroImage* **166**, 400-424 (2018).
34. Gaser, C., Dahnke, R., Thompson, P.M., Kurth, F. & Luders, E. CAT – A Computational Anatomy Toolbox for the Analysis of Structural MRI Data. *bioRxiv* **2022.06.11.495736**(2022).

35. Rasmussen, C.E.W., C. K. I. Gaussian Processes for Machine Learning. *MIT Press* (2006).
36. de Lange, A.G.*, et al.* Population-based neuroimaging reveals traces of childbirth in the maternal brain. *Proc Natl Acad Sci U S A* **116**, 22341-22346 (2019).
37. Rocca, W.A., Grossardt, B.R., Shuster, L.T. & Stewart, E.A. Hysterectomy, oophorectomy, estrogen, and the risk of dementia. *Neurodegener Dis* **10**, 175-178 (2012).
38. Tungler, A.*, et al.* Body mass index but not genetic risk is longitudinally associated with altered structural brain parameters. *Scientific reports* **11**, 24246 (2021).
39. Cherbuin, N.*, et al.* Optimal Blood Pressure Keeps Our Brains Younger. *Front Aging Neurosci* **13**, 694982 (2021).
40. Antal, B.*, et al.* Type 2 diabetes mellitus accelerates brain aging and cognitive decline: Complementary findings from UK Biobank and meta-analyses. *Elife* **11**(2022).
41. Chan, M.Y.*, et al.* Long-term prognosis and educational determinants of brain network decline in older adult individuals. *Nat Aging* **1**, 1053-1067 (2021).
42. Busby, N.*, et al.* Lower socioeconomic status is associated with premature brain aging. *Neurobiology of aging* **130**, 135-140 (2023).
43. Foster, H.M.E.*, et al.* The effect of socioeconomic deprivation on the association between an extended measurement of unhealthy lifestyle factors and health outcomes: a prospective analysis of the UK Biobank cohort. *Lancet Public Health* **3**, e576-e585 (2018).
44. Cohen, J. A power primer. *Psychol.Bull.* **112**, 155-159 (1992).
45. Goto, M.*, et al.* Accelerated hippocampal volume reduction in post-menopausal women: an additional study with Atlas-based method. *Radiol Phys Technol* **4**, 185-188 (2011).
46. Goto, M.*, et al.* 3 Tesla MRI detects accelerated hippocampal volume reduction in postmenopausal women. *J Magn Reson Imaging* **33**, 48-53 (2011).
47. Lu, W.*, et al.* Grey matter differences associated with age and sex hormone levels between premenopausal and perimenopausal women: A voxel-based morphometry study. *J Neuroendocrinol* **30**, e12655 (2018).
48. Manly, J.J.*, et al.* Endogenous estrogen levels and Alzheimer's disease among postmenopausal women. *Neurology* **54**, 833-837 (2000).
49. Boccardi, M.*, et al.* Effects of hormone therapy on brain morphology of healthy postmenopausal women: a Voxel-based morphometry study. *Menopause* **13**, 584-591 (2006).
50. Ghidoni, R.*, et al.* Effects of estrogens on cognition and brain morphology: involvement of the cerebellum. *Maturitas* **54**, 222-228 (2006).
51. Kim, T.H., Kim, B., Kim, Y.R., Jeong, C.W. & Lee, Y.H. Gray matter differences associated with menopausal hormone therapy in menopausal women: a DARTEL-based VBM study. *Scientific reports* **13**, 1401 (2023).
52. Depypere, H.*, et al.* Menopause hormone therapy significantly alters pathophysiological biomarkers of Alzheimer's disease. *Alzheimers Dement* **19**, 1320-1330 (2023).
53. Xiong, J.*, et al.* FSH blockade improves cognition in mice with Alzheimer's disease. *Nature* **603**, 470-476 (2022).
54. Mishra, A. & Brinton, R.D. Inflammation: Bridging Age, Menopause and APOEepsilon4 Genotype to Alzheimer's Disease. *Front Aging Neurosci* **10**, 312 (2018).

55. Scheyer, O.*, et al.* Female Sex and Alzheimer's Risk: The Menopause Connection. *J Prev Alzheimers Dis* **5**, 225-230 (2018).
56. Davey, D.A. Alzheimer's disease, dementia, mild cognitive impairment and the menopause: a 'window of opportunity'? *Womens Health (Lond)* **9**, 279-290 (2013).
57. Mishra, A.*, et al.* A tale of two systems: Lessons learned from female mid-life aging with implications for Alzheimer's prevention & treatment. *Ageing Res Rev* **74**, 101542 (2022).
58. Middleton, L.E. & Yaffe, K. Promising strategies for the prevention of dementia. *Arch Neurol* **66**, 1210-1215 (2009).
59. Marder, K. & Sano, M. Estrogen to treat Alzheimer's disease: too little, too late? So what's a woman to do? *Neurology* **54**, 2035-2037 (2000).
60. Resnick, S.M. & Henderson, V.W. Hormone therapy and risk of Alzheimer disease: a critical time. *JAMA* **288**, 2170-2172 (2002).
61. Yaffe, K. Estrogens, selective estrogen receptor modulators, and dementia: what is the evidence? *Ann N Y Acad Sci* **949**, 215-222 (2001).
62. Yaffe, K., Haan, M., Byers, A., Tangen, C. & Kuller, L. Estrogen use, APOE, and cognitive decline: evidence of gene-environment interaction. *Neurology* **54**, 1949-1954 (2000).
63. Nerattini, M.*, et al.* Systematic review and meta-analysis of the effects of menopause hormone therapy on risk of Alzheimer's disease and dementia. *Front Aging Neurosci* **15**, 1260427 (2023).
64. Marek, S.*, et al.* Reproducible brain-wide association studies require thousands of individuals. *Nature* **603**, 654-660 (2022).
65. Fry, A.*, et al.* Comparison of Sociodemographic and Health-Related Characteristics of UK Biobank Participants With Those of the General Population. *Am J Epidemiol* **186**, 1026-1034 (2017).
66. von Elm, E.*, et al.* The Strengthening the Reporting of Observational Studies in Epidemiology (STROBE) statement: guidelines for reporting observational studies. *J Clin Epidemiol* **61**, 344-349 (2008).

Figure Legends

Figure 1. Flowchart of sample selection.

Figure 2. Correlations with BrainAGE at the initial brain scan. The x-axes show the reproductive span (age, respectively) in years. Of note, age in the UK Biobank has been rounded to the year, so we added a small random jitter to the x-axes to give a better overview about the age distribution. The y-axes show the BrainAGE index in years, with negative values indicating that brains are estimated younger than their chronological age and positive values indicating that brains are estimated older than their chronological age. Panel A displays a negative link between the BrainAGE index and the reproductive span (the longer the reproductive span, the younger the estimated brain age). Panel B displays a positive link between the BrainAGE index and the age at menarche (the earlier the onset of menarche, the younger the estimated brain age). Panel C displays a negative link between the BrainAGE index and the age at menopause (the later the onset of menopause, the younger the estimated brain age). Hot colors in the density plot indicate a larger overlay of measures; cool colors indicate a smaller overlay. The shaded band is the 95% confidence interval.

Figure 3. Correlations with BrainAGE over 2.35 years (Δ BrainAGE). Panel A displays a negative link between the BrainAGE index and reproductive span (the longer the reproductive span, the smaller the estimated brain age). Panel B displays a positive link between the BrainAGE index and the age at menarche (the earlier the onset of menarche, the younger the estimated brain age). Panel C displays a negative link between the BrainAGE index and the age at menopause (the later the onset of menopause, the younger the estimated brain age). Hot colors in the density plot indicate a larger overlay of measures; cool colors indicate a smaller overlay. The shaded band is the 95% confidence interval.

Tables

Table 1. Sample characteristics

Variable	Descriptive Statistics
Age at the initial brain scan	mean ± SD: 63.20 ± 6.42 years
Age at the follow-up brain scan	mean ± SD: 65.54 ± 6.37 years
Age at menarche	mean ± SD: 13.02 ± 1.53 years
Age at menopause	mean ± SD: 51.41 ± 3.23 years
Reproductive span	mean ± SD: 38.39 ± 3.55 years
Number of live births	mean ± SD: 1.75 ± 1.16
Number of women with hormone replacement therapy	yes: 306 | no: 700
Number of women with hysterectomy	yes: 48 | no: 958
Number of women with bilateral oophorectomy	yes: 37 | no: 969

SD = standard deviation

Table 2. Associations with BrainAGE at the initial brain scan

	**Main Analyses**					**Sensitivity Analyses***				
	**R^2**	**r**	**p**	**Slope**	**95% Confidence Interval**	**R^2**	**r**	**p**	**Slope**	**95% Confidence Interval**
Reproductive Span	0.01	-0.11	<0.001	-0.11	-0.17 to -0.05	0.01	-0.11	<0.001	-0.11	-0.17 to -0.05
Age at Menarche	0.02	0.14	<0.001	0.32	0.18 to 0.46	0.02	0.14	<0.001	0.33	0.19 to 0.47
Age at Menopause	0.01	-0.09	<0.005	-0.10	-0.17 to -0.03	0.01	-0.09	<0.01	-0.09	-0.16 to -0.03

*While removing the variance associated with the number of live births, hormone replacement therapy, hysterectomy, bilateral oophorectomy, body mass index, diastolic and systolic blood pressure, diabetes, education, income, and a composite lifestyle factor.

Table 3. Associations with changes in BrainAGE over 2.35 years

	Main Analyses					Sensitivity Analyses*				
	R^2	r	p	Slope	95% Confidence Interval	R^2	r	p	Slope	95% Confidence Interval
Reproductive Span	0.01	-0.12	<0.001	-0.06	-0.10 to -0.03	0.01	-0.11	<0.001	-0.06	-0.09 to -0.03
Age at Menarche	<0.01	0.06	<0.05	0.08	0.0 to 0.16	<0.01	0.06	n.s.	0.08	0.0 to 0.16
Age at Menopause	0.01	-0.12	<0.001	-0.07	-0.10 to -0.03	0.01	-0.12	<0.001	-0.07	-0.11 to -0.03

*While removing the variance associated with the number of live births, hormone replacement therapy, hysterectomy, bilateral oophorectomy, body mass index, diastolic and systolic blood pressure, diabetes, education, income, and a composite lifestyle factor.
n.s. = not significant

Figure

Click here to access/download;Figure;Figure 1.png

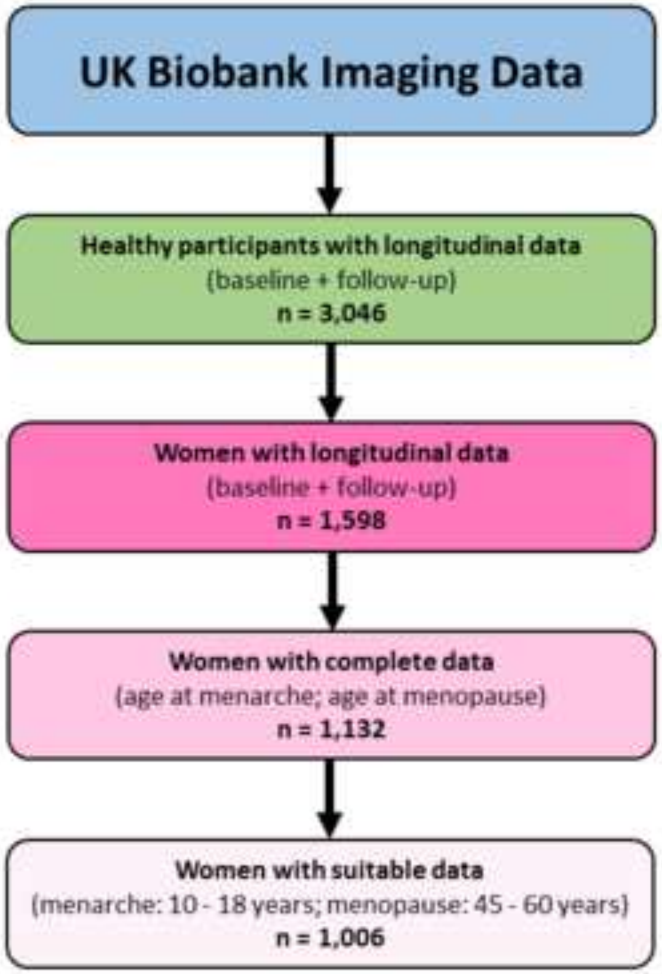

Figure

Click here to access/download;Figure;Figure 2.png

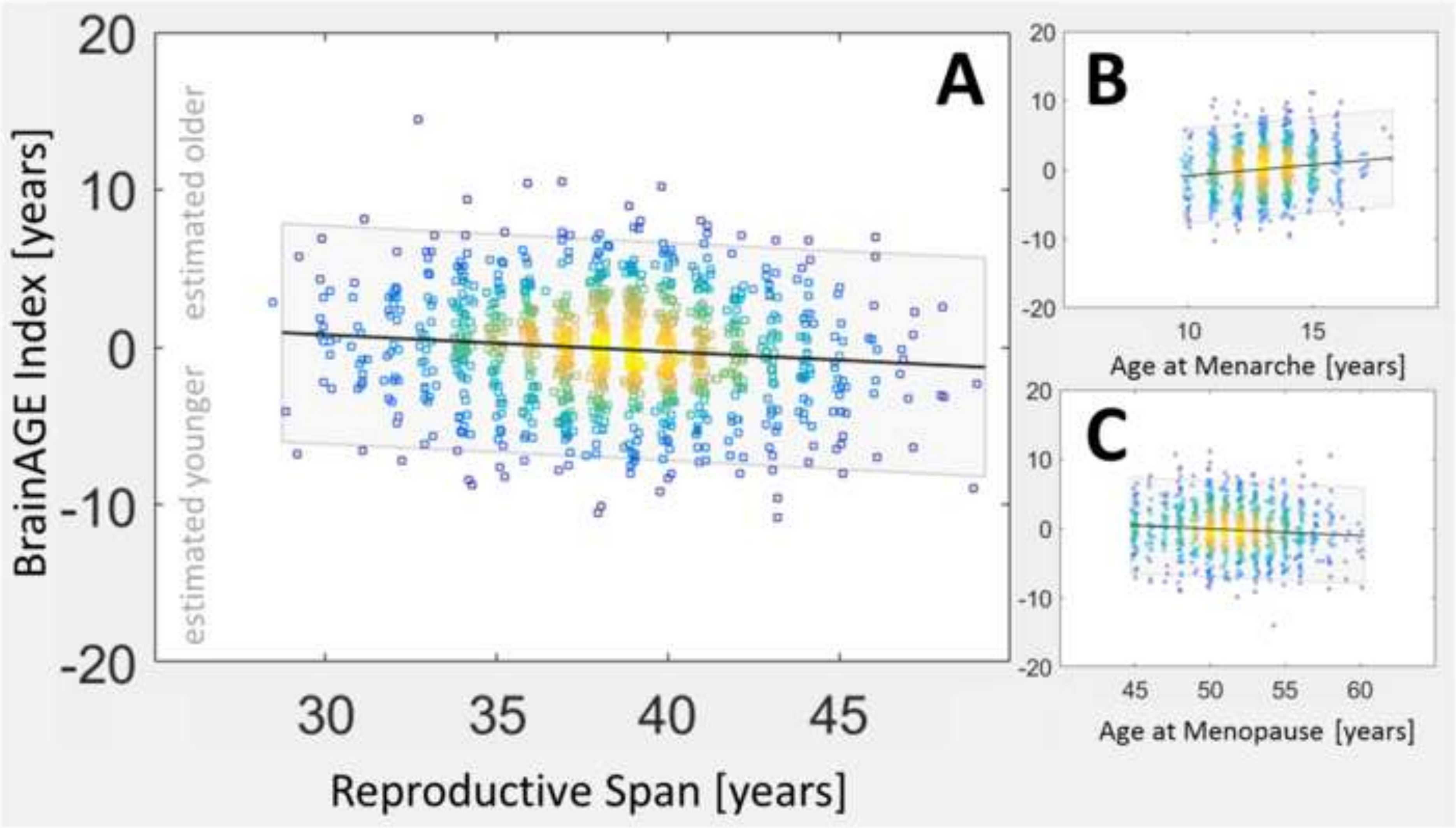

Click here to access/download;Figure;Figure 3.png

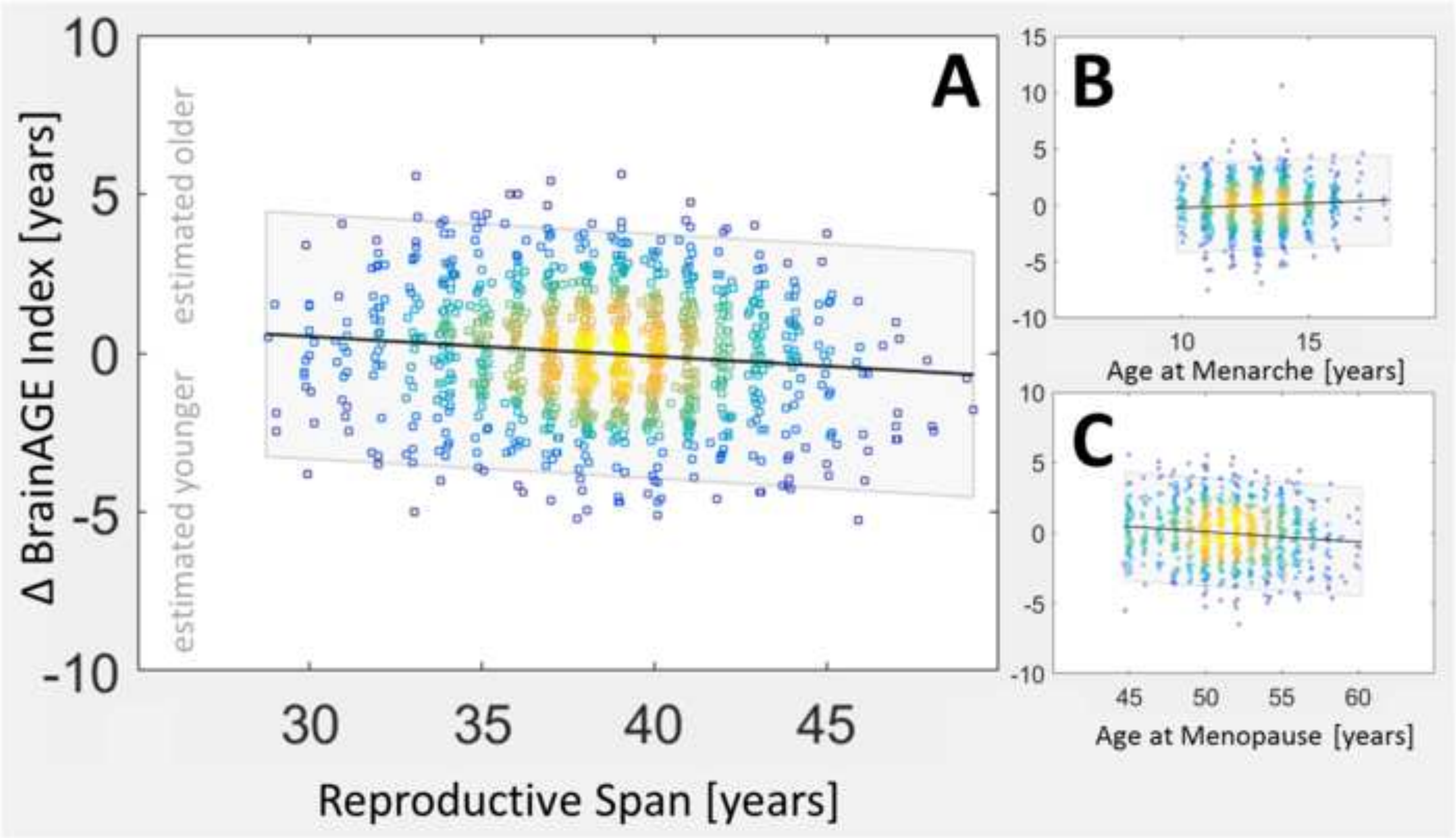

Click here to access/download
Supplementary Material
Supplemental Tables.docx

Cover Letter

Click here to access/download;Personal Cover;O_Cover Letter.docx

THE UNIVERSITY OF AUCKLAND
Te Whare Wānanga o Tāmaki Makaurau
NEW ZEALAND

SCIENCE

Building 302, Room 245
23 Symonds Street
Auckland 92019
New Zealand

The University of Auckland
Private Bag 92019
Auckland 1142
New Zealand
T +64 9 923 2928
E e.lueders@auckland.ac.nz

September 27th, 2024

Dear Editor(s),

Enclosed please find our manuscript, titled “***A Case for Estradiol: Younger Brains in Women with Earlier Menarche and Later Menopause***“, for your consideration as a publication in *GigaScience.*

In this large-scale study (n=1,006) we assessed links between brain age and milestones in a woman’s reproductive life, using both cross-sectional and longitudinal data. The findings of our study – younger brains in women with longer reproductive spans, earlier menarche, and later menopause – suggest that estradiol has brain-preserving effects.

Given that links between brain aging and significant milestones in a woman’s life are still severely understudied (even though this topic concerns half the population), we believe that the outcomes of our study would be of significant interest to a broad readership and an excellent fit for your journal.

Perhaps equally relevant, our findings in healthy post-menopausal women have important implications for clinical research because they highlight the powerful impact of estradiol and, as such, the potential of estrogen-replacement therapy to combat brain decline and preserve cognitive function later in life.

Please do not hesitate to contact us if you require further information.

Sincerely,

Eileen Lu(e)ders, PhD
[eileen.lueders@uppsala.se]
