# Supplementary material for: A Case for estradiol: younger brains in women with earlier menarche and later menopause
Source: Gigascience. 2025 May 23;14:giaf060. doi: 10.1093/gigascience/giaf060 (PMC12099614; doi:10.1093/gigascience/giaf060)
Supplement: giaf060_GIGA-D-24-00418_Revision_1 [file giaf060_giga-d-24-00418_revision_1.pdf]

GigaScience

A Case for Estradiol: Younger Brains in Women with Earlier Menarche and Later Menopause

--Manuscript Draft--

**Manuscript Number:**	GIGA-D-24-00418R1
**Full Title:**	A Case for Estradiol: Younger Brains in Women with Earlier Menarche and Later Menopause
**Article Type:**	Research
**Funding Information:**	Helse Sør-Øst RHF (2023037, 2022103) — Dr. Claudia Barth
**Abstract:**	The transition to menopause is marked by a gradual decrease of estradiol. At the same time, the risk of dementia increases around menopause and it stands to reason that estradiol (or the lack thereof) plays a role for the development of dementia and other age-related neuropathologies. Here we investigated if there is a link between brain aging and estradiol-associated events, such as menarche and menopause. For this purpose, we applied a well-validated machine learning approach in a sample of 1,006 postmenopausal women who were scanned twice approximately two years apart. We observed less brain aging in women with an earlier menarche, a later menopause, and a longer reproductive span (i.e., the time interval between menarche and menopause). These effects were evident both cross-sectionally and longitudinally, which supports the notion that estradiol contributes to brain preservation. However, further research is required because no direct measures of estradiol were obtained and effects were small overall.
**Corresponding Author:**	Eileen Luders<br>Uppsala Universitet<br>Uppsala, SWEDEN
**Corresponding Author Secondary Information:**	
**Corresponding Author's Institution:**	Uppsala Universitet
**Corresponding Author's Secondary Institution:**	
**First Author:**	Eileen Luders
**First Author Secondary Information:**	
**Order of Authors:**	Eileen Luders
	Inger Sundström Poromaa
	Claudia Barth
	Christian Gaser
**Order of Authors Secondary Information:**	
**Response to Reviewers:**	Please see our response letter. Thank you.
**Additional Information:**	
**Question**	**Response**
Are you submitting this manuscript to a special series or article collection?	No
**Experimental design and statistics**<br><br>Full details of the experimental design and	Yes

Powered by Editorial Manager® and ProduXion Manager® from Aries Systems Corporation

statistical methods used should be given in the Methods section, as detailed in our Minimum Standards Reporting Checklist. Information essential to interpreting the data presented should be made available in the figure legends.<br><br>Have you included all the information requested in your manuscript?	
**Resources**<br><br>A description of all resources used, including antibodies, cell lines, animals and software tools, with enough information to allow them to be uniquely identified, should be included in the Methods section. Authors are strongly encouraged to cite Research Resource Identifiers (RRIDs) for antibodies, model organisms and tools, where possible.<br><br>Have you included the information requested as detailed in our Minimum Standards Reporting Checklist?	Yes
**Availability of data and materials**<br><br>All datasets and code on which the conclusions of the paper rely must be either included in your submission or deposited in publicly available repositories (where available and ethically appropriate), referencing such data using a unique identifier in the references and in the "Availability of Data and Materials" section of your manuscript.<br><br>Have you have met the above requirement as detailed in our Minimum Standards Reporting Checklist?	Yes
GigaScience has policies and guidelines in place for the use of generative AI-writing tools such as ChatGPT. If you have used such writing tools to assist with writing the manuscript this must be	No

declared and cited in the text. Authors should not list AI-writing tools and other AI-assisted technologies as an author or co-author and should acknowledge that they are fully responsible for text generated or refined by AI-writing tools.<br><br>A summary of use (particularly in the introduction or among methods) needs to be included at the end of the paper, and the outputs should also be included as a supplementary file hosted in GigaDB or other open repositories. Please read our guidelines for more information. <br>By submitting to GigaScience, you are aware of the journal's AI-writing tools policy, and if you have declared use of such tools below, you have acknowledged this where appropriate in your manuscript and have made a summary of use and outputs available. <br><br>**AI-assisted writing tools have been used in the preparation of this manuscript?<br>** 	

Cover Letter Click here to access/download;Manuscript;R_Cover Letter.docx

THE UNIVERSITY OF AUCKLAND
Te Whare Wānanga o Tāmaki Makaurau
NEW ZEALAND
SCIENCE

The University of Auckland
Private Bag 92019
Auckland 1142
New Zealand
T +64 9 923 2928
E e.lueders@auckland.ac.nz

Building 302, Room 245
23 Symonds Street
Auckland 92019
New Zealand

April 8th, 2025

Dear Dr. Nicole Nogoy,

Thank you for your correspondence regarding our manuscript (GIGA-D-24-00418), titled "***A Case for Estradiol: Younger Brains in Women with Earlier Menarche and Later Menopause***", for your consideration as a publication in *GigaScience.*

We very much appreciate your feedback as well as the overall positive evaluation by both reviewers. We have thoroughly revised our manuscript according to the suggestions provided and we believe that the revised manuscript has been improved considerably.

For detailed point-by-point replies, please see our response letter.

Thank you very much for your continued interest in our study. Please let me know if you have any questions or require any additional information.

Warmly,

Eileen Luders, PhD
A/Professor, School of Psychology, University of Auckland, New Zealand

 Click here to access/download;Manuscript;R_Response to Reviews.docx

Response to Reviews

Reviewer #1: Overall Rating

This study explored the link between brain aging in women and estradiol-associated events, including menarche and menopause. Results show that estradiol may play a role in women's brain health. However, follow-up research is required because the effects in the current study were small. Specifically, estradiol was not directly explored as a central variable, which must be highlighted and validated in future studies. Nevertheless, the authors performed reliable research, and the data is open for access and may be replicable. This study should be accepted by GigaScience with major reviews. However, the authors still need to put effort into showing the main contributions and the literature gaps they fill with this piece of evidence and better explaining methodological procedures to support replicability. Besides, since the sample was conducted with most British women, caution should be taken regarding its generalization. Data collection was clearly conducted and curated, but the authors must explain further several points regarding methodological procedures, especially using the machine learning technique (e.g., hyperparameters, cross-validation, training and validation). I have conducted studies using technologies in healthcare systems and consultancy projects, but I lack expertise in the field of women's brain aging and reproductive areas. Thus, I strongly recommend another reviewer with expertise in this specific field to assess more nuances regarding brain aging. Below are my recommendations for improvements and points to be clarified.

Thank you very much for this positive assessment.

i) The abstract is well-written, but some improvements could be made. For example, the authors mention dementia and other related diseases in the context but do not emphasize brain aging; this seems disconnected from the study's objective. Also, if the research problem is unclear, consider pointing out research gaps in the literature or limitations of related work. This will help justify this research. In fact, the context should also be used to indicate previous studies using machine learning to identify similar relationships in order to justify the use of such techniques. Besides, more information should be provided about the methodological procedures used, especially those related to the machine learning technique used and the type of algorithm/framework (Gaussian Process Regression). The research strategy should move place (before presenting the results). The results should be more specific, e.g., "We observed less brain aging in women…" how much? The discussion should be rewritten to the importance of this study; currently, it does not sound positive. In this perspective, consider using "machine learning" in this study title, as it may help increase visibility.

(i) Please see our replies below regarding your comments on the Abstract.

ii) This study is very important, and I think it deserves a better introduction. For example, I found the introduction to be way too short and confusing at some points. Consider providing better context about the relationship between brain aging and estradiol-associated events, approaches used by previous studies and their key findings. Yes, the authors cited some works,

but superficially. Keep in mind that this will better support your research problem since I have found the problem to be addressed unclear. Besides, an entire paragraph should be developed to show how machine learning has been used for similar purposes and highlight the innovative approach used in this study. I also found the research problem obscure since it is unclear what specific gaps the study aims to fill. The BrainAGE should be better explained to show why it is an important metric.

(ii) Please see our replies below regarding your comments on the Introduction.

iii) Figures and Tables (also in sup. material) require some attention and enhancements. The criteria for including and excluding participants seem arbitrary and are not grounded on scientific findings. Figure 3 should be significantly improved as neither the text nor the Figure specifies each step of the sample workflow in depth. Table 3 also requires some attention, as the variable should be better explained regarding the possibility of bias. Great efforts should be made to improve the description of steps to deploy the machine learning algorithm (GPR), such as hyperparameter decision-making, overfitting techniques, data split between training and validation, etc. Check Boff Medeiros et al. as a good reference for this. Consider also checking Figure 1 as well as benchmarking to be used to describe the method. Single texts in subsections could be separated, e.g., PCA and GPR. The same should be applied to the "removed the linear age" - how did authors perform this? Consider explaining these procedures to support replication and transparency. PS: Sensitivity analysis also requires some improvements in Sup. Table 4.

(iii) Please see our replies below regarding your comments on the Methods.

iv) For the results, consider providing a context about BrainAGE values (e.g., what constitutes "young" and "old") and interpret the practical relevance of changes in BrainAGE or ΔBrainAGE. In addition to this, it would be important to better clarify the significance of slope values in tables, especially for those readers without statistical backgrounds. Additionally, it may be helpful to add sample size information and a quantitative scale for density in figures and tables while explaining the impact of adjustments for confounding variables on the results. In the discussion, the review calls for a deeper quantitative comparison with previous studies (this also has to be present in the introduction), distinguishing those directly related to estradiol and BrainAGE from those focused on broader cognitive outcomes. I recommend linking discussions to the presented results (finding X with Table Z or Figure Y). The conclusions should be split into three subsections showing contributions for practice, theory limitations and future works. In fact, limitations regarding ethnic diversity (e.g., predominance of British participants) should be highlighted and discussed, as well as how it may impact bias and choices for future works.

(iv) Please see our replies below regarding your comments on the Results and Discussion.

Abstract

i) The context clearly shows that the lack of estradiol may have played a significant role in causing/affecting mental illnesses like dementia. Still, I think that authors jump directly to the objective without highlighting the research problem or literature gap. For example, is this the first study investigating the link between brain aging and estradiol-associated events? Did previous studies try similar approaches using machine learning as a central technique? If so, what are the limitations that this study could support in advancing the field of research to fill out a literature gap? how does it differ from or build on previous studies? Consider providing more information for readers about it. In addition to this, I could not find anything related to brain aging since this will be the variable (central for this study) to be investigated with estradiol. Yes, the context mentions dementia and other related illnesses, but brain aging is very specific. Thus, consider making the context more focused on brain aging. ii) Besides, a "well-validated machine learning approach" is vague. Is this related to a specific machine learning technique, e.g., XGboost, SVM, etc? Or has a framework previously been validated in other similar contexts? Consider providing more information about the methodological procedures related to machine learning to enhance the method's credibility. iii) What kind of medical exams were used to assess brain aging? e.g., MRI and/or other exams? Besides, in the sentence "We observed less brain aging in women", how much less (%)? In addition, the notion of cross-section and longitudinal study should be described before the results, as this was your research strategy. iv) Consider excluding this sentence, "effects were small and no direct measures of estradiol were obtained in the current study," or explain better because it does not sound good, which almost invalidates this research as it sounds abrupt and may give the impression that the study is insignificant. Instead, highlight how these small observed effects and the absence of direct measurements of estradiol contribute to the literature, e.g., fill research gaps and open avenues for future research such as...???

(i-iv) We sincerely thank the reviewer for their thoughtful and constructive feedback on the abstract. While we appreciate the detailed suggestions, we propose keeping the changes balanced to maintain focus and clarity. Nevertheless, we have extended the abstract and made several adjustments to address comments i-iv. Further details on the rationale, methods, results, and discussion are provided in the respective sections of the article. The updated abstract is provided below.

> ***Abstract***
> *The transition to menopause is marked by a gradual decrease of estradiol. Concurrently, the risk of dementia in women increases around menopause, suggesting that estradiol (or the lack thereof) plays a role in the development of dementia and other age-related neuropathologies. Here, we set out to investigate whether there is a link between brain aging and estradiol-associated events, such as menarche and menopause. For this purpose, we applied a well-validated machine learning approach to analyze both cross-sectional and longitudinal data from a sample of 1,006 postmenopausal women who underwent structural magnetic resonance imaging (MRI) twice, approximately two years apart. We observed less brain aging in women with an earlier menarche, a later menopause, and a longer reproductive span (i.e., the time interval between menarche and menopause). These effects were evident both cross-sectionally and longitudinally, supporting the notion that*

estradiol has neuroprotective properties and contributes to brain preservation. However, further research is required because the observed effects were small, estradiol was not directly measured, and other factors may modulate female brain health. Future studies might benefit from incorporating actual estradiol (and other hormone) measures, as well as considering genetic predispositions and lifestyle factors alongside indicators of brain aging to deepen our understanding of estradiol's role in maintaining brain health. Additionally, including more diverse study populations (e.g., varying in ethnicity, socioeconomic status, and health status) in follow-up research would enhance the generalizability and applicability of these findings.

v) The title could be improved to "using machine learning to explore the impact of estradiol on brain aging: insights from menarche to menopause". Keep in mind that the use of "machine learning" will definitely increase visibility.

(v) Thank you for the suggestion. We appreciate your input but prefer to retain the current title as it is more engaging and likely to attract a broader audience. However, we are open to considering a more technical title if the Editor feels it would be more appropriate for GigaScience.

Introduction

i) Consider expanding results of postmenopause studies (e.g., 3-6) that show dementia in women is known to increase around menopause. For example, cite the authors et al., their approaches and key findings. The way it is presented gives too much effort for readers to look into these papers and check the main results. ii) Before presenting the research problem, an entire paragraph should show more context about these studies above and 4,7-14,18-20. In this regard, expand the problems related to dementia and what are the conclusions of previous research so far. In addition, it provides the main approaches used to understand the link between estradiol and dementia in women.

(i-ii) Thank you for these suggestions. We added more studies (also in reply to Reviewer #2) to the Introduction alluding to the potential of estradiol and its role in maintaining brain health and protecting against age-related degeneration (see below). While summarizing the approaches and key findings for each study would be excessive, focusing solely on dementia-related studies would seem arbitrary, especially since dementia is not the central focus of this study. Therefore, we propose keeping the Introduction as concise as possible to maintain the overall focus and readability of the manuscript. Readers interested in the details of any of the cited studies (including those related to dementia) can refer to the original references, which provide comprehensive insights into the respective approaches and findings. This approach reflects our aim to balance brevity with informativeness while staying within the constraints of the manuscript.

Introduction

[…] The risk for dementia in women is known to increase around menopause[3-6] and thus it stands to reason that estradiol plays a role for the development of dementia and other age-

related neuropathologies. Indeed, studies using animal models have demonstrated that estradiol promotes synaptic plasticity, enhances neurogenesis, and protects against oxidative stress and neuroinflammation[7-13]—mechanisms that are critical for maintaining brain health and mitigating age-related brain degeneration. While extensive research has also been conducted in humans, focusing on specific phases (e.g., menarche, pregnancy, menopause) or interventions (e.g., hormonal contraceptives, menopausal estrogen therapy and anti-estrogen therapy) definitive evidence for the neuroprotective role of estradiol remains elusive[4,14-29]. Specifically, in the context of menarche and menopause, both early and late onset have been associated with an increased risk of dementia as well as with markers of brain aging and cognitive function[4,14-21,25-27]. […]

iii) Regarding the research problem, it is unclear what specific gaps the study aims to fill. For example, the authors do not explicitly state whether previous studies have failed to use longitudinal approaches or whether there is controversy about the relationship between estradiol and brain health. I wonder, if there is no controversy, what is the need for this study?

(iii) The Introduction outlines the controversy about the relationship between estradiol and brain health as shown above. Apologies if the comment on the existing UK Biobank studies was confusing (we removed it now). Note, longitudinal studies exist, but the outcomes are not conclusive. Below is the updated paragraph explaining the goal of our study:

Introduction

[…] To further advance this field of research, the current study set out to determine if there is a link between a woman's estimated brain age (a biological marker of brain health[30]) and the reproductive span (i.e., the interval between menarche and menopause when estradiol levels are high). If a lack of estradiol is among the driving factors for diminished brain health later in life, brain age and reproductive span should be inversely related (negative correlation). To be able to relate our findings to others in the literature[14-16] and to provide a frame of reference for future studies, we additionally investigated if there is a significant link between estimated brain age and the age at menarche as well as the age at menopause. Assuming a neuroprotective effect of estradiol, we expected that a lower brain age would be linked to an earlier menarche (positive correlation) and to a later menopause (negative correlation). Importantly, our study comprises both cross-sectional and longitudinal components, with follow-up data acquired approximately two years after the initial brain scan.

iv) The BrainAGE algorithm should be better explained to show readers what it means or why it is an important metric, especially for those unfamiliar with the concept, as this can lead to confusion.

(iv). The BrainAGE algorithm is explained as follows in the Introduction (see below). Additional details are now provided in the Methods section (please see our replies on that further below).

Introduction

[…] To estimate brain age, we used structural brain images and a well-validated high-dimensional pattern recognition approach, as detailed elsewhere [31,32]. Briefly, the difference between the estimated brain age and the chronological age yields a so-called brain age gap estimate (BrainAGE) in years. The BrainAGE index is negative if a brain is estimated younger than its chronological age; it is positive if a brain is estimated older than its chronological age. For example, a 50-year-old woman with a BrainAGE index of -3 years shows the aging pattern of a 47-year-old. The BrainAGE algorithm has been shown to be robust and reliable across datasets, age-ranges, and scanner types[31,33]; it has been successfully applied in a wide range of studies[31,32,34-36] including those capturing hormonal changes in women[37,38]. Moreover, the BrainAGE index has been demonstrated to work as a predictor of dementia as well as age-related cognitive decline[34,39]. A major advantage of the BrainAGE approach is its ability to aggregate complex, spatially distributed age-related changes in brain structure into a single, interpretable biomarker. Such brain age metrics provide a powerful way to study the influence of biological factors across the female lifespan, including the effects of cumulative estrogen exposure and genetic risk for age-related brain degeneration, as also shown in other studies. [14]

v) Although I have no experience in this area, I still found the argumentative structure somewhat non-linear. For example, the introduction switches between different topics (risk of dementia, menarche, menopause, estradiol, brain age) without building a fluid narrative that connects all the elements cohesively.

(v) Thank you for your feedback regarding the structuring. We understand that there are individual preferences. The current format follows our thinking and we believe the structure chosen effectively conveys the various elements needed to provide context for our study. This sentiment was echoed by Reviewer #2, who stated:

"The introduction is largely well written and promptly describes the bigger picture about links between neuroprotective effects of estradiol and potential importance in brain health and aging. The introduction also does a good job to spell out what BrainAGE is and how one could interpret it. By the end of the introduction the reader comes away with a clear understanding of the directional predictions that the authors make."

That being said though, we made numerous adjustments to the Introduction as per your other suggestions (i-iv), as detailed above.

vi) The authors mention the machine learning technique to estimate brain age, which, in my opinion, is an important innovative factor of this research. However, it is mentioned superficially. A full paragraph should be developed explaining why this approach is relevant and what related work has been developed using similar techniques. This is crucial to strengthen the justification for this study. For example, DE LANGE, Ann-Marie et al. Cumulative estrogen exposure, APOE genotype, and women's brain aging-a population-based neuroimaging study. bioRxiv, p. 826123, 2019.

(vi). Thank you very much. And yes, we devoted a full paragraph in the Introduction to summarizing the BrainAGE algorithm, including the suggested reference to De Lange et al., 2019. Please see the text excerpt above (in reply to your comment iv). Additional details are now offered in the Methods section. Please see our replies on that further below (Methods comments v-vi).

Briefly, the core of our BrainAGE framework uses Gaussian Process Regression (GPR), a non-parametric Bayesian approach well suited for modelling complex relationships in neuroimaging data. We revised our original framework (Franke et al. 2010), which used Relevance Vector Regression (RVR), and adopted GPR due to its improved stability and performance, especially when dealing with the large datasets common in modern neuroimaging studies, as demonstrated in our previous work (Kalc et al. 2024). GPR provides a flexible framework for kernel selection, allowing us to effectively model the relationship between brain features and age. Studies comparing different algorithms for estimating brain age have shown GPR to be a strong performer, often comparable to deep learning methods, but with advantages in interpretability and handling of small to medium-sized datasets (Kalc et al. 2024).

A key innovative aspect of our current study is the application of a GPR-based ensemble approach. Specifically, we used a stacking methodology in which predictions from multiple GPR base models trained on different brain tissue segmentations (grey and white matter) and different preprocessing parameters (e.g., smoothing levels) were combined using a general linear model where the weights of the models were derived by maximizing the variance to the parameter of interest. This stacking ensemble approach serves as an effective regularisation technique, reducing the risk of overfitting and exploiting the complementary information from different feature sets.

While other machine learning techniques are used to estimate brain age, such as the XGBoost approach used by De Lange et al. (2019) to investigate the effects of estrogen exposure, our GPR stacking method represents a distinct approach. De Lange et al. used XGBoost based on cortical and subcortical volume/thickness measurements. In contrast, our method relies on the probabilistic framework of GPR and specifically exploits ensemble learning by stacking GPR models trained on voxel-based morphometric features. While both studies use the brain age gap concept derived from MRI, the underlying algorithms, feature inputs and model building strategies differ, highlighting the ongoing evolution and diversification of methods in the field. Our approach contributes a robust, high-performance GPR-based alternative, optimised by ensemble techniques.

Method

i) Supplemental Table 3 presents the ethnic background of women with longitudinal MRI data used in this study. Consider including percentages to show the proportion of each group in relation to the total. I wonder if the authors should report factors that could impact the study, such as age, socioeconomic status, or previous health conditions, which could vary between ethnic groups and affect the results. Also, "British" seems very generic. Could different parts of the UK show nuances that should be considered?

(i) We assume your review refers to an older version of our manuscript as there was no Supplemental Table 3 in the draft submitted to GigaScience. However, we re-added the table on the ethnicities and also included the percentages, as requested. The Methods section has been adjusted accordingly. Moreover, the Abstract and Discussion have been extended to comment on the generalizability of the findings. Note, the information of "British" has been obtained as such from the UK Biobank; there are no further nuances.

Abstract
[…] Additionally, including more diverse study populations (e.g., varying in ethnicity, socioeconomic status, and health status) in follow-up research would enhance the generalizability and applicability of these findings.

Methods
[…] The UK Biobank is a biomedical database and research resource that contains genetic, lifestyle and health information from half a million people. In the UK Biobank cohort, 94.6% of participants are of white ethnicity[40]. For general ethnic information, see https://biobank.ctsu.ox.ac.uk/crystal/field.cgi?id=21000; for ethnic information on all women with available longitudinal data, see ***Supplemental Table 3****.*

Supplemental Table 3. *Ethnic background of women with longitudinal MRI data (n=1,598)*

Ethnicity	***Number of Women***
British	*1497 (93.68%)*
Any other white background	*42 (2.63%)*
Irish	*25 (1.56%)*
Chinese	*9 (0.56%)*
Other ethnic group	*5 (0.31%)*
Caribbean	*5 (0.31%)*
Indian	*3 (19%)*
Pakistani	*3 (19%)*
African	*3 (19%)*
Any other mixed background	*3 (19%)*
White and Black Caribbean	*2 (13%)*
Any other Asian background	*1 (0.06%)*
White and Black African	*0 (0%)*

Information on ethnicity was collected based on self-reports using a touchscreen questionnaire at the UK Biobank Assessment Centre.

Discussion
[…] Lastly, the UK Biobank (i.e., the source of the current sample) is biased towards healthy and more socioeconomically privileged individuals with a predominant white ethnic background[40]. Thus, conducting research in more diverse populations, including individuals from different ethnic, socioeconomic, and health backgrounds, would improve the generalizability of findings and provide a broader understanding of the relationship between estradiol and brain health.

ii) I got curious about the inclusion and exclusion of participants - is there a reference to justify age at menarche was younger than ten or older than 18, or whose age at menopause was

younger than 45 or older than 60? If so, consider providing a source or a brief explanation to support replication. Keep in mind that these ranges may seem arbitrary without a clear justification.

(ii) We did not follow a specific reference when including / excluding women according to their age at menarche and menopause. The cutoffs were chosen based on the data available. In other words, most women ranged between the chosen cutoffs and the few outliers were excluded to increase the homogeneity of the group.

iii) Figure 3 shows the workflow of sample collection. Consider providing the criteria for inclusion and exclusion used, such as previous diagnosis, medication use, or other conditions during each stage. Besides, separate into stages 1, 2, etc. In addition, instead of just presenting absolute numbers (n), excluding percentages between each stage would give a clearer idea of the proportion of participants removed at each stage, e.g., "36% of women with longitudinal data were excluded for missing X and Y reasons.". This information could be presented on the right side of each step, and new arrows between existing ones ->.

(iii) We assume the reviewer refers to Figure 1, providing a flowchart of the sample selection. If yes, we prefer the current layout to simplify things and make it easier on the reader. Additional information (e.g., on the inclusion / exclusion criteria) is provided in the Methods section.

iv) Table 3 presents the descriptive characteristics of the sample of women included in the study, detailing variables related to age, among others. As a recommendation for improvement, consider including the percentage of women who met each inclusion criterion, e.g., how many live births, hysterectomies or oophorectomies were distributed among the final participants (n=1,006)? This could help to check for important biases. In addition, the authors could explain better why these variables are considered for analysis for a broader audience. For example, I am interested in the results of this study because of the use of machine learning techniques. Still, I lack expertise in brain aging and estradiol, so it is hard to understand some issues. For example, In Table 3, the variable related to the number of women with hormone replacement therapy (yes: 306 | no: 700) makes me wonder if this difference could impact the results somehow, i.e., HRT could affect brain aging and introduce variation in outcomes. If not, make this explicit after calling Table 3 in the text, showing the importance of each variable for the study. The same should be applied to other variables like the number of women with hysterectomies (yes: 48 | no: 958), etc.

(iv) We assume the reviewer refers to Table 1. We added the percentages, as requested.

Table 1. *Sample characteristics*

Variable	*Descriptive Statistics*
Age at the initial brain scan	*mean ± SD: 63.20 ± 6.42 years*
Age at the follow-up brain scan	*mean ± SD: 65.54 ± 6.37 years*
Age at menarche	*mean ± SD: 13.02 ± 1.53 years*
Age at menopause	*mean ± SD: 51.41 ± 3.23 years*
Reproductive span	*mean ± SD: 38.39 ± 3.55 years*

Number of live births	*mean ± SD: 1.75 ± 1.16*
Number of women with hormone replacement therapy	*yes: 306 (30.42%) | no: 700 (69.58%)*
Number of women with hysterectomy	*yes: 48 (4.77%) | no: 958 (95.23%)*
Number of women with bilateral oophorectomy	*yes: 37 (3.68%)| no: 969 (96.32%)*

SD = standard deviation

v) The authors mention the application of PCA for data reduction but lack detail on how the principal components were selected. For example, there is no mention or description of how the data were normalized before PCA, which is essential for the components to be comparable.

(v) Thank you for pointing this out. We aimed at keeping the description concise, but here is additional information on the PCA: Prior to applying Principal Component Analysis (PCA) for dimensionality reduction of the voxel-based morphometry maps, the feature data within the training set were normalized by scaling the values between 0 and 1 and subtracting the mean. The same scaling and mean subtraction were then applied to standardize the test set data. PCA was then performed on the normalised training data. The number of principal components to be retained was determined based on the theoretical maximum rank of the centred data matrix. This approach was chosen because the maximum rank of the centred data matrix (and thus the maximum number of principal components with non-zero variance) is determined by the smaller of the two dimensions (number of subjects N or number of voxel p) minus one. By choosing min(N, p) - 1 components, we ensure that we capture the entire variance structure represented in the data, regardless of whether the number of subjects exceeds the number of features or vice versa, while excluding mathematically redundant dimensions. The transformation matrix derived from the training data PCA was used to project the standardized test data onto this selected k-dimensional principal component space. The Methods description has been extended as follows:

> ***Data Analysis***
> *[…] For further data reduction, we applied a principal component analysis (PCA) using singular value decomposition to all the models using n–1 PCA components (n = minimum of voxel number or sample size). Prior to applying the PCA, the data were normalized by scaling the values between 0 and 1 and subtracting the mean. The transformation matrix derived from the training data PCA was used to project the normalized test data onto this principal component space.*

vi) Regarding the machine learning (Gaussian Process Regression) applied in this study, please explain the reason for specifically selecting this model and not others. Furthermore, were the hyperparameters (e.g., 100 for the constant mean function and -1 for the likelihood function) set? Also, should the procedures regarding the 10-fold approach be expanded, e.g., how the data was split between training and validation? Were any techniques applied to avoid overfitting, such as cross-validation, etc? Besides, is there any chance of sharing the dataset on GitHub to assess and allow replication? How was data split into data training and validation? Check the methodological procedures of the following paper as a reference for good practice regarding these details: BOFF MEDEIROS, Natália et al. Predicting the length-of-stay of pediatric patients using machine learning algorithms. International Journal of Production Research, p. 1-14, 2023.

(vi) We are happy to provide further explanations:

- Model Selection Rationale: GPR was selected as the core machine learning algorithm for its strong theoretical foundation in Bayesian inference and demonstrated stability and accuracy in previous brain age prediction studies, including our own foundational work (Kalc et al., 2024). Its flexibility via kernel functions allows capturing complex non-linear relationships between brain features and age.

- Hyperparameters: The hyperparameters (mean function and likelihood noise variance) and the chosen kernel were set to specific values based on prior exploratory analysis in our method paper (Kalc et al. 2024). The log-likelihood optimizer was used to estimate the optimal parameters from the training data and our global BrainAGE model was validated using different external data set: i) a synthetic dataset of global neocortical thinning; ii) ADNI sample; iii) schizophrenia dataset.

- Cross-Validation and Overfitting Prevention: To avoid overfitting and ensure generalizability, we employed 10-fold cross-validation, where the dataset was randomly partitioned into 10 equally sized subsets. In each iteration, one fold was used as the test set, and the remaining nine as the training set. This process was repeated 10 times, and performance metrics (e.g., MAE) were averaged across folds.

The Methods description has been extended as shown below:

> ***Data Analysis***
> *For the estimation of the BrainAGE index, we employed a Gaussian Process Regression (GPR)[43] that uses a linear covariance function, a constant mean function, and a Gaussian likelihood function. Hyperparameters were set to 100 for the constant mean function and to -1 for the likelihood function based on prior exploratory analyses[33]. As training data, we selected 3,046 individuals from the UK Biobank where two time points were available. To avoid overfitting and ensure generalizability, we employed 10-fold cross-validation separately for the initial and follow-up brain scan, where the dataset was randomly partitioned into 10 equally sized subsets. In each iteration, one fold was used as the test set, and the remaining nine as the training set. This process was repeated 10 times, and performance metrics (e.g., MAE) were averaged across folds. To estimate the individual brain ages, eight models based on the aforementioned sets of images (i.e., gray matter/white matter, 4 mm/8 mm Gaussian kernel, and 4 mm/8 mm image resolution) were combined using a general linear model where the weights of the models were derived by maximizing the variance to the parameter of interest (e.g., menopause). The difference between the resulting estimated brain age and the chronological age was then calculated as the BrainAGE index (in years).*

Last but not least, this is to confirm that we have reviewed the methodological standards outlined by Boff Medeiros et al. (2023). We ensure that our revised manuscript aligns with best practices in machine learning transparency and reproducibility. In terms of data availability, due to privacy

restrictions and consent limitations, the raw MRI data cannot be publicly shared. However, we have made all code and preprocessing pipelines available in a GitHub repository (https://github.com/ChristianGaser/BrainAGE, https://github.com/ChristianGaser/BrainAGE-UKBiobank).

vii) Supplemental Table 4 addresses the adjustment for a factor called "GLS", which was used to adjust the results in the sensitivity analyses. This table could be improved in some ways. For example, the authors did not justify why the 16 specific variables were chosen to represent the participants' lifestyles - selection criteria based on literature or statistical relevance? Furthermore, I wonder how the data were adjusted, given that the variables have different units of measurement. Also, consider showing how each variable weighs on the participants' lifestyles, e.g., physical activity may be more impactful than watching TV.

(vii) We assume the reviewer refers to Supplemental Table 1 summarizing the components for the General Lifestyle Score. If yes, please know that this score was developed and provided as such by the UK Biobank. We had nothing to do with the selection criteria and compilation.

Results

i) In Figure 1, the authors could improve the interpretation of the magnitude of the Y-axis scale (BrainAGE index) by indicating the ranges of practical relevance. For example, I wonder how much represents a "young" value (-10 or -5)? Likewise, how much represents an "old" value (+5 or +10)? It would also be important to add information about the sample size in each ratio (number of points used in each panel - A, B and C). Perhaps show examples of how these values translate to brain health or aging, e.g., does -10 years mean X% reduction in some risk? ii) Furthermore, there is no quantitative scale to show the meaning of the colors (e.g., points per area on graphs), although the legend indicates that the colors represent density. Consider including some of this information (e.g., n. of samples) in Table 1. iii) Not much to add to Figure 2 and Table 2, but the same improvements that I have provided in Figure 1 and Table 1. Still, as I mentioned in Figure 1, the authors can explain what a change of -5 or +5 years in the Δ BrainAGE Index represents. I wonder if there is some cutoff or threshold (e.g., +10 or -10 years) that has clinical or scientific relevance... Without this practical interpretation, the values on the Y-axis are just abstract numbers. Remember that a reader may be uncertain about the magnitude of the effects, e.g., is -5 years in BrainAGE meaningful for brain health? Are values like +1 year or -1 year considered important, or are they normal margins of error of the model (similar to my question about the BrainAGE index)?

(i-iii) We assume the reviewer is referring to Figure 2 and Figure 3, which present the findings. The numbers on the y-axis represent the BrainAGE index in years (Figure 2) or the change in the BrainAGE index in years (Figure 3), respectively. Negative numbers mean that brains are estimated younger than they are; positive numbers mean that brains are estimated older than they are. The actual number indicates the magnitude (e.g., a BrainAGE of +5 years indicates that the brain appears to be 5 years older than its chronological age). This is explained in the Introduction (as shown below). The sample size was always the same (n=1,006) as we analyzed

the same women in the cross-sectional and longitudinal analyses when correlating BrainAGE (change in BrainAGE, respectively) with (A) reproductive span, (B) Age at Menarche, and (C) Age at Menopause. In other words, the squares in the density plot refer to the individual participants in each analysis and there are always 1,006 participants in panel A, B, C. Of course, not all 1,006 can be visually discriminated as the overlap / density was high at times, so we used the density color scheme (hot color = large overlay; cool color = little overlay). The figure legends have been updated to convey this information, including the sample size of 1,006 (as shown below). With respect to your other question: Currently, there are no established guidelines or cut-offs for the BrainAGE index in terms of clinical relevance. However, to facilitate the interpretation of the plots, we provided some extra information on the slopes in the Results section (see our reply to your next comment).

> ***Introduction***
>
> *[…] Briefly, the difference between the estimated brain age and the chronological age yields a so-called brain age gap estimate (BrainAGE) in years. The BrainAGE index is negative if a brain is estimated younger than its chronological age; it is positive if a brain is estimated older than its chronological age. For example, a 50-year-old woman with a BrainAGE index of -3 years shows the aging pattern of a 47-year-old. The BrainAGE algorithm has been shown to be robust and reliable across datasets, age-ranges, and scanner types[31,33;] it has been successfully applied in a wide range of studies[31,32,34-36] including those capturing hormonal changes in women[37,38]. Moreover, the BrainAGE index has been demonstrated to work as a predictor of dementia as well as age-related cognitive decline[34,39.]*
>
> ***Figure Legends***
>
> *Figure 2. Correlations with BrainAGE at the initial brain scan. The x-axes show the reproductive span (age, respectively) in years. Of note, age in the UK Biobank has been rounded to the year, so we added a small random jitter to the x-axes to give a better overview about the age distribution. The y-axes show the BrainAGE index in years, with negative values indicating that brains are estimated younger than their chronological age and positive values indicating that brains are estimated older than their chronological age. Panel A displays a negative link between the BrainAGE index and the reproductive span (the longer the reproductive span, the younger the estimated brain age). Panel B displays a positive link between the BrainAGE index and the age at menarche (the earlier the onset of menarche, the younger the estimated brain age). Panel C displays a negative link between the BrainAGE index and the age at menopause (the later the onset of menopause, the younger the estimated brain age). The squares in the density plot represent the individual measures (n=1,006); hot colors indicate a larger overlay of measures; cool colors indicate a smaller overlay. The shaded band is the 95% confidence interval.*
>
> *Figure 3. Correlations with BrainAGE over 2.35 years (Δ BrainAGE). Panel A displays a negative link between the BrainAGE index and reproductive span (the longer the reproductive span, the smaller the estimated brain age). Panel B displays a positive link between the BrainAGE index and the age at menarche (the earlier the onset of menarche, the younger the estimated brain age). Panel C displays a negative link between the*

BrainAGE index and the age at menopause (the later the onset of menopause, the younger the estimated brain age). The squares in the density plot represent the individual measures (n=1,006); hot colors indicate a larger overlay of measures; cool colors indicate a smaller overlay. The shaded band is the 95% confidence interval.

iv) In Supplemental Table 1 and Sup. In Table 2, the authors could briefly explain the slope values in practical terms. For example, what does a change of -0.11 or -0.06 in BrainAGE mean for each additional year in the reproductive period? Furthermore, for analyses that were adjusted for multiple variables (e.g., BMI, blood pressure), the authors could make clear how these adjustments impacted the results - did the coefficients change significantly after adjustment?

(iv) Thank you for the comment. Of note, the slopes as well as the outcomes of the sensitivity analyses are explained in the Results section:

Results

[…] The slopes of the regression indicate different rates of change for menarche and menopause (0.32 and -0.10, respectively). More specifically, for each year younger at menarche, brains are estimated 0.32 years younger (which corresponds to 3.2 years younger for each 10 years). In contrast, for each year older at menopause, brains are estimated 0.1 year younger (which corresponds to 1 year younger for each 10 years).

[…] The slopes of the regression are still somewhat different for menarche and menopause (0.08 and -0.06, respectively), albeit more similar than in the cross-sectional analysis: For each year younger at menarche, brains are estimated 0.08 years younger (0.8 years over 10 years), whereas for each year older at menopause, brains are estimated 0.06 years younger (0.6 years over 10 years).

[…] *The results described above remained comparable when removing the variance associated with the number of live births, hormone replacement therapy, hysterectomy, bilateral oophorectomy, body mass index, diastolic and systolic blood pressure, diabetes, education, income, and a composite lifestyle factor. In other words, when examining the association between BrainAGE and reproductive span, we observed a negative association. Likewise, there was a positive association between BrainAGE and age at menarche and a negative association between BrainAGE and age at menopause. The effects were significant for reproductive span, menarche, and menopause for the cross-sectional analyses (see Table 2; Sensitivity Analyses), and for reproductive span and menopause for the longitudinal (see Table 3; Sensitivity Analyses).*

Discussion

i) The authors mention related work such as 8,10,13,14, etc, showing similar findings. However, there is no quantitative data demonstrating alignment with studies, e.g., how similar are the

results of this study to previous findings? e.g., correlations, effect sizes, etc. Consider providing a new table showing approaches, key findings, and quantitative differences and similarities between this study and the existing literature. The lack of an in-depth comparison makes understanding how this study fills a literature gap/limitations difficult.

(i) Thank you for your thoughtful suggestion. While we appreciate the appeal of a detailed comparison with prior studies, including a new table summarizing approaches, key findings, and quantitative differences or similarities goes beyond the intended scope of our research. Our study focuses on presenting novel insights rather than conducting an extensive review or meta-analysis, which would be more appropriate for a dedicated review article. Nevertheless, we have ensured that our discussion contextualizes our findings within the existing literature and highlights their unique contributions.

ii) Furthermore, these studies should be clear about which ones directly support the association between estradiol and BrainAGE and which of these studies studied dementia or cognition without considering the BrainAGE index and used machine learning as a central technique. In fact, this should also be explicit in the introduction of this study, as I mentioned above.

(ii) Please see our replies to your comments regarding the Introduction above.

iii) Authors should put effort into linking the discussions with their results. For example, "both an earlier menarche and a later menopause were significantly associated with less brain aging, according shown in Figure X, or Table Y"

(iii) As recommended, we extended the Discussion as follows:

> ***Discussion***
> *[…] We detected less brain aging in women with longer reproductive spans, earlier menarche, and later menopause (see Figures 2-3 and Tables 2-3).*

iv) Supplemental Table 3 highlights a limitation of the study: ethnic representation. The predominance of British women limits the applicability of the results to more diverse populations, which may be relevant in studies of brain health and reproductive factors. Consider further developing these study limitations, showing how future work could be developed in this perspective. Furthermore, the conclusion could be divided into three subsections: a) Contributions for practice (how this study can support clinicians in real-life healthcare settings, e.g., how might these results influence the use of hormone replacement therapy? OR are there any recommendations for monitoring reproductive or brain health?, b) Contributions for theory (how this study fills the literature gaps - this requires more clarification) and c) Limitations and future works.

(iv) We revised the Conclusion (also in reply to reviewer #2) as shown below. In addition, we comment on limitations in the preceding text within the Discussion:

Discussion

[…] The current findings seem to suggest a protective effect of estradiol and as such seem promising in the framework of prevention and intervention. However, further research is required, as the effect sizes for the observed associations were small (albeit smaller effect sizes are not uncommon in studies with larger sample sizes [72]) and various factors, such as genetics, life style, or hormones other than estradiol, could play a greater (or at least an additional) role in preserving brain health[2,73,74]. Moreover, our study did not measure estradiol directly, and links between estradiol and brain aging seem to be rather complex as indicated by the outcomes of other studies. […]

Conclusion

Our study revealed less brain aging in women with a larger reproductive span, earlier menarche, and later menopause. Thus, sex hormones—potentially estradiol—may contribute to brain health. However, follow-up research is required because the effects observed in the current study were small, estradiol was not directly examined, and female brain health is likely be modulated by factors other than estradiol. Future studies might benefit from incorporating actual estradiol (and other hormone) measurements, as well as considering genetic predispositions and lifestyle factors alongside structural brain measures. Moreover, to build a more comprehensive understanding and expand this understudied field, future research focusing on specific timeframes surrounding menopause—such as perimenopause (i.e., the time preceding the final menstrual period) or early postmenopause (e.g., the initial year after menopause) versus late menopause (e.g., ten years after menopause)—would be valuable. Lastly, the UK Biobank (i.e., the source of the current sample) is biased towards healthy and more socioeconomically privileged individuals with a predominant white ethnic background[74]. Thus, conducting research in more diverse populations, including individuals from different ethnic, socioeconomic, and health backgrounds, would improve the generalizability of findings and provide a broader understanding of the relationship between estradiol and brain health.

Reviewer #2:

Overall Assessment:

In this work Luders et al., examines MRI scans of n=1006 women from the UK Biobank database. The gray and white matter structural MRI features were measured from the T1-weighted MRI images and then fed into a machine learning model that makes robust and accurate predictions about age based on these brain features. That machine learning model, called BrainAGE, outputs a number that can be interpreted relative to a person's chronological age – lower BrainAGE scores indicate an individual with brain features that are younger than the typical person of the same chronological age. The rationale behind the study was to use BrainAGE output as an indicator of brain health and aging and test whether variables such as age at menarche, menopause, and reproductive span (i.e. span between menarche and

menopause) would predict BrainAGE. The basis for predicting that such variables might be predictive of BrainAGE stems back to literature showing that estradiol tends to be neuroprotective and because risk of dementia tends to increase around menopause. Since menarche and menopause are life events that are associated with changes in estradiol levels, they are used as proxy measures for the main biological mechanisms of interest – estradiol. Overall, the authors show a small but significant relationship in the predicted directions for age at menarche, age at menopause, reproductive span for predicting BrainAGE estimates. These results are found in cross-sectional data (e.g., initial brain scans of the n=1006 women) and then found again in delta BrainAGE estimates when BrainAGE at two years post the initial brain scans were also estimated. This continuity of the result in longitudinal data on the same participants in a particular strength that helps boost confidence in the results. The authors also engaged in sensitivity analyses where a bunch of possibly important confounds and covariates were inserted, and the results remained robust. The authors tentatively conclude that such effects may be indicative of underlying effects of estradiol being neuroprotective and thus buffering brain health as individuals get older (e.g., the mean age at initial brain scans in this study was around 62 years of age). However, they have also been cautious to say that no direct measures of estradiol were actually measured in the datasets they analyze. The overall data and methodologies used by the authors are quite robust and the results also seem to be quite clean and sound. The effect sizes are notably small and the authors responsibly insert statements in the Discussion of the paper to talk about the notion of clinical significance of such effects and what they might mean for the actual power of estradiol in buffering brain health and aging. My only small suggestion for the authors would be to deposit anonymized variables used in the analyses for this paper along with the MRI derivatives of BrainAGE scores in a open repository as a tidy data tabular organized data file that would allow readers to easily reproduce the results reported in the study. Furthermore, the reproducible analysis code implementing the authors analyses, results, and figures could also be deposited in an openly available GitHub repository and would thus further be in line with open science practices. Currently the authors merely suggest that the data is available at the UK Biobank website, and they only provide links to software such as CAT12 and BrainAGE for the section on code availability. As a recommendation for the journal, I would recommend minor revisions. The suggestions made below are primarily useful for enhancing clarity and would likely not change anything in terms of the major impact or interpretations behind the paper.

- Thank you very much for this positive assessment. Below please find our detailed replies regarding your comments and recommendations.

The introduction is largely well written and promptly describes the bigger picture about links between neuroprotective effects of estradiol and potential importance in brain health and aging. The introduction also does a good job to spell out what BrainAGE is and how one could interpret it. This is important for readers that may not be familiar with such models predicting age based on neuroimaging data. By the end of the introduction the reader comes away with a clear understanding of the directional predictions that the authors make.

A minor suggestion to the authors would be to potentially include some statements that directly point to these kinds of effects in more highly-controlled experiments in animal models.

While human research is vitally important for giving background to this work, there is a large literature on neuroprotective effects of estradiol that could help bolster arguments about the motivations for why the authors make certain directional predictions.

- Thank you. As suggested, we extended the Introduction including information on research using animal models.

> ***Introduction***
> *[…] The risk for dementia in women is known to increase around menopause[3-6] and thus it stands to reason that estradiol plays a role for the development of dementia and other age-related neuropathologies. Indeed, studies using animal models have demonstrated that estradiol promotes synaptic plasticity, enhances neurogenesis, and protects against oxidative stress and neuroinflammation[7-13]—mechanisms that are critical for maintaining brain health and mitigating age-related brain degeneration. While extensive research has also been conducted in humans, focusing on specific phases (e.g., menarche, pregnancy, menopause) or interventions (e.g., hormonal contraceptives, menopausal estrogen therapy and anti-estrogen therapy) definitive evidence for the neuroprotective role of estradiol remains elusive[4,14-29]. Specifically, in the context of menarche and menopause, both early and late onset have been associated with an increased risk of dementia as well as with markers of brain aging and cognitive function[4,14-21,25-27]. […]*

The Methods section of the manuscript is also very clear and well written and offers engouh information for readers to clearly understand how data was selected from the UK Biobank, how the imaging data was processed, what statistical analyses were done and why, etc. The only substantive comments for the Methods would be to ask if the authors can deposit tidy tabular data of the exact data used to reproduce their results and analyses. The statement on data availability says that the MRI and other data variables are available from the UK Biobank. However, accessing the UK Biobank may incur some costs and the data is very hard to manage and process in order to reproduce the exact details of this paper. Furthermore, it would be impossible for anyone to check and try to reproduce the results of this paper, since no details are given about which data was subselected from the UK Biobank. Anonymized tidy data in tabular format that has all the variables used by the downstream final analyses could be made available, and alongside reproducible analysis code that shows how such downstream analysis was done would enable anyone to reproduce the results themselves. Thus, I would suggest more effort be given to make available the tidy derivative data for this study along with reproducible analysis code. Unless there are certain constraints from the UK Biobank that limit such data availability, this suggestion seems like it could be done without much added work.

- We appreciate this suggestion. In fact, during the submission process, we communicated with the GigaScience team regarding their data and code-sharing policies, and our proposed solutions were accepted. Specifically, the following sections were deemed suitable and added to the manuscript:

> ***Data Availability***

The individual-level proxy measures obtained from the prediction models in this work will be shared after publication in agreement with UK Biobank regulations. The input data are available to other researchers through the UK Biobank's controlled access scheme [1]. The procedure to apply for access [2] requires registering with the UK Biobank and completion of an application form detailing:

- *A summary of the planned research*
- *The UK Biobank data fields required for the project*
- *A description of derivatives (data, variables) generated by the project*

[1] Link to UK Biobank website: http://www.ukbiobank.ac.uk

[2] Link to UK Biobank access procedure: https://www.ukbiobank.ac.uk/enable-your-research/apply-for-access.

Availability of Source Code and Requirements

Project name: BrainAGE-UKBiobank
Project home page: https://github.com/ChristianGaser/BrainAGE-UKBiobank
Operating system(s): Platform independent
Programming language: Matlab
Other requirements: SPM12, CAT12, BrainAGE
License: GNU GPL-3.0

The results reported in this paper are fairly robust and highly statistically significant. However, the authors note that the effect sizes are relatively small. Would there be any other data sources that could be used in the future to possibly replicate the findings and/or extend them with actual estradiol and other measurements? This is not a question for the results section of the paper, but could be something commented on in the Discussion.

- Yes, we fully agree. Given the relatively small effect sizes, replication studies are necessary to confirm the current findings. We also believe that future studies would benefit from complementing structural/morphometric analyses with actual measures of estradiol. We have addressed this in both the Abstract and Discussion (see text excerpts below). Additionally, we have expanded the Conclusion section — also in response to your next comment — recommending the incorporation of actual estradiol and other relevant measures (see the text excerpt below your next comment).

Abstract
[…] However, further research is required because the observed effects were small, estradiol was not directly measured, and other factors may modulate female brain health. Future studies might benefit from incorporating actual estradiol (and other hormone) measures, as well as considering genetic predispositions and lifestyle factors alongside indicators of brain

aging to deepen our understanding of estradiol's role in maintaining brain health. Additionally, including more diverse study populations (e.g., varying in ethnicity, socioeconomic status, and health status) in follow-up research would enhance the generalizability and applicability of these findings.

Discussion

[…] The current findings seem to suggest a protective effect of estradiol and as such seem promising in the framework of prevention and intervention. However, further research is required, as the effect sizes for the observed associations were small (albeit smaller effect sizes are not uncommon in studies with larger sample sizes [71]) and other factors, such as genetics, life style, or hormones other than estradiol, could play a greater (or at least an additional) role in preserving brain health[2,72,73]. Moreover, our study did not measure estradiol directly, and links between estradiol and brain aging seem to be rather complex as indicated by the outcomes of other studies. For example…

The Discussion paper is very well-written and contextualized the current set of findings well. In particular, the authors are carefully to insert words of caution and note limitations with respect to small effect sizes, the issue of clinical significance, and the fact that estradiol measures were not directly measured in the UK Biobank and thus, the variables examined a proxies for estradiol as a mechanism. The Discussion might benefit for a bit more discussion on how future work could possibly follow-up on the findings reported here. Do the results reported here lead to new hypotheses to explore in the future?

- Again, thank very much for this positive evaluation. Yes, we tried to be mindful to not over interpret our findings. Following your suggestion, we have made additional revisions to the Discussion and further refined the Conclusion section to include more commentary on future research and follow-up studies.

Conclusion

Our study revealed less brain aging in women with a larger reproductive span, earlier menarche, and later menopause. Thus, sex hormones—potentially estradiol—may contribute to brain health. However, follow-up research is required because the effects observed in the current study were small, estradiol was not directly examined, and female brain health is likely modulated by factors other than estradiol. Future studies might benefit from incorporating actual estradiol (and other hormone) measurements, as well as considering genetic predispositions and lifestyle factors alongside structural brain measures. Moreover, to build a more comprehensive understanding and expand this understudied field, future research focusing on specific timeframes surrounding menopause—such as perimenopause (i.e., the time preceding the final menstrual period) or early postmenopause (e.g., the initial year after menopause) versus late menopause (e.g., ten years after menopause)—would be valuable. Lastly, the UK Biobank (i.e., the source of the current sample) is biased towards healthy and more socioeconomically privileged individuals with a predominant white ethnic background[74]. Thus, conducting research in more diverse populations, including individuals from different

ethnic, socioeconomic, and health backgrounds, would improve the generalizability of findings and provide a broader understanding of the relationship between estradiol and brain health.

Manuscript

Click here to access/download;Manuscript;R_Revised Manuscript.docx

Title:

A Case for Estradiol: Younger Brains in Women with Earlier Menarche and Later Menopause

Authors and Affiliations:

Eileen Luders[1,2,3,4*] | Inger Sundström Poromaa[1] | Claudia Barth[5] | Christian Gaser[6,7,8]

[1]Department of Women's and Children's Health, Uppsala University, Uppsala, Sweden
[2]Swedish Collegium for Advanced Study (SCAS), Uppsala, Sweden
[3]School of Psychology, University of Auckland, Auckland, New Zealand
[4]Laboratory of Neuro Imaging, School of Medicine, University of Southern California, Los Angeles, USA
[5]Department of Psychiatric Research, Diakonhjemmet Hospital, Oslo, Norway
[6]Department of Psychiatry and Psychotherapy, Jena University Hospital, Jena, Germany
[7]Department of Neurology, Jena University Hospital, Jena, Germany
[8]German Center for Mental Health (DZPG)

**Correspondence should be addressed to:*

Eileen Luders, Ph.D.

Department of Women's and Children's Health, Uppsala University, Uppsala, Sweden
Email: eileen.lueders@uu.se

Number of Words in Abstract:	**≤250**
Number of Figures:	**3**
Number of Tables:	**3**
Number of Pages:	**26**
Supplemental Material:	**3 Tables**

Date of Submission: **September 27th, 2024**

Date of Revision: **April 8, 2025**

Abstract

The transition to menopause is marked by a gradual decrease of estradiol. Concurrently, the risk of dementia in women increases around menopause, suggesting that estradiol (or the lack thereof) plays a role in the development of dementia and other age-related neuropathologies. Here, we set out to investigate whether there is a link between brain aging and estradiol-associated events, such as menarche and menopause. For this purpose, we applied a well-validated machine learning approach to analyze both cross-sectional and longitudinal data from a sample of 1,006 postmenopausal women who underwent structural magnetic resonance imaging (MRI) twice, approximately two years apart. We observed less brain aging in women with an earlier menarche, a later menopause, and a longer reproductive span (i.e., the time interval between menarche and menopause). These effects were evident both cross-sectionally and longitudinally, supporting the notion that estradiol has neuroprotective properties and contributes to brain preservation. However, further research is required because the observed effects were small, estradiol was not directly measured, and other factors may modulate female brain health. Future studies might benefit from incorporating actual estradiol (and other hormone) measures, as well as considering genetic predispositions and lifestyle factors alongside indicators of brain aging to deepen our understanding of estradiol's role in maintaining brain health. Additionally, including more diverse study populations (e.g., varying in ethnicity, socioeconomic status, and health status) in follow-up research would enhance the generalizability and applicability of these findings.

Keywords:

brain age; estradiol; machine learning; menarche; MRI; menopause; structural neuroimaging

Background

Estradiol is the most potent and prevalent form of estrogen during the reproductive life of a woman[1]. Generally speaking, estradiol levels start increasing just before the first menstrual period (menarche) and then plateau on a high level until they start decreasing during perimenopause. After the final menstrual period (i.e., menopause) estradiol levels decrease further and eventually reach plateauing low levels during postmenopause[2]. The risk for dementia in women is known to increase around menopause[3-6] and thus it stands to reason that estradiol plays a role for the development of dementia and other age-related neuropathologies. Indeed, studies using animal models have demonstrated that estradiol promotes synaptic plasticity, enhances neurogenesis, and protects against oxidative stress and neuroinflammation[7-13]—mechanisms that are critical for maintaining brain health and mitigating age-related brain degeneration. While extensive research has also been conducted in humans, focusing on specific phases (e.g., menarche, pregnancy, menopause) or interventions (e.g., hormonal contraceptives, menopausal estrogen therapy and anti-estrogen therapy), definitive evidence for the neuroprotective role of estradiol remains elusive[4,14-29]. Specifically, in the context of menarche and menopause, both early and late onset have been associated with an increased risk of dementia as well as with markers of brain aging and cognitive function[4,14-21,25-27].

To further advance this field of research, the current study set out to determine if there is a link between a woman's estimated brain age (a biological marker of brain health[30]) and the reproductive span (i.e., the interval between menarche and menopause when estradiol levels are high). If a lack of estradiol is among the driving factors for diminished brain health later in life, brain age and reproductive span should be inversely related (negative correlation). To be able to

relate our findings to others in the literature[14-16] and to provide a frame of reference for future studies, we additionally investigated if there is a significant link between estimated brain age and the age at menarche as well as the age at menopause. Assuming a neuroprotective effect of estradiol, we expected that a lower brain age would be linked to an earlier menarche (positive correlation) and to a later menopause (negative correlation). Importantly, our study comprises both cross-sectional and longitudinal components, with follow-up data acquired approximately two years after the initial brain scan.

To estimate brain age, we used structural brain images and a well-validated high-dimensional pattern recognition approach, as detailed elsewhere [31,32]. Briefly, the difference between the estimated brain age and the chronological age yields a so-called brain age gap estimate (BrainAGE) in years. The BrainAGE index is negative if a brain is estimated younger than its chronological age; it is positive if a brain is estimated older than its chronological age. For example, a 50-year-old woman with a BrainAGE index of -3 years shows the aging pattern of a 47-year-old. The BrainAGE algorithm has been shown to be robust and reliable across datasets, age-ranges, and scanner types[31,33]; it has been successfully applied in a wide range of studies[31,32,34-36] including those capturing hormonal changes in women[37,38]. Moreover, the BrainAGE index has been demonstrated to work as a predictor of dementia as well as age-related cognitive decline[34,39]. A major advantage of the BrainAGE approach is its ability to aggregate complex, spatially distributed age-related changes in brain structure into a single, interpretable biomarker. Such brain age metrics provide a powerful way to study the influence of biological factors across the female lifespan, including the effects of cumulative estrogen exposure and genetic risk for age-related brain degeneration, as also shown in other studies [14].

Materials and Methods

Data Description

The study is based on a carefully selected sample of 1,006 postmenopausal women from the UK Biobank (https://www.ukbiobank.ac.uk/) which was accessed under application number #41655. The UK Biobank is a biomedical database and research resource that contains genetic, lifestyle and health information from half a million people. In the UK Biobank cohort, 94.6% of participants are of white ethnicity[40]. For general ethnic information, see https://biobank.ctsu.ox.ac.uk/crystal/field.cgi?id=21000; for ethnic information on all women with available longitudinal data, see **Supplemental Table 3**. The UK Biobank holds the ethical approval from the North West Multi-Centre Research Ethics Committee (MREC) and is in possession of the informed consents. Written informed consent was obtained from all participants. Inclusion criteria for the current study were women with available longitudinal data as well as information on age at menarche and age at menopause. Exclusion criteria for the current study were pre-existing neurological or psychiatric diagnoses as per UK Biobank data fields #41202-0.0 to #41202-0.78. In addition, to further increase the homogeneity of the sample, we excluded women whose age at menarche was younger than 10 or older than 18, or whose age at menopause was younger than 45 or older than 60. This resulted in a final sample size of 1,006 women. **Table 1** provides information on this final sample; **Figure 1** summarizes the steps related to the sample selection. For each woman, one initial brain scan and one follow-up brain scan – approximately two years apart (mean ± SD: 2.35 ± 6.12 years) – were obtained *after* menopause. These T1-weighted brain images were acquired on a 3 Tesla Siemens Skyra scanner

using a 32-channel head coil, as described elsewhere[41] (see also: http://biobank.ctsu.ox.ac.uk/crystal/crystal/docs/bmri_V4_23092014.pdf).

– Table 1 –

– Figure 1 –

Data Analyses

Using the aforementioned T1-weighted images, we applied a number of processing routines implemented in the CAT12 toolbox[42] (version 12.8), which resulted in bias-corrected, spatially normalized, and tissue-classified brain images, as detailed elsewhere[31,38]. The normalized gray and white matter partitions were smoothed using a 4 and 8 mm full-width-at-half-maximum (FWHM) Gaussian kernel, and image resolution was set to 4 and 8 mm. For further data reduction, we applied a principal component analysis (PCA) using singular value decomposition to all the models using n–1 PCA components (n = minimum of voxel number or sample size). Prior to applying the PCA, the data were normalized by scaling the values between 0 and 1 and subtracting the mean. The transformation matrix derived from the training data PCA was used to project the normalized test data onto this principal component space.

For the estimation of the BrainAGE index, we employed a Gaussian Process Regression (GPR)[43] that uses a linear covariance function, a constant mean function, and a Gaussian likelihood function. Hyperparameters were set to 100 for the constant mean function and to -1 for the likelihood function based on prior exploratory analyses[33]. As training data, we selected 3,046 individuals from the UK Biobank where two time points were available. To avoid overfitting

and ensure generalizability, we employed 10-fold cross-validation separately for the initial and follow-up brain scan, where the dataset was randomly partitioned into 10 equally sized subsets. In each iteration, one fold was used as the test set, and the remaining nine as the training set. This process was repeated 10 times, and performance metrics (e.g., MAE) were averaged across folds. To estimate the individual brain ages, eight models based on the aforementioned sets of images (i.e., gray matter/white matter, 4 mm/8 mm Gaussian kernel, and 4 mm/8 mm image resolution) were combined using a general linear model where the weights of the models were derived by maximizing the variance to the parameter of interest (e.g., menopause). The difference between the resulting estimated brain age and the chronological age was then calculated as the BrainAGE index (in years).

Statistical Analyses

Main Analyses

After computing the BrainAGE index for all 1,006 women at initial and follow-up scan, we first removed the linear age trend that is typically seen in BrainAGE estimation. Then, we conducted two analysis streams using linear regressions in Matlab (version R2023b), one cross-sectional and one longitudinal. For all analyses, alpha was set at 0.05 (two-tailed). For the cross-sectional stream, we tested if there is a significant link between the BrainAGE index at the initial brain scan and the reproductive span. In addition, we tested if there is a significant link between the BrainAGE index at the initial brain scan and the age at menarche as well as the age at menopause. For the longitudinal stream, we first subtracted the BrainAGE index at the initial brain scan from the BrainAGE index at the follow-up brain scan, which resulted in a Δ BrainAGE

index for each individual. This method, often referred to as "change score" analysis, produces statistical results that are comparable to those resulting from a repeated-measures ANOVA with two time points. Using the Δ BrainAGE index, we then tested for significant links with the reproductive span, the age at menarche, and the age at menopause.

Sensitivity Analyses

The aforementioned main analyses were repeated while accounting for potential confounds known to affect brain health. More specifically, we removed the variance associated with the number of live births[44] (UK Biobank data field #2734), hormone replacement therapy[14] (#2814), hysterectomy[45] (#3591), bilateral oophorectomy[45] (#834), body mass index[46] (#21001), diastolic and systolic blood pressure[47] (#4079 and #4080), diabetes[48] (#2443), education[49] (#6138), income[50] (#738), and a composite lifestyle factor[51]. The latter was expressed as a general lifestyle score that was calculated based on a number of factors (see **Supplemental Table 1**), known to increase / decrease the risk of adverse cardiovascular events. Since not all women had information on all potential confounds (see **Supplemental Table 2**), we applied an imputation method using the Matlab function 'fillmissing'. That is, missing entries were replaced with the corresponding values from the nearest neighbor rows, calculated based on the pairwise Euclidean distance between rows. Imputation was applied to up to 295 women, depending on the potential confound. For the cross-sectional stream, we tested if there is a significant link between the BrainAGE index at the initial brain scan and the reproductive span (age at menarche and age at menopause, respectively). Likewise, for the longitudinal stream, we tested if there is

a significant link between the Δ BrainAGE index and the reproductive span (age at menarche and age at menopause, respectively).

Results

Main Analyses

As shown in **Figure 2** (left), our cross-sectional analyses revealed a significant negative association between BrainAGE and the reproductive span. In other words, brains of women with longer reproductive spans were estimated younger than brains of women with shorter reproductive spans. As also shown in **Figure 2** (right), there was a significant positive association between BrainAGE and age at menarche (i.e., the earlier the menarche, the younger the brain) and a significant negative association between BrainAGE and age at menopause (i.e., the later the menopause, the younger the brain). As shown in **Table 2** (main analyses), effect sizes were small[52], with r-values at -0.11, 0.14, and -0.09 for reproductive span, menarche, and menopause, respectively). The slopes of the regression indicate different rates of change for menarche and menopause (0.32 and -0.10, respectively). More specifically, for each year younger at menarche, brains are estimated 0.32 years younger (which corresponds to 3.2 years younger for each 10 years). In contrast, for each year older at menopause, brains are estimated 0.1 year younger (which corresponds to 1 year younger for each 10 years).

– Figure 2 –

– Table 2 –

As shown in **Figure 3** and **Table 3** (main analyses), our longitudinal findings confirm the observed cross-sectional relationships. More specifically, Δ BrainAGE was negatively linked to reproductive span and menopause, and positively linked to age at menarche. All associations were significant. Again, effect sizes were small, with r-values at -0.12, 0.06, and -0.12 for reproductive span, menarche, and menopause, respectively. The slopes of the regression are still somewhat different for menarche and menopause (0.08 and -0.06, respectively), albeit more similar than in the cross-sectional analysis: For each year younger at menarche, brains are estimated 0.08 years younger (0.8 years over 10 years), whereas for each year older at menopause, brains are estimated 0.06 years younger (0.6 years over 10 years).

– Figure 3 –

– Table 3 –

Sensitivity Analyses

The results described above remained comparable when removing the variance associated with the number of live births, hormone replacement therapy, hysterectomy, bilateral oophorectomy, body mass index, diastolic and systolic blood pressure, diabetes, education, income, and a composite lifestyle factor. In other words, when examining the association between BrainAGE and reproductive span, we observed a negative association. Likewise, there was a positive association between BrainAGE and age at menarche and a negative association between

BrainAGE and age at menopause. The effects were significant for reproductive span, menarche, and menopause for the cross-sectional analyses (see **Table 2**; Sensitivity Analyses), and for reproductive span and menopause for the longitudinal (see **Table 3**; Sensitivity Analyses).

Discussion

Here we assessed links between estimated brain age and milestones in a woman's reproductive life in a well-powered sample of more than a thousand postmenopausal women. We detected less brain aging in women with longer reproductive spans, earlier menarche, and later menopause (see **Figures 2-3** and **Tables 2-3**).

Correspondence with Previous Findings

Our findings are in line with the outcomes of other studies suggesting a longer reproductive span [15,17,20], an earlier menarche[20,21], as well as a later menopause [4,15,17-19] to be associated with a lower risk of developing dementia or better retained cognitive function. Furthermore, given that the BrainAGE index is based on the weighted distribution of gray and whiter matter tissue in the brain, our findings are also in agreement with reports of lower brain volumes as well as higher rates of brain tissue loss during menopause compared to premenopause or in postmenopausal women compared to premenopausal women[53-55]. In addition, our findings agree with observed effects across the menstrual cycle linking high estradiol levels at ovulation to lower BrainAGE estimates[37]. Altogether, the outcomes of our study seem to suggest that estradiol contributes to brain health, which is in agreement with other studies reporting positive effects of estradiol on

brain health and cognition within the framework of aging and/or menopausal hormone therapy[56-60].

Menarche versus Menopause

The outcomes of the main analyses indicate that both, an earlier menarche and a later menopause, are significantly associated with less brain aging. However, menarche and menopause differ with respect to the strength of their relationship with age (which is reflected in the correlation coefficient) and their rate of change with age (which is reflected in the slope of the regression line). This might indicate somewhat different underlying biological mechanisms and/or confounds for menarche and menopause. For example, during menopause, in addition to decreasing levels of estradiol, increasing levels of follicle-stimulating hormones may cause an accelerated deposition of amyloid-β and Tau[61], which enhances brain atrophy. Moreover, menopause is marked by disadvantageous alterations in cytokine and T cell profiles[62] , which are linked to an enhanced inflammation. Alternatively, the less strong link pertaining to menarche could also reflect the fact that, later in life, it is probably more challenging to accurately remember the onset of menarche than the onset of menopause.

Potential Implications

Given that estradiol levels start decreasing during perimenopause and further decrease after menopause, our findings may explain why the risk for dementia in women is known to increase around menopause[3-6] and why there is an increased age-independent prevalence of Alzheimer's disease in women compared to men[60]. Moreover, our findings seem to support to the concept

of the “window of opportunity”, spanning the years leading up to menopause to the years immediately after menopause, where health interventions (e.g., menopausal hormone treatment) may combat the increased risk for Alzheimer's disease in some women[5,63-65]. In fact, several large-scale projects have investigated the effects of menopausal hormone treatment on cognitive function and Alzheimer's risk, but results are inconclusive (potentially relevant modulators of treatment outcomes are discussed elsewhere[56,66-71]).

The current findings seem to suggest a protective effect of estradiol and as such seem promising in the framework of prevention and intervention. However, further research is required, as the effect sizes for the observed associations were small (albeit smaller effect sizes are not uncommon in studies with larger sample sizes [72]) and various factors, such as genetics, life style, or hormones other than estradiol, could play a greater (or at least an additional) role in preserving brain health[2,73,74]. Moreover, our study did not measure estradiol directly, and links between estradiol and brain aging seem to be rather complex as indicated by the outcomes of other studies. For example, it was reported that, compared to no exposure or no dose, exposure to low concentrations of estradiol or low doses of estrogen enhanced neuronal survival and increased anti-inflammatory markers (i.e., positive links), while exposure to high concentrations of estradiol as well as high doses of estrogen had the opposite effect (i.e., negative links)[28,29]. Another study reported U-shaped curves suggesting that both early and late menarche are associated with an increased risk for dementia (i.e., positive and negative links)[15]. And yet another study reported either negative links or missing links between age at menarche and brain aging depending on the potential confounds accounted for[14]. Interestingly, this latter study also reported that, in carriers of the apolipoprotein E type 4 allele (APOE e4), higher levels of estradiol

at menopause were associated with increased brain aging (positive link). In contrast, in non-carriers, higher levels of estradiol at menopause were associated with decreased brain aging (negative link)[14].

Conclusion

Our study revealed less brain aging in women with a larger reproductive span, earlier menarche, and later menopause. Thus, sex hormones—potentially estradiol—may contribute to brain health. However, follow-up research is required because the effects observed in the current study were small, estradiol was not directly examined, and female brain health is likely modulated by factors other than estradiol. Future studies might benefit from incorporating actual estradiol (and other hormone) measurements, as well as considering genetic predispositions and lifestyle factors alongside structural brain measures. Moreover, to build a more comprehensive understanding and expand this understudied field, future research focusing on specific timeframes surrounding menopause—such as perimenopause (i.e., the time preceding the final menstrual period) or early postmenopause (e.g., the initial year after menopause) versus late menopause (e.g., ten years after menopause)—would be valuable. Lastly, the UK Biobank (i.e., the source of the current sample) is biased towards healthy and more socioeconomically privileged individuals with a predominant white ethnic background[40].

Thus, conducting research in more diverse populations, including individuals from different ethnic, socioeconomic, and health backgrounds, would improve the generalizability of findings and provide a broader understanding of the relationship between estradiol and brain health.

Acknowledgments

EL was supported by the Swedish Collegium for Advanced Study (SCAS) and Erling-Persson Family Foundation. The data of the UK Biobank were accessed under application number #41655. The article was composed using the STROBE cohort checklist[75].

Funding

CB received funding from the South-Eastern Norway Regional Health Authority (2023037, 2022103).

Data Availability

The individual-level proxy measures obtained from the prediction models in this work will be shared after publication in agreement with UK Biobank regulations. The input data are available to other researchers through the UK Biobank's controlled access scheme [1]. The procedure to apply for access [2] requires registering with the UK Biobank and completion of an application form detailing:

- A summary of the planned research
- The UK Biobank data fields required for the project
- A description of derivatives (data, variables) generated by the project

[1] Link to UK Biobank website: http://www.ukbiobank.ac.uk

[2] Link to UK Biobank access procedure: https://www.ukbiobank.ac.uk/enable-your-research/apply-for-access.

Availability of Source Code and Requirements

Project name: BrainAGE-UKBiobank

Project home page: https://github.com/ChristianGaser/BrainAGE-UKBiobank

Operating system(s): Platform independent

Programming language: Matlab

Other requirements: SPM12, CAT12, BrainAGE

License: GNU GPL-3.0

Competing Interests

The authors declare that the research was conducted in the absence of any commercial or financial relationships that could be construed as a potential conflict of interest.

References

1. Thomas, M.P. & Potter, B.V. The structural biology of oestrogen metabolism. *The Journal of steroid biochemistry and molecular biology* **137**, 27-49 (2013).
2. Barth, C., Crestol, A., de Lange, A.G. & Galea, L.A.M. Sex steroids and the female brain across the lifespan: insights into risk of depression and Alzheimer's disease. *Lancet Diabetes Endocrinol* **11**, 926-941 (2023).
3. Farrer, L.A.*, et al.* Effects of age, sex, and ethnicity on the association between apolipoprotein E genotype and Alzheimer disease. A meta-analysis. APOE and Alzheimer Disease Meta Analysis Consortium. *JAMA* **278**, 1349-1356 (1997).
4. Brinton, R.D., Yao, J., Yin, F., Mack, W.J. & Cadenas, E. Perimenopause as a neurological transition state. *Nat Rev Endocrinol* **11**, 393-405 (2015).
5. Mosconi, L.*, et al.* Increased Alzheimer's risk during the menopause transition: A 3-year longitudinal brain imaging study. *PLoS One* **13**, e0207885 (2018).
6. Rahman, A.*, et al.* Sex-driven modifiers of Alzheimer risk: A multimodality brain imaging study. *Neurology* **95**, e166-e178 (2020).
7. Vegeto, E., Benedusi, V. & Maggi, A. Estrogen anti-inflammatory activity in brain: a therapeutic opportunity for menopause and neurodegenerative diseases. *Front Neuroendocrinol* **29**, 507-519 (2008).
8. Behl, C., Widmann, M., Trapp, T. & Holsboer, F. 17-beta estradiol protects neurons from oxidative stress-induced cell death in vitro. *Biochem Biophys Res Commun* **216**, 473-482 (1995).
9. Barha, C.K., Lieblich, S.E. & Galea, L.A. Different forms of oestrogen rapidly upregulate cell proliferation in the dentate gyrus of adult female rats. *J Neuroendocrinol* **21**, 155-166 (2009).
10. Tanapat, P., Hastings, N.B., Reeves, A.J. & Gould, E. Estrogen stimulates a transient increase in the number of new neurons in the dentate gyrus of the adult female rat. *The Journal of neuroscience : the official journal of the Society for Neuroscience* **19**, 5792-5801 (1999).
11. Inagaki, T., Kaneko, N., Zukin, R.S., Castillo, P.E. & Etgen, A.M. Estradiol attenuates ischemia-induced death of hippocampal neurons and enhances synaptic transmission in aged, long-term hormone-deprived female rats. *PLoS One* **7**, e38018 (2012).
12. Woolley, C.S. & McEwen, B.S. Estradiol mediates fluctuation in hippocampal synapse density during the estrous cycle in the adult rat. *The Journal of neuroscience : the official journal of the Society for Neuroscience* **12**, 2549-2554 (1992).
13. Yankova, M., Hart, S.A. & Woolley, C.S. Estrogen increases synaptic connectivity between single presynaptic inputs and multiple postsynaptic CA1 pyramidal cells: a serial electron-microscopic study. *Proc.Natl.Acad.Sci.U.S.A* **98**, 3525-3530 (2001).
14. de Lange, A.G.*, et al.* Women's brain aging: Effects of sex-hormone exposure, pregnancies, and genetic risk for Alzheimer's disease. *Hum Brain Mapp* **41**, 5141-5150 (2020).
15. Gong, J., Harris, K., Peters, S.A.E. & Woodward, M. Reproductive factors and the risk of incident dementia: A cohort study of UK Biobank participants. *PLoS Med* **19**, e1003955 (2022).
16. Jani, M.*, et al.* Birth outcomes, puberty onset, and obesity as long-term predictors of biological aging in young adulthood. *Front Nutr* **9**, 1100237 (2022).
17. Lindseth, L.R.S.*, et al.* Associations between reproductive history, hormone use, APOE epsilon4 genotype and cognition in middle- to older-aged women from the UK Biobank. *Front Aging Neurosci* **14**, 1014605 (2022).
18. Kuh, D., Cooper, R., Moore, A., Richards, M. & Hardy, R. Age at menopause and lifetime cognition: Findings from a British birth cohort study. *Neurology* **90**, e1673-e1681 (2018).
19. McLay, R.N., Maki, P.M. & Lyketsos, C.G. Nulliparity and late menopause are associated with decreased cognitive decline. *J Neuropsychiatry Clin Neurosci* **15**, 161-167 (2003).

20. Karim, R., *et al.* Effect of Reproductive History and Exogenous Hormone Use on Cognitive Function in Mid- and Late Life. *J Am Geriatr Soc* **64**, 2448-2456 (2016).
21. Ryan, J., Carriere, I., Scali, J., Ritchie, K. & Ancelin, M.L. Life-time estrogen exposure and cognitive functioning in later life. *Psychoneuroendocrinology* **34**, 287-298 (2009).
22. Branigan, G.L., Soto, M., Neumayer, L., Rodgers, K. & Brinton, R.D. Association Between Hormone-Modulating Breast Cancer Therapies and Incidence of Neurodegenerative Outcomes for Women With Breast Cancer. *JAMA Netw Open* **3**, e201541 (2020).
23. Comasco, E., Frokjaer, V.G. & Sundstrom-Poromaa, I. Functional and molecular neuroimaging of menopause and hormone replacement therapy. *Frontiers in neuroscience* **8**, 388 (2014).
24. Nabulsi, L., *et al.* Exogenous Sex Hormone Effects on Brain Microstructure in Women: A diffusion MRI Study in the UK Biobank. *bioRxiv : the preprint server for biology*, 2020.2009.2018.304154 (2023).
25. Georgakis, M.K., *et al.* Age at menopause and duration of reproductive period in association with dementia and cognitive function: A systematic review and meta-analysis. *Psychoneuroendocrinology* **73**, 224-243 (2016).
26. Ambikairajah, A., Tabatabaei-Jafari, H., Hornberger, M. & Cherbuin, N. Age, menstruation history, and the brain. *Menopause* **28**, 167-174 (2020).
27. Gilsanz, P., *et al.* Reproductive period and risk of dementia in a diverse cohort of health care members. *Neurology* **92**, e2005-e2014 (2019).
28. Prestwood, K.M., Unson, C., Kulldorff, M. & Cushman, M. The effect of different doses of micronized 17beta-estradiol on C-reactive protein, interleukin-6, and lipids in older women. *J Gerontol A Biol Sci Med Sci* **59**, 827-832 (2004).
29. Chen, S., Nilsen, J. & Brinton, R.D. Dose and temporal pattern of estrogen exposure determines neuroprotective outcome in hippocampal neurons: therapeutic implications. *Endocrinology* **147**, 5303-5313 (2006).
30. Franke, K. & Gaser, C. Ten Years of BrainAGE as a Neuroimaging Biomarker of Brain Aging: What Insights Have We Gained? *Front Neurol* **10**, 789 (2019).
31. Franke, K., Ziegler, G., Kloppel, S. & Gaser, C. Estimating the age of healthy subjects from T1-weighted MRI scans using kernel methods: exploring the influence of various parameters. *Neuroimage* **50**, 883-892 (2010).
32. Franke, K., Luders, E., May, A., Wilke, M. & Gaser, C. Brain maturation: Predicting individual BrainAGE in children and adolescents using structural MRI. *Neuroimage* **63**, 1305-1312 (2012).
33. Kalc, P., Dahnke, R., Hoffstaedter, F. & Gaser, C. BrainAGE: Revisited and reframed machine learning workflow. *Hum Brain Mapp* **45**, e26632 (2024).
34. Gaser, C., Franke, K., Kloppel, S., Koutsouleris, N. & Sauer, H. BrainAGE in Mild Cognitive Impaired Patients: Predicting the Conversion to Alzheimer's Disease. *PLoS One* **8**, e67346 (2013).
35. Franke, K., Ristow, M. & Gaser, C. Gender-specific impact of personal health parameters on individual brain aging in cognitively unimpaired elderly subjects. *Front Aging Neurosci* **6**, 94 (2014).
36. Luders, E., Cherbuin, N. & Gaser, C. Estimating brain age using high-resolution pattern recognition: Younger brains in long-term meditation practitioners. *NeuroImage* **134**, 508-513 (2016).
37. Franke, K., Hagemann, G., Schleussner, E. & Gaser, C. Changes of individual BrainAGE during the course of the menstrual cycle. *NeuroImage* **115**, 1-6 (2015).
38. Luders, E., *et al.* Potential Brain Age Reversal after Pregnancy: Younger Brains at 4-6Weeks Postpartum. *Neuroscience* **386**, 309-314 (2018).
39. Giannakopoulos, P., *et al.* Alzheimer resemblance atrophy index, BrainAGE, and normal pressure hydrocephalus score in the prediction of subtle cognitive decline: added value compared to existing MR imaging markers. *Eur Radiol* (2022).

40. Fry, A., *et al.* Comparison of Sociodemographic and Health-Related Characteristics of UK Biobank Participants With Those of the General Population. *Am J Epidemiol* **186**, 1026-1034 (2017).
41. Alfaro-Almagro, F., *et al.* Image processing and Quality Control for the first 10,000 brain imaging datasets from UK Biobank. *NeuroImage* **166**, 400-424 (2018).
42. Gaser, C., Dahnke, R., Thompson, P.M., Kurth, F. & Luders, E. CAT – A Computational Anatomy Toolbox for the Analysis of Structural MRI Data. *bioRxiv* **2022.06.11.495736**(2022).
43. Rasmussen, C.E.W., C. K. I. Gaussian Processes for Machine Learning. *MIT Press* (2006).
44. de Lange, A.G., *et al.* Population-based neuroimaging reveals traces of childbirth in the maternal brain. *Proc Natl Acad Sci U S A* **116**, 22341-22346 (2019).
45. Rocca, W.A., Grossardt, B.R., Shuster, L.T. & Stewart, E.A. Hysterectomy, oophorectomy, estrogen, and the risk of dementia. *Neurodegener Dis* **10**, 175-178 (2012).
46. Tungler, A., *et al.* Body mass index but not genetic risk is longitudinally associated with altered structural brain parameters. *Scientific reports* **11**, 24246 (2021).
47. Cherbuin, N., *et al.* Optimal Blood Pressure Keeps Our Brains Younger. *Front Aging Neurosci* **13**, 694982 (2021).
48. Antal, B., *et al.* Type 2 diabetes mellitus accelerates brain aging and cognitive decline: Complementary findings from UK Biobank and meta-analyses. *Elife* **11**(2022).
49. Chan, M.Y., *et al.* Long-term prognosis and educational determinants of brain network decline in older adult individuals. *Nat Aging* **1**, 1053-1067 (2021).
50. Busby, N., *et al.* Lower socioeconomic status is associated with premature brain aging. *Neurobiology of aging* **130**, 135-140 (2023).
51. Foster, H.M.E., *et al.* The effect of socioeconomic deprivation on the association between an extended measurement of unhealthy lifestyle factors and health outcomes: a prospective analysis of the UK Biobank cohort. *Lancet Public Health* **3**, e576-e585 (2018).
52. Cohen, J. A power primer. *Psychol.Bull.* **112**, 155-159 (1992).
53. Goto, M., *et al.* Accelerated hippocampal volume reduction in post-menopausal women: an additional study with Atlas-based method. *Radiol Phys Technol* **4**, 185-188 (2011).
54. Goto, M., *et al.* 3 Tesla MRI detects accelerated hippocampal volume reduction in postmenopausal women. *J Magn Reson Imaging* **33**, 48-53 (2011).
55. Lu, W., *et al.* Grey matter differences associated with age and sex hormone levels between premenopausal and perimenopausal women: A voxel-based morphometry study. *J Neuroendocrinol* **30**, e12655 (2018).
56. Manly, J.J., *et al.* Endogenous estrogen levels and Alzheimer's disease among postmenopausal women. *Neurology* **54**, 833-837 (2000).
57. Boccardi, M., *et al.* Effects of hormone therapy on brain morphology of healthy postmenopausal women: a Voxel-based morphometry study. *Menopause* **13**, 584-591 (2006).
58. Ghidoni, R., *et al.* Effects of estrogens on cognition and brain morphology: involvement of the cerebellum. *Maturitas* **54**, 222-228 (2006).
59. Kim, T.H., Kim, B., Kim, Y.R., Jeong, C.W. & Lee, Y.H. Gray matter differences associated with menopausal hormone therapy in menopausal women: a DARTEL-based VBM study. *Scientific reports* **13**, 1401 (2023).
60. Depypere, H., *et al.* Menopause hormone therapy significantly alters pathophysiological biomarkers of Alzheimer's disease. *Alzheimers Dement* **19**, 1320-1330 (2023).
61. Xiong, J., *et al.* FSH blockade improves cognition in mice with Alzheimer's disease. *Nature* **603**, 470-476 (2022).
62. Mishra, A. & Brinton, R.D. Inflammation: Bridging Age, Menopause and APOEepsilon4 Genotype to Alzheimer's Disease. *Front Aging Neurosci* **10**, 312 (2018).

63. Scheyer, O., *et al.* Female Sex and Alzheimer's Risk: The Menopause Connection. *J Prev Alzheimers Dis* **5**, 225-230 (2018).
64. Davey, D.A. Alzheimer's disease, dementia, mild cognitive impairment and the menopause: a 'window of opportunity'? *Womens Health (Lond)* **9**, 279-290 (2013).
65. Mishra, A., *et al.* A tale of two systems: Lessons learned from female mid-life aging with implications for Alzheimer's prevention & treatment. *Ageing Res Rev* **74**, 101542 (2022).
66. Middleton, L.E. & Yaffe, K. Promising strategies for the prevention of dementia. *Arch Neurol* **66**, 1210-1215 (2009).
67. Marder, K. & Sano, M. Estrogen to treat Alzheimer's disease: too little, too late? So what's a woman to do? *Neurology* **54**, 2035-2037 (2000).
68. Resnick, S.M. & Henderson, V.W. Hormone therapy and risk of Alzheimer disease: a critical time. *JAMA* **288**, 2170-2172 (2002).
69. Yaffe, K. Estrogens, selective estrogen receptor modulators, and dementia: what is the evidence? *Ann N Y Acad Sci* **949**, 215-222 (2001).
70. Yaffe, K., Haan, M., Byers, A., Tangen, C. & Kuller, L. Estrogen use, APOE, and cognitive decline: evidence of gene-environment interaction. *Neurology* **54**, 1949-1954 (2000).
71. Nerattini, M., *et al.* Systematic review and meta-analysis of the effects of menopause hormone therapy on risk of Alzheimer's disease and dementia. *Front Aging Neurosci* **15**, 1260427 (2023).
72. Marek, S., *et al.* Reproducible brain-wide association studies require thousands of individuals. *Nature* **603**, 654-660 (2022).
73. Barth, C. & de Lange, A.G. Towards an understanding of women's brain aging: the immunology of pregnancy and menopause. *Front Neuroendocrinol* **58**, 100850 (2020).
74. Barth, C., Villringer, A. & Sacher, J. Sex hormones affect neurotransmitters and shape the adult female brain during hormonal transition periods. *Frontiers in neuroscience* **9**(2015).
75. von Elm, E., *et al.* The Strengthening the Reporting of Observational Studies in Epidemiology (STROBE) statement: guidelines for reporting observational studies. *J Clin Epidemiol* **61**, 344-349 (2008).

Figure Legends

Figure 1. Flowchart of sample selection.

Figure 2. Correlations with BrainAGE at the initial brain scan. The x-axes show the reproductive span (age, respectively) in years. Of note, age in the UK Biobank has been rounded to the year, so we added a small random jitter to the x-axes to give a better overview about the age distribution. The y-axes show the BrainAGE index in years, with negative values indicating that brains are estimated younger than their chronological age and positive values indicating that brains are estimated older than their chronological age. Panel A displays a negative link between the BrainAGE index and the reproductive span (the longer the reproductive span, the younger the estimated brain age). Panel B displays a positive link between the BrainAGE index and the age at menarche (the earlier the onset of menarche, the younger the estimated brain age). Panel C displays a negative link between the BrainAGE index and the age at menopause (the later the onset of menopause, the younger the estimated brain age). The squares in the density plot represent the individual measures (n=1,006); hot colors indicate a larger overlay of measures; cool colors indicate a smaller overlay. The shaded band is the 95% confidence interval.

Figure 3. Correlations with BrainAGE over 2.35 years (Δ BrainAGE). Panel A displays a negative link between the BrainAGE index and reproductive span (the longer the reproductive span, the smaller the estimated brain age). Panel B displays a positive link between the BrainAGE index and the age at menarche (the earlier the onset of menarche, the younger the estimated brain age). Panel C displays a negative link between the BrainAGE index and the age at menopause (the later the onset of menopause, the younger the estimated brain age). The squares in the density plot represent the individual measures (n=1,006); hot colors indicate a larger overlay of measures; cool colors indicate a smaller overlay. The shaded band is the 95% confidence interval.

Tables

Table 1. Sample characteristics

Variable	Descriptive Statistics
Age at the initial brain scan	mean ± SD: 63.20 ± 6.42 years
Age at the follow-up brain scan	mean ± SD: 65.54 ± 6.37 years
Age at menarche	mean ± SD: 13.02 ± 1.53 years
Age at menopause	mean ± SD: 51.41 ± 3.23 years
Reproductive span	mean ± SD: 38.39 ± 3.55 years
Number of live births	mean ± SD: 1.75 ± 1.16
Number of women with hormone replacement therapy	yes: 306 (30.42%) | no: 700 (69.58%)
Number of women with hysterectomy	yes: 48 (4.77%) | no: 958 (95.23%)
Number of women with bilateral oophorectomy	yes: 37 (3.68%)| no: 969 (96.32%)

SD = standard deviation

Table 2. Associations with BrainAGE at the initial brain scan

	Main Analyses					Sensitivity Analyses*				
	R^2	r	p	Slope	95% Confidence Interval	R^2	r	p	Slope	95% Confidence Interval
Reproductive Span	0.01	-0.11	<0.001	-0.11	-0.17 to -0.05	0.01	-0.11	<0.001	-0.11	-0.17 to -0.05
Age at Menarche	0.02	0.14	<0.001	0.32	0.18 to 0.46	0.02	0.14	<0.001	0.33	0.19 to 0.47
Age at Menopause	0.01	-0.09	<0.005	-0.10	-0.17 to -0.03	0.01	-0.09	<0.01	-0.09	-0.16 to -0.03

*While removing the variance associated with the number of live births, hormone replacement therapy, hysterectomy, bilateral oophorectomy, body mass index, diastolic and systolic blood pressure, diabetes, education, income, and a composite lifestyle factor.

Table 3. Associations with changes in BrainAGE over 2.35 years

	Main Analyses					Sensitivity Analyses*				
	R^2	r	p	Slope	95% Confidence Interval	R^2	r	p	Slope	95% Confidence Interval
Reproductive Span	0.01	-0.12	<0.001	-0.06	-0.10 to -0.03	0.01	-0.11	<0.001	-0.06	-0.09 to -0.03
Age at Menarche	<0.01	0.06	<0.05	0.08	0.0 to 0.16	<0.01	0.06	n.s.	0.08	0.0 to 0.16
Age at Menopause	0.01	-0.12	<0.001	-0.07	-0.10 to -0.03	0.01	-0.12	<0.001	-0.07	-0.11 to -0.03

*While removing the variance associated with the number of live births, hormone replacement therapy, hysterectomy, bilateral oophorectomy, body mass index, diastolic and systolic blood pressure, diabetes, education, income, and a composite lifestyle factor.
n.s. = not significant

Figure 1

Click here to access/download;Figure;Figure 1.png

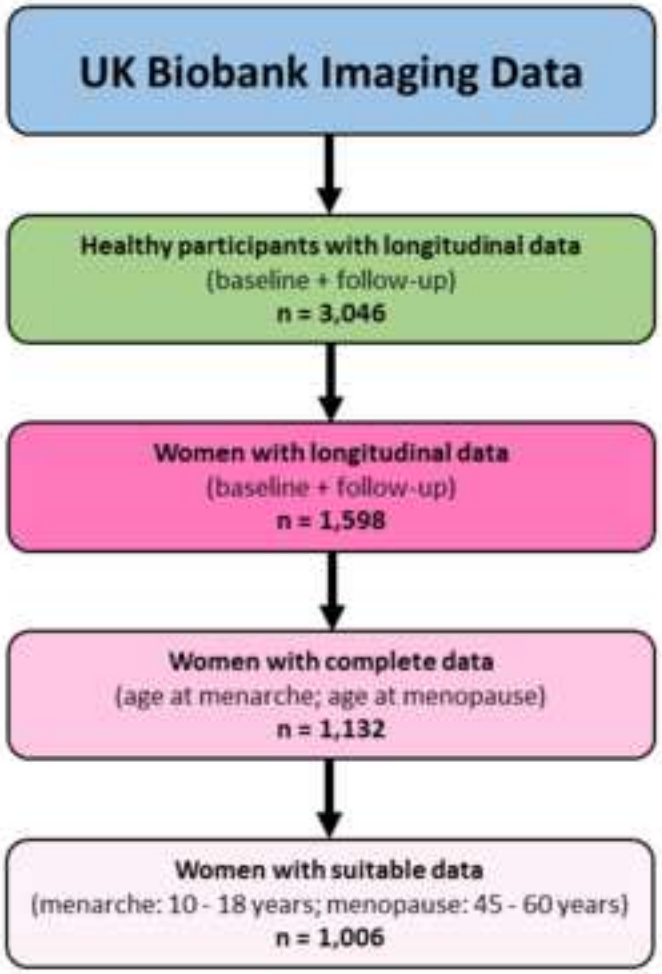

Figure 2

Click here to access/download;Figure;Figure 2.png

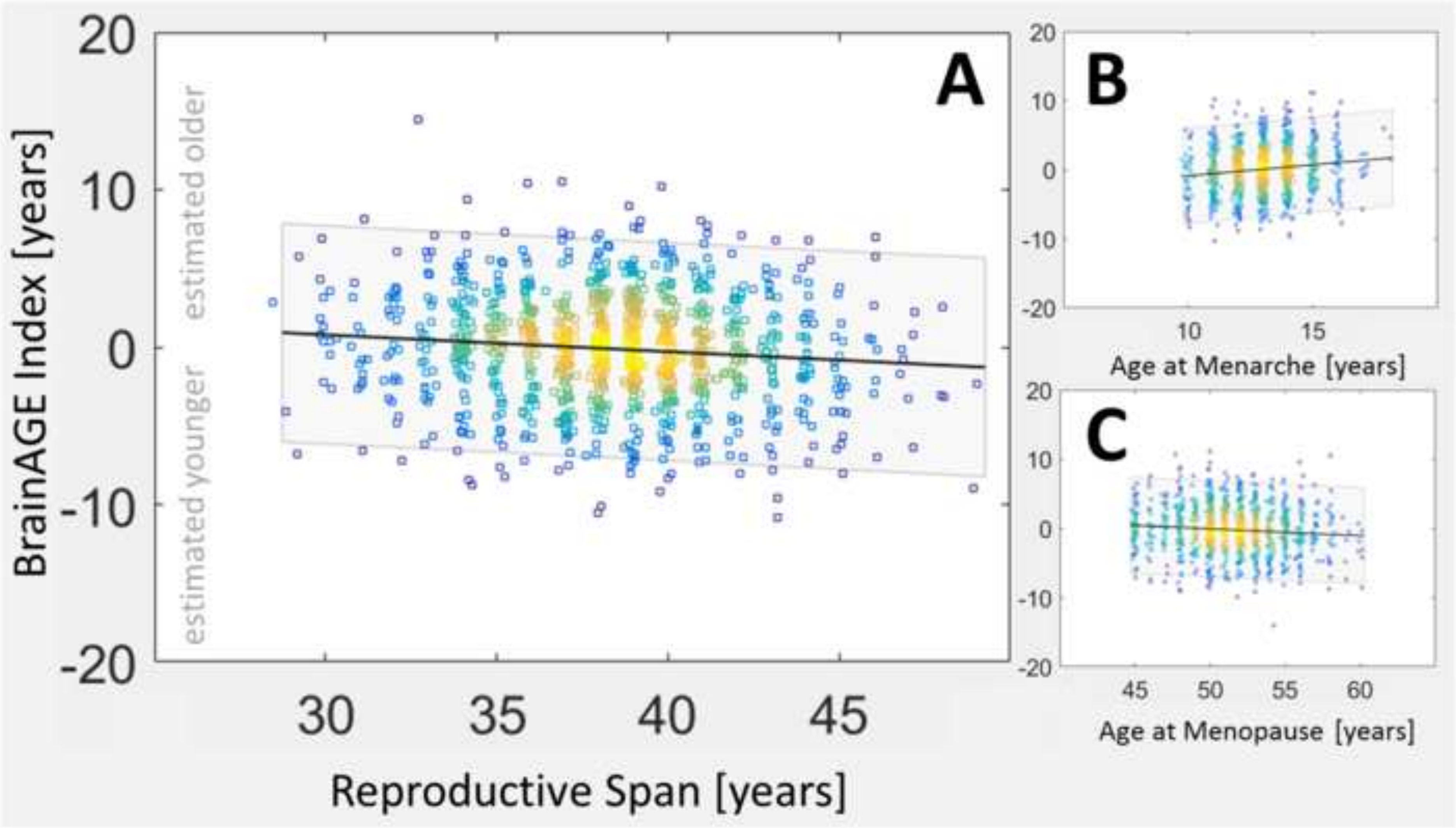

Figure 3

Click here to access/download;Figure;Figure 3.png

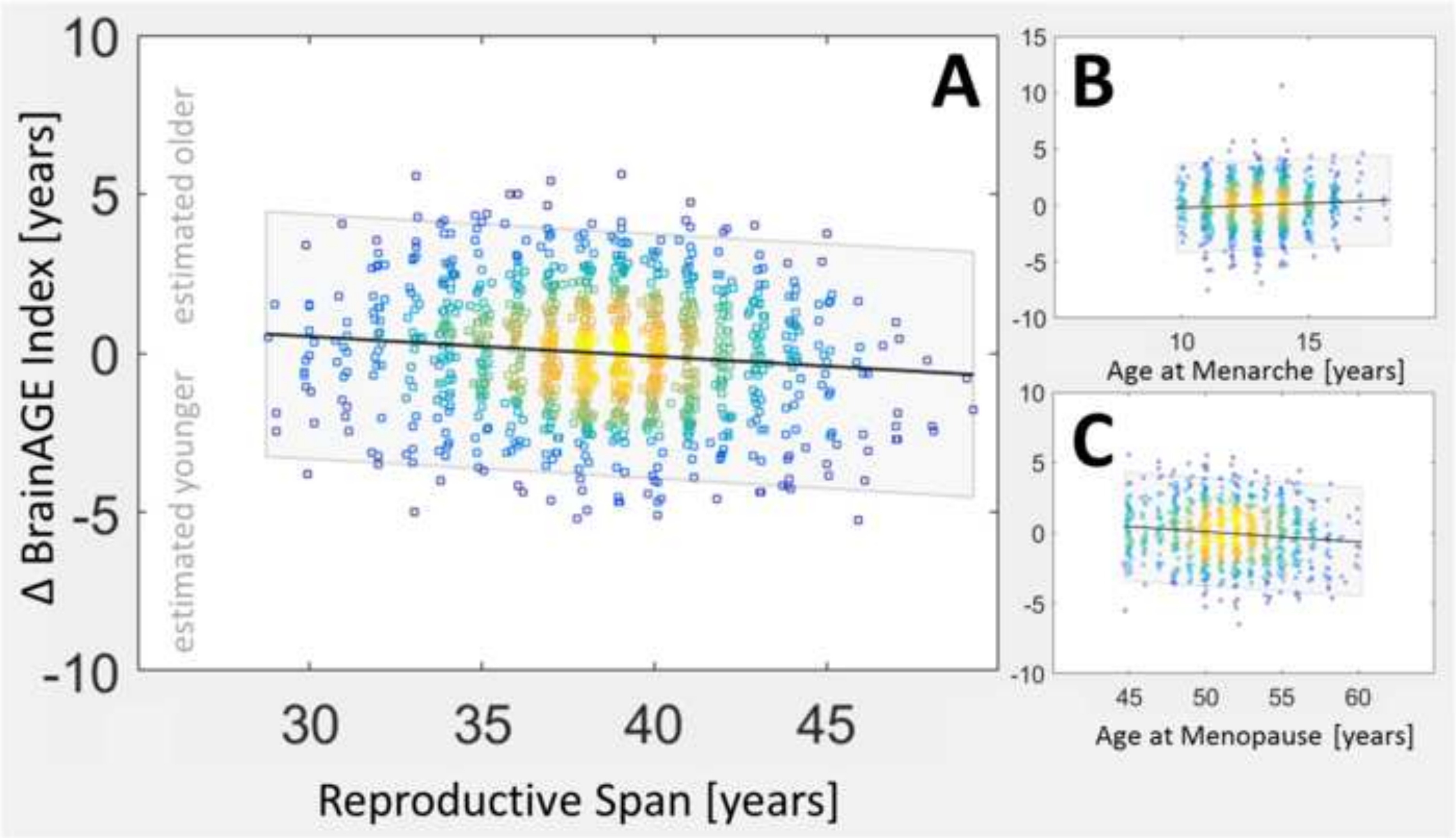

Supplementary Tables

Click here to access/download
Supplementary Material
R_Supplemental Tables.docx
