# Supplementary material for: A Case for estradiol: younger brains in women with earlier menarche and later menopause
Source: Gigascience. 2025 May 23;14:giaf060. doi: 10.1093/gigascience/giaf060 (PMC12099614; doi:10.1093/gigascience/giaf060)
Supplement: giaf060_GIGA-D-24-00418_Revision_2 [file giaf060_giga-d-24-00418_revision_2.pdf]

GigaScience

A Case for Estradiol: Younger Brains in Women with Earlier Menarche and Later Menopause

--Manuscript Draft--

Manuscript Number:	GIGA-D-24-00418R2
Full Title:	A Case for Estradiol: Younger Brains in Women with Earlier Menarche and Later Menopause
Article Type:	Research
Funding Information:	Helse Sør-Øst RHF (2023037, 2022103) / Dr. Claudia Barth
Abstract:	The transition to menopause is marked by a gradual decrease of estradiol. At the same time, the risk of dementia increases around menopause and it stands to reason that estradiol (or the lack thereof) plays a role for the development of dementia and other age-related neuropathologies. Here we investigated if there is a link between brain aging and estradiol-associated events, such as menarche and menopause. For this purpose, we applied a well-validated machine learning approach in a sample of 1,006 postmenopausal women who were scanned twice approximately two years apart. We observed less brain aging in women with an earlier menarche, a later menopause, and a longer reproductive span (i.e., the time interval between menarche and menopause). These effects were evident both cross-sectionally and longitudinally, which supports the notion that estradiol contributes to brain preservation. However, further research is required because no direct measures of estradiol were obtained and effects were small overall.
Corresponding Author:	Eileen Luders<br>Uppsala Universitet<br>Uppsala, SWEDEN
Corresponding Author Secondary Information:	
Corresponding Author's Institution:	Uppsala Universitet
Corresponding Author's Secondary Institution:	
First Author:	Eileen Luders
First Author Secondary Information:	
Order of Authors:	Eileen Luders
	Inger Sundström Poromaa
	Claudia Barth
	Christian Gaser
Order of Authors Secondary Information:	
Response to Reviewers:	We have re-formatted the document as per the editor's comments. Thank you!
Additional Information:	
Question	Response
Are you submitting this manuscript to a special series or article collection?	No
Experimental design and statistics<br><br>Full details of the experimental design and	Yes

statistical methods used should be given in the Methods section, as detailed in our Minimum Standards Reporting Checklist. Information essential to interpreting the data presented should be made available in the figure legends.<br><br>Have you included all the information requested in your manuscript?	
**Resources**<br><br>A description of all resources used, including antibodies, cell lines, animals and software tools, with enough information to allow them to be uniquely identified, should be included in the Methods section. Authors are strongly encouraged to cite Research Resource Identifiers (RRIDs) for antibodies, model organisms and tools, where possible.<br><br>Have you included the information requested as detailed in our Minimum Standards Reporting Checklist?	Yes
**Availability of data and materials**<br><br>All datasets and code on which the conclusions of the paper rely must be either included in your submission or deposited in publicly available repositories (where available and ethically appropriate), referencing such data using a unique identifier in the references and in the "Availability of Data and Materials" section of your manuscript.<br><br>Have you have met the above requirement as detailed in our Minimum Standards Reporting Checklist?	Yes
GigaScience has policies and guidelines in place for the use of generative AI-writing tools such as ChatGPT. If you have used such writing tools to assist with writing the manuscript this must be	No

declared and cited in the text. Authors should not list AI-writing tools and other AI-assisted technologies as an author or co-author and should acknowledge that they are fully responsible for text generated or refined by AI-writing tools.<br><br>A summary of use (particularly in the introduction or among methods) needs to be included at the end of the paper, and the outputs should also be included as a supplementary file hosted in GigaDB or other open repositories. Please read our guidelines for more information. <br>By submitting to GigaScience, you are aware of the journal's AI-writing tools policy, and if you have declared use of such tools below, you have acknowledged this where appropriate in your manuscript and have made a summary of use and outputs available. <br><br>**AI-assisted writing tools have been used in the preparation of this manuscript?<br>** 	

Manuscript cleaned
Click here to access/download;Manuscript;R_Revised Manuscript NN edits_EL_CLEAN_NN.docx

Title:

A Case for Estradiol: Younger Brains in Women with Earlier Menarche and Later Menopause

Authors and Affiliations:

Eileen Luders[1,2,3,4*] | Inger Sundström Poromaa[1] | Claudia Barth[5] | Christian Gaser[6,7,8]

[1]Department of Women's and Children's Health, Uppsala University, Uppsala, Sweden
[2]Swedish Collegium for Advanced Study (SCAS), Uppsala, Sweden
[3]School of Psychology, University of Auckland, Auckland, New Zealand
[4]Laboratory of Neuro Imaging, School of Medicine, University of Southern California, Los Angeles, USA
[5]Department of Psychiatric Research, Diakonhjemmet Hospital, Oslo, Norway
[6]Department of Psychiatry and Psychotherapy, Jena University Hospital, Jena, Germany
[7]Department of Neurology, Jena University Hospital, Jena, Germany
[8]German Center for Mental Health (DZPG)

**Correspondence should be addressed to:*

Eileen Luders, Ph.D.

Department of Women's and Children's Health, Uppsala University, Uppsala, Sweden
Email: eileen.lueders@uu.se

Number of Words in Abstract:	**≤250**
Number of Figures:	**3**
Number of Tables:	**3**
Number of Pages:	**26**
Supplemental Material:	**3 Tables**

Date of Submission:	**September 27th, 2024**
Date of Revision 1:	**April 8, 2025**
Date of Revision 2:	**May 4, 2025**

Abstract

The transition to menopause is marked by a gradual decrease of estradiol. Concurrently, the risk of dementia in women increases around menopause, suggesting that estradiol (or the lack thereof) plays a role in the development of dementia and other age-related neuropathologies. Here, we set out to investigate whether there is a link between brain aging and estradiol-associated events, such as menarche and menopause. For this purpose, we applied a well-validated machine learning approach to analyze both cross-sectional and longitudinal data from a sample of 1,006 postmenopausal women who underwent structural magnetic resonance imaging (MRI) twice, approximately two years apart. We observed less brain aging in women with an earlier menarche, a later menopause, and a longer reproductive span (i.e., the time interval between menarche and menopause). These effects were evident both cross-sectionally and longitudinally, supporting the notion that estradiol has neuroprotective properties and contributes to brain preservation. However, further research is required because the observed effects were small, estradiol was not directly measured, and other factors may modulate female brain health. Future studies might benefit from incorporating actual estradiol (and other hormone) measures, as well as considering genetic predispositions and lifestyle factors alongside indicators of brain aging to deepen our understanding of estradiol's role in maintaining brain health. Additionally, including more diverse study populations (e.g., varying in ethnicity, socioeconomic status, and health status) in follow-up research would enhance the generalizability and applicability of these findings.

Keywords:

brain age; estradiol; machine learning; menarche; MRI; menopause; structural neuroimaging

Background

Estradiol is the most potent and prevalent form of estrogen during the reproductive life of a woman [1]. Generally speaking, estradiol levels start increasing just before the first menstrual period (menarche) and then plateau on a high level until they start decreasing during perimenopause. After the final menstrual period (i.e., menopause) estradiol levels decrease further and eventually reach plateauing low levels during postmenopause [2]. The risk for dementia in women is known to increase around menopause [3-6] and thus it stands to reason that estradiol plays a role for the development of dementia and other age-related neuropathologies. Indeed, studies using animal models have demonstrated that estradiol promotes synaptic plasticity, enhances neurogenesis, and protects against oxidative stress and neuroinflammation [7-13]—mechanisms that are critical for maintaining brain health and mitigating age-related brain degeneration. While extensive research has also been conducted in humans, focusing on specific phases (e.g., menarche, pregnancy, menopause) or interventions (e.g., hormonal contraceptives, menopausal estrogen therapy and anti-estrogen therapy), definitive evidence for the neuroprotective role of estradiol remains elusive [4, 14-29]. Specifically, in the context of menarche and menopause, both early and late onset have been associated with an increased risk of dementia as well as with markers of brain aging and cognitive function [4, 14-21, 24-26].

To further advance this field of research, the current study set out to determine if there is a link between a woman's estimated brain age (a biological marker of brain health [30]) and the reproductive span (i.e., the interval between menarche and menopause when estradiol levels are high). If a lack of estradiol is among the driving factors for diminished brain health later in

life, brain age and reproductive span should be inversely related (negative correlation). To be able to relate our findings to others in the literature [14-16] and to provide a frame of reference for future studies, we additionally investigated if there is a significant link between estimated brain age and the age at menarche as well as the age at menopause. Assuming a neuroprotective effect of estradiol, we expected that a lower brain age would be linked to an earlier menarche (positive correlation) and to a later menopause (negative correlation). Importantly, our study comprises both cross-sectional and longitudinal components, with follow-up data acquired approximately two years after the initial brain scan.

To estimate brain age, we used structural brain images and a well-validated high-dimensional pattern recognition approach, as detailed elsewhere . Briefly, the difference between the estimated brain age and the chronological age yields a so-called brain age gap estimate (BrainAGE) in years. The BrainAGE index is negative if a brain is estimated younger than its chronological age; it is positive if a brain is estimated older than its chronological age. For example, a 50-year-old woman with a BrainAGE index of -3 years shows the aging pattern of a 47-year-old. The BrainAGE algorithm has been shown to be robust and reliable across datasets, age-ranges, and scanner types [31, 33]; it has been successfully applied in a wide range of studies [31, 32, 34-36] including those capturing hormonal changes in women [37, 38]. Moreover, the BrainAGE index has been demonstrated to work as a predictor of dementia as well as age-related cognitive decline [34, 39]. A major advantage of the BrainAGE approach is its ability to aggregate complex, spatially distributed age-related changes in brain structure into a single, interpretable biomarker. Such brain age metrics provide a powerful way to study the influence of biological

factors across the female lifespan, including the effects of cumulative estrogen exposure and genetic risk for age-related brain degeneration, as also shown in other studies .

Materials and Methods

Data Description

The study is based on a carefully selected sample of 1,006 postmenopausal women from the UK Biobank [40] which was accessed under application number #41655. The UK Biobank is a biomedical database and research resource that contains genetic, lifestyle and health information from half a million people. In the UK Biobank cohort, 94.6% of participants are of white ethnicity [41]. For general ethnic information, see [42]; for ethnic information on all women with available longitudinal data, see **Supplemental Table 3**. The UK Biobank holds the ethical approval from the North West Multi-Centre Research Ethics Committee (MREC) and is in possession of the informed consents. Written informed consent was obtained from all participants. Inclusion criteria for the current study were women with available longitudinal data as well as information on age at menarche and age at menopause. Exclusion criteria for the current study were pre-existing neurological or psychiatric diagnoses as per UK Biobank data fields #41202-0.0 to #41202-0.78. In addition, to further increase the homogeneity of the sample, we excluded women whose age at menarche was younger than 10 or older than 18, or whose age at menopause was younger than 45 or older than 60. This resulted in a final sample size of 1,006 women. **Table 1** provides information on this final sample; **Figure 1** summarizes the steps related to the sample selection. For each woman, one initial brain scan and one follow-up brain scan – approximately two years apart (mean ± SD: 2.35 ± 6.12 years) – were obtained *after*

menopause. These T1-weighted brain images were acquired on a 3 Tesla Siemens Skyra scanner using a 32-channel head coil, as described elsewhere , [44].

– Table 1 –

– Figure 1 –

Data Analyses

Using the aforementioned T1-weighted images, we applied a number of processing routines implemented in the CAT12 toolbox [45] (version 12.8), which resulted in bias-corrected, spatially normalized, and tissue-classified brain images, as detailed elsewhere [31, 38]. The normalized gray and white matter partitions were smoothed using a 4 and 8 mm full-width-at-half-maximum (FWHM) Gaussian kernel, and image resolution was set to 4 and 8 mm. For further data reduction, we applied a principal component analysis (PCA) using singular value decomposition to all the models using n–1 PCA components (n = minimum of voxel number or sample size). Prior to applying the PCA, the data were normalized by scaling the values between 0 and 1 and subtracting the mean. The transformation matrix derived from the training data PCA was used to project the normalized test data onto this principal component space.

For the estimation of the BrainAGE index, we employed a Gaussian Process Regression (GPR) [46] that uses a linear covariance function, a constant mean function, and a Gaussian likelihood function. Hyperparameters were set to 100 for the constant mean function and to -1 for the likelihood function based on prior exploratory analyses [33]. As training data, we selected 3,046 individuals from the UK Biobank where two time points were available. To avoid overfitting

and ensure generalizability, we employed 10-fold cross-validation separately for the initial and follow-up brain scan, where the dataset was randomly partitioned into 10 equally sized subsets. In each iteration, one-fold was used as the test set, and the remaining nine as the training set. This process was repeated 10 times, and performance metrics (e.g., MAE) were averaged across folds. To estimate the individual brain ages, eight models based on the aforementioned sets of images (i.e., gray matter/white matter, 4 mm/8 mm Gaussian kernel, and 4 mm/8 mm image resolution) were combined using a general linear model where the weights of the models were derived by maximizing the variance to the parameter of interest (e.g., menopause). The difference between the resulting estimated brain age and the chronological age was then calculated as the BrainAGE index (in years).

Statistical Analyses

Main Analyses

After computing the BrainAGE index for all 1,006 women at initial and follow-up scan, we first removed the linear age trend that is typically seen in BrainAGE estimation. Then, we conducted two analysis streams using linear regressions in MATLAB (version R2023b; RRID:SCR_001622), one cross-sectional and one longitudinal. For all analyses, alpha was set at 0.05 (two-tailed).

For the cross-sectional stream, we tested if there is a significant link between the BrainAGE index at the initial brain scan and the reproductive span. In addition, we tested if there is a significant link between the BrainAGE index at the initial brain scan and the age at menarche as well as the age at menopause. For the longitudinal stream, we first subtracted the BrainAGE index at the initial brain scan from the BrainAGE index at the follow-up brain scan, which resulted

in a Δ BrainAGE index for each individual. This method, often referred to as "change score" analysis, produces statistical results that are comparable to those resulting from a repeated-measures ANOVA with two time points. Using the Δ BrainAGE index, we then tested for significant links with the reproductive span, the age at menarche, and the age at menopause.

Sensitivity Analyses

The aforementioned main analyses were repeated while accounting for potential confounds known to affect brain health. More specifically, we removed the variance associated with the number of live births [47] (UK Biobank data field #2734), hormone replacement therapy [14] (#2814), hysterectomy [48] (#3591), bilateral oophorectomy [48] (#834), body mass index [49] (#21001), diastolic and systolic blood pressure [50] (#4079 and #4080), diabetes [51] (#2443), education [52] (#6138), income [53] (#738), and a composite lifestyle factor [54]. The latter was expressed as a general lifestyle score that was calculated based on a number of factors (see **Supplemental Table 1**), known to increase / decrease the risk of adverse cardiovascular events. Since not all women had information on all potential confounds (see **Supplemental Table 2**), we applied an imputation method using the MATLAB function 'fillmissing'. That is, missing entries were replaced with the corresponding values from the nearest neighbor rows, calculated based on the pairwise Euclidean distance between rows. Imputation was applied to up to 295 women, depending on the potential confound. For the cross-sectional stream, we tested if there is a significant link between the BrainAGE index at the initial brain scan and the reproductive span (age at menarche and age at menopause, respectively). Likewise, for the longitudinal stream, we

tested if there is a significant link between the Δ BrainAGE index and the reproductive span (age at menarche and age at menopause, respectively).

Results

Main Analyses

As shown in **Figure 2** (left), our cross-sectional analyses revealed a significant negative association between BrainAGE and the reproductive span. In other words, brains of women with longer reproductive spans were estimated younger than brains of women with shorter reproductive spans. As also shown in **Figure 2** (right), there was a significant positive association between BrainAGE and age at menarche (i.e., the earlier the menarche, the younger the brain) and a significant negative association between BrainAGE and age at menopause (i.e., the later the menopause, the younger the brain). As shown in **Table 2** (main analyses), effect sizes were small [55], with r-values at -0.11, 0.14, and -0.09 for reproductive span, menarche, and menopause, respectively). The slopes of the regression indicate different rates of change for menarche and menopause (0.32 and -0.10, respectively). More specifically, for each year younger at menarche, brains are estimated 0.32 years younger (which corresponds to 3.2 years younger for each 10 years). In contrast, for each year older at menopause, brains are estimated 0.1 year younger (which corresponds to 1 year younger for each 10 years).

– Figure 2 –

– Table 2 –

As shown in **Figure 3** and **Table 3** (main analyses), our longitudinal findings confirm the observed cross-sectional relationships. More specifically, Δ BrainAGE was negatively linked to reproductive span and menopause, and positively linked to age at menarche. All associations were significant. Again, effect sizes were small, with r-values at -0.12, 0.06, and -0.12 for reproductive span, menarche, and menopause, respectively. The slopes of the regression are still somewhat different for menarche and menopause (0.08 and -0.06, respectively), albeit more similar than in the cross-sectional analysis: For each year younger at menarche, brains are estimated 0.08 years younger (0.8 years over 10 years), whereas for each year older at menopause, brains are estimated 0.06 years younger (0.6 years over 10 years).

– Figure 3 –

– Table 3 –

Sensitivity Analyses

The results described above remained comparable when removing the variance associated with the number of live births, hormone replacement therapy, hysterectomy, bilateral oophorectomy, body mass index, diastolic and systolic blood pressure, diabetes, education, income, and a composite lifestyle factor. In other words, when examining the association between BrainAGE and reproductive span, we observed a negative association. Likewise, there was a positive association between BrainAGE and age at menarche and a negative association between

BrainAGE and age at menopause. The effects were significant for reproductive span, menarche, and menopause for the cross-sectional analyses (see **Table 2**; Sensitivity Analyses), and for reproductive span and menopause for the longitudinal (see **Table 3**; Sensitivity Analyses).

Discussion

Here we assessed links between estimated brain age and milestones in a woman's reproductive life in a well-powered sample of more than a thousand postmenopausal women. We detected less brain aging in women with longer reproductive spans, earlier menarche, and later menopause (see **Figures 2-3** and **Tables 2-3**).

Correspondence with Previous Findings

Our findings are in line with the outcomes of other studies suggesting a longer reproductive span , an earlier menarche [20, 21], as well as a later menopause to be associated with a lower risk of developing dementia or better retained cognitive function. Furthermore, given that the BrainAGE index is based on the weighted distribution of gray and whiter matter tissue in the brain, our findings are also in agreement with reports of lower brain volumes as well as higher rates of brain tissue loss during menopause compared to premenopause or in postmenopausal women compared to premenopausal women [56-58]. In addition, our findings agree with observed effects across the menstrual cycle linking high estradiol levels at ovulation to lower BrainAGE estimates [37]. Altogether, the outcomes of our study seem to suggest that estradiol contributes to brain health, which is in agreement with other studies reporting positive effects of estradiol

on brain health and cognition within the framework of aging and/or menopausal hormone therapy [59-63].

Menarche versus Menopause

The outcomes of the main analyses indicate that both, an earlier menarche and a later menopause, are significantly associated with less brain aging. However, menarche and menopause differ with respect to the strength of their relationship with age (which is reflected in the correlation coefficient) and their rate of change with age (which is reflected in the slope of the regression line). This might indicate somewhat different underlying biological mechanisms and/or confounds for menarche and menopause. For example, during menopause, in addition to decreasing levels of estradiol, increasing levels of follicle-stimulating hormones may cause an accelerated deposition of amyloid-β and Tau [64], which enhances brain atrophy. Moreover, menopause is marked by disadvantageous alterations in cytokine and T cell profiles [65] , which are linked to an enhanced inflammation. Alternatively, the less strong link pertaining to menarche could also reflect the fact that, later in life, it is probably more challenging to accurately remember the onset of menarche than the onset of menopause.

Potential Implications

Given that estradiol levels start decreasing during perimenopause and further decrease after menopause, our findings may explain why the risk for dementia in women is known to increase around menopause [3-6] and why there is an increased age-independent prevalence of Alzheimer's disease in women compared to men [63]. Moreover, our findings seem to support

to the concept of the “window of opportunity”, spanning the years leading up to menopause to the years immediately after menopause, where health interventions (e.g., menopausal hormone treatment) may combat the increased risk for Alzheimer's disease in some women [5, 66-68]. In fact, several large-scale projects have investigated the effects of menopausal hormone treatment on cognitive function and Alzheimer's risk, but results are inconclusive (potentially relevant modulators of treatment outcomes are discussed elsewhere [59, 69-74]).

The current findings seem to suggest a protective effect of estradiol and as such seem promising in the framework of prevention and intervention. However, further research is required, as the effect sizes for the observed associations were small (albeit smaller effect sizes are not uncommon in studies with larger sample sizes) and various factors, such as genetics, life style, or hormones other than estradiol, could play a greater (or at least an additional) role in preserving brain health [2, 76, 77]. Moreover, our study did not measure estradiol directly, and links between estradiol and brain aging seem to be rather complex as indicated by the outcomes of other studies. For example, it was reported that, compared to no exposure or no dose, exposure to low concentrations of estradiol or low doses of estrogen enhanced neuronal survival and increased anti-inflammatory markers (i.e., positive links), while exposure to high concentrations of estradiol as well as high doses of estrogen had the opposite effect (i.e., negative links) [27, 28]. Another study reported U-shaped curves suggesting that both early and late menarche are associated with an increased risk for dementia (i.e., positive and negative links) [15]. And yet another study reported either negative links or missing links between age at menarche and brain aging depending on the potential confounds accounted for [14]. Interestingly, this latter study also reported that, in carriers of the apolipoprotein E type 4 allele

(APOE e4), higher levels of estradiol at menopause were associated with increased brain aging (positive link). In contrast, in non-carriers, higher levels of estradiol at menopause were associated with decreased brain aging (negative link) [14].

Conclusion

Our study revealed less brain aging in women with a larger reproductive span, earlier menarche, and later menopause. Thus, sex hormones—potentially estradiol—may contribute to brain health. However, follow-up research is required because the effects observed in the current study were small, estradiol was not directly examined, and female brain health is likely modulated by factors other than estradiol. Future studies might benefit from incorporating actual estradiol (and other hormone) measurements, as well as considering genetic predispositions and lifestyle factors alongside structural brain measures. Moreover, to build a more comprehensive understanding and expand this understudied field, future research focusing on specific timeframes surrounding menopause—such as perimenopause (i.e., the time preceding the final menstrual period) or early postmenopause (e.g., the initial year after menopause) versus late menopause (e.g., ten years after menopause)—would be valuable. Lastly, the UK Biobank (i.e., the source of the current sample) is biased towards healthy and more socioeconomically privileged individuals with a predominant white ethnic background [41]. Thus, conducting research in more diverse populations, including individuals from different ethnic, socioeconomic, and health backgrounds, would improve the generalizability of findings and provide a broader understanding of the relationship between estradiol and brain health.

Availability of Source Code and Requirements

Project name: BrainAGE-UKBiobank

Project home page: https://github.com/ChristianGaser/BrainAGE-UKBiobank

Operating system(s): Platform independent

Programming language: MATLAB

Other requirements: SPM12, CAT12, BrainAGE

License: GNU GPL-3.0

Data Availability

The individual-level proxy measures obtained from the prediction models in this work will be shared after publication in agreement with UK Biobank regulations. Snapshots of our GitHub is in Software Heritage [78], and the DOME-ML annotations for this work can be found in the DOME Registry [79]. The input data are available to other researchers through the UK Biobank's controlled access scheme . The procedure to apply for access [80] requires registering with the UK Biobank and completion of an application form detailing:

- A summary of the planned research
- The UK Biobank data fields required for the project
- A description of derivatives (data, variables) generated by the project

Abbreviations

BrainAGE = brain age gap estimate
MRI = magnetic resonance imaging
PCA = principal component analysis

Declarations

Consent for publication

The data of the UK Biobank were accessed under application number #41655. The article was composed using the STROBE cohort checklist [81].

Competing Interests

The authors declare that the research was conducted in the absence of any commercial or financial relationships that could be construed as a potential conflict of interest.

Funding

CB received funding from the South-Eastern Norway Regional Health Authority (2023037, 2022103).

Authors' contributions

Conceptualization: E.L., C.G.; software, methodology, visualization, and formal analysis: C.G.; writing—original draft: E.L., C.G.; writing—revision: E.L., C.B., I.S., C.G.; funding acquisition: C.B.

Acknowledgments

EL was supported by the Swedish Collegium for Advanced Study (SCAS) and Erling-Persson Family Foundation.

References

1. Thomas MP and Potter BV. The structural biology of oestrogen metabolism. The Journal of steroid biochemistry and molecular biology. 2013;137:27-49. doi:10.1016/j.jsbmb.2012.12.014.
2. Barth C, Crestol A, de Lange AG and Galea LAM. Sex steroids and the female brain across the lifespan: insights into risk of depression and Alzheimer's disease. Lancet Diabetes Endocrinol. 2023;11 12:926-41. doi:10.1016/S2213-8587(23)00224-3.
3. Farrer LA, Cupples LA, Haines JL, Hyman B, Kukull WA, Mayeux R, et al. Effects of age, sex, and ethnicity on the association between apolipoprotein E genotype and Alzheimer disease. A meta-analysis. APOE and Alzheimer Disease Meta Analysis Consortium. JAMA. 1997;278 16:1349-56.
4. Brinton RD, Yao J, Yin F, Mack WJ and Cadenas E. Perimenopause as a neurological transition state. Nat Rev Endocrinol. 2015;11 7:393-405. doi:10.1038/nrendo.2015.82.
5. Mosconi L, Rahman A, Diaz I, Wu X, Scheyer O, Hristov HW, et al. Increased Alzheimer's risk during the menopause transition: A 3-year longitudinal brain imaging study. PLoS One. 2018;13 12:e0207885. doi:10.1371/journal.pone.0207885.
6. Rahman A, Schelbaum E, Hoffman K, Diaz I, Hristov H, Andrews R, et al. Sex-driven modifiers of Alzheimer risk: A multimodality brain imaging study. Neurology. 2020;95 2:e166-e78. doi:10.1212/WNL.0000000000009781.
7. Vegeto E, Benedusi V and Maggi A. Estrogen anti-inflammatory activity in brain: a therapeutic opportunity for menopause and neurodegenerative diseases. Front Neuroendocrinol. 2008;29 4:507-19. doi:10.1016/j.yfrne.2008.04.001.
8. Behl C, Widmann M, Trapp T and Holsboer F. 17-beta estradiol protects neurons from oxidative stress-induced cell death in vitro. Biochem Biophys Res Commun. 1995;216 2:473-82. doi:10.1006/bbrc.1995.2647.
9. Barha CK, Lieblich SE and Galea LA. Different forms of oestrogen rapidly upregulate cell proliferation in the dentate gyrus of adult female rats. J Neuroendocrinol. 2009;21 3:155-66. doi:10.1111/j.1365-2826.2008.01809.x.
10. Tanapat P, Hastings NB, Reeves AJ and Gould E. Estrogen stimulates a transient increase in the number of new neurons in the dentate gyrus of the adult female rat. The Journal of neuroscience : the official journal of the Society for Neuroscience. 1999;19 14:5792-801. doi:10.1523/JNEUROSCI.19-14-05792.1999.
11. Inagaki T, Kaneko N, Zukin RS, Castillo PE and Etgen AM. Estradiol attenuates ischemia-induced death of hippocampal neurons and enhances synaptic transmission in aged, long-term hormone-deprived female rats. PLoS One. 2012;7 6:e38018. doi:10.1371/journal.pone.0038018.
12. Woolley CS and McEwen BS. Estradiol mediates fluctuation in hippocampal synapse density during the estrous cycle in the adult rat. The Journal of neuroscience : the official journal of the Society for Neuroscience. 1992;12 7:2549-54. doi:10.1523/JNEUROSCI.12-07-02549.1992.
13. Yankova M, Hart SA and Woolley CS. Estrogen increases synaptic connectivity between single presynaptic inputs and multiple postsynaptic CA1 pyramidal cells: a serial electron-microscopic study. ProcNatlAcadSciUSA. 2001;98 6:3525-30.
14. de Lange AG, Barth C, Kaufmann T, Maximov, II, van der Meer D, Agartz I, et al. Women's brain aging: Effects of sex-hormone exposure, pregnancies, and genetic risk for Alzheimer's disease. Hum Brain Mapp. 2020;41 18:5141-50. doi:10.1002/hbm.25180.
15. Gong J, Harris K, Peters SAE and Woodward M. Reproductive factors and the risk of incident dementia: A cohort study of UK Biobank participants. PLoS Med. 2022;19 4:e1003955. doi:10.1371/journal.pmed.1003955.

16. Jani M, Zackova L, Piler P, Andryskova L, Brazdil M and Mareckova K. Birth outcomes, puberty onset, and obesity as long-term predictors of biological aging in young adulthood. Front Nutr. 2022;9:1100237. doi:10.3389/fnut.2022.1100237.
17. Lindseth LRS, de Lange AG, van der Meer D, Agartz I, Westlye LT, Tamnes CK, et al. Associations between reproductive history, hormone use, APOE epsilon4 genotype and cognition in middle- to older-aged women from the UK Biobank. Front Aging Neurosci. 2022;14:1014605. doi:10.3389/fnagi.2022.1014605.
18. Kuh D, Cooper R, Moore A, Richards M and Hardy R. Age at menopause and lifetime cognition: Findings from a British birth cohort study. Neurology. 2018;90 19:e1673-e81. doi:10.1212/WNL.0000000000005486.
19. McLay RN, Maki PM and Lyketsos CG. Nulliparity and late menopause are associated with decreased cognitive decline. J Neuropsychiatry Clin Neurosci. 2003;15 2:161-7. doi:10.1176/jnp.15.2.161.
20. Karim R, Dang H, Henderson VW, Hodis HN, St John J, Brinton RD, et al. Effect of Reproductive History and Exogenous Hormone Use on Cognitive Function in Mid- and Late Life. J Am Geriatr Soc. 2016;64 12:2448-56. doi:10.1111/jgs.14658.
21. Ryan J, Carriere I, Scali J, Ritchie K and Ancelin ML. Life-time estrogen exposure and cognitive functioning in later life. Psychoneuroendocrinology. 2009;34 2:287-98. doi:10.1016/j.psyneuen.2008.09.008.
22. Branigan GL, Soto M, Neumayer L, Rodgers K and Brinton RD. Association Between Hormone-Modulating Breast Cancer Therapies and Incidence of Neurodegenerative Outcomes for Women With Breast Cancer. JAMA Netw Open. 2020;3 3:e201541. doi:10.1001/jamanetworkopen.2020.1541.
23. Comasco E, Frokjaer VG and Sundstrom-Poromaa I. Functional and molecular neuroimaging of menopause and hormone replacement therapy. Frontiers in neuroscience. 2014;8:388. doi:10.3389/fnins.2014.00388.
24. Georgakis MK, Kalogirou EI, Diamantaras AA, Daskalopoulou SS, Munro CA, Lyketsos CG, et al. Age at menopause and duration of reproductive period in association with dementia and cognitive function: A systematic review and meta-analysis. Psychoneuroendocrinology. 2016;73:224-43. doi:10.1016/j.psyneuen.2016.08.003.
25. Ambikairajah A, Tabatabaei-Jafari H, Hornberger M and Cherbuin N. Age, menstruation history, and the brain. Menopause. 2020;28 2:167-74. doi:10.1097/GME.0000000000001688.
26. Gilsanz P, Lee C, Corrada MM, Kawas CH, Quesenberry CP, Jr. and Whitmer RA. Reproductive period and risk of dementia in a diverse cohort of health care members. Neurology. 2019;92 17:e2005-e14. doi:10.1212/WNL.0000000000007326.
27. Prestwood KM, Unson C, Kulldorff M and Cushman M. The effect of different doses of micronized 17beta-estradiol on C-reactive protein, interleukin-6, and lipids in older women. J Gerontol A Biol Sci Med Sci. 2004;59 8:827-32. doi:10.1093/gerona/59.8.m827.
28. Chen S, Nilsen J and Brinton RD. Dose and temporal pattern of estrogen exposure determines neuroprotective outcome in hippocampal neurons: therapeutic implications. Endocrinology. 2006;147 11:5303-13. doi:10.1210/en.2006-0495.
29. Luders E, Sundstrom-Poromaa I and Kurth F. The neuroanatomy of menopause. Hormones and Behavior (in press). 2025.
30. Franke K and Gaser C. Ten Years of BrainAGE as a Neuroimaging Biomarker of Brain Aging: What Insights Have We Gained? Front Neurol. 2019;10:789. doi:10.3389/fneur.2019.00789.
31. Franke K, Ziegler G, Kloppel S and Gaser C. Estimating the age of healthy subjects from T1-weighted MRI scans using kernel methods: exploring the influence of various parameters. Neuroimage. 2010;50 3:883-92.

32. Franke K, Luders E, May A, Wilke M and Gaser C. Brain maturation: Predicting individual BrainAGE in children and adolescents using structural MRI. Neuroimage. 2012;63 3:1305-12.
33. Kalc P, Dahnke R, Hoffstaedter F and Gaser C. BrainAGE: Revisited and reframed machine learning workflow. Hum Brain Mapp. 2024;45 3:e26632. doi:10.1002/hbm.26632.
34. Gaser C, Franke K, Kloppel S, Koutsouleris N and Sauer H. BrainAGE in Mild Cognitive Impaired Patients: Predicting the Conversion to Alzheimer's Disease. PLoS One. 2013;8 6:e67346. doi:10.1371/journal.pone.0067346.
35. Franke K, Ristow M and Gaser C. Gender-specific impact of personal health parameters on individual brain aging in cognitively unimpaired elderly subjects. Front Aging Neurosci. 2014;6:94. doi:10.3389/fnagi.2014.00094.
36. Luders E, Cherbuin N and Gaser C. Estimating brain age using high-resolution pattern recognition: Younger brains in long-term meditation practitioners. NeuroImage. 2016;134:508-13. doi:10.1016/j.neuroimage.2016.04.007.
37. Franke K, Hagemann G, Schleussner E and Gaser C. Changes of individual BrainAGE during the course of the menstrual cycle. NeuroImage. 2015;115:1-6. doi:10.1016/j.neuroimage.2015.04.036.
38. Luders E, Gingnell M, Poromaa IS, Engman J, Kurth F and Gaser C. Potential Brain Age Reversal after Pregnancy: Younger Brains at 4-6Weeks Postpartum. Neuroscience. 2018;386:309-14. doi:10.1016/j.neuroscience.2018.07.006.
39. Giannakopoulos P, Montandon ML, Herrmann FR, Hedderich D, Gaser C, Kellner E, et al. Alzheimer resemblance atrophy index, BrainAGE, and normal pressure hydrocephalus score in the prediction of subtle cognitive decline: added value compared to existing MR imaging markers. Eur Radiol. 2022; doi:10.1007/s00330-022-08798-0.
40. The UK Biobank. https://www.ukbiobank.ac.uk/. Accessed 1 May 2025.
41. Fry A, Littlejohns TJ, Sudlow C, Doherty N, Adamska L, Sprosen T, et al. Comparison of Sociodemographic and Health-Related Characteristics of UK Biobank Participants With Those of the General Population. Am J Epidemiol. 2017;186 9:1026-34. doi:10.1093/aje/kwx246.
42. Biobank UK, Datafield 21000. https://biobank.ctsu.ox.ac.uk/crystal/field.cgi?id=21000. Accessed 30 April 2025.
43. Alfaro-Almagro F, Jenkinson M, Bangerter NK, Andersson JLR, Griffanti L, Douaud G, et al. Image processing and Quality Control for the first 10,000 brain imaging datasets from UK Biobank. NeuroImage. 2018;166:400-24. doi:10.1016/j.neuroimage.2017.10.034.
44. UK Biobank protocol for SIEMENS MAGNETOM Skyra syngo MR D13. http://biobank.ctsu.ox.ac.uk/crystal/crystal/docs/bmri_V4_23092014.pdf. Accessed 30 April 2025.
45. Gaser C, Dahnke R, Thompson PM, Kurth F, Luders E and The Alzheimer's Disease Neuroimaging I. CAT: a computational anatomy toolbox for the analysis of structural MRI data. Gigascience. 2024;13 doi:10.1093/gigascience/giae049.
46. Rasmussen CEW, C. K. I. Gaussian Processes for Machine Learning. MIT Press. 2006.
47. de Lange AG, Kaufmann T, van der Meer D, Maglanoc LA, Alnaes D, Moberget T, et al. Population-based neuroimaging reveals traces of childbirth in the maternal brain. Proc Natl Acad Sci U S A. 2019;116 44:22341-6. doi:10.1073/pnas.1910666116.
48. Rocca WA, Grossardt BR, Shuster LT and Stewart EA. Hysterectomy, oophorectomy, estrogen, and the risk of dementia. Neurodegener Dis. 2012;10 1-4:175-8. doi:10.1159/000334764.
49. Tungler A, Van der Auwera S, Wittfeld K, Frenzel S, Terock J, Roder N, et al. Body mass index but not genetic risk is longitudinally associated with altered structural brain parameters. Scientific reports. 2021;11 1:24246. doi:10.1038/s41598-021-03343-3.

50. Cherbuin N, Walsh EI, Shaw M, Luders E, Anstey KJ, Sachdev PS, et al. Optimal Blood Pressure Keeps Our Brains Younger. Front Aging Neurosci. 2021;13:694982. doi:10.3389/fnagi.2021.694982.
51. Antal B, McMahon LP, Sultan SF, Lithen A, Wexler DJ, Dickerson B, et al. Type 2 diabetes mellitus accelerates brain aging and cognitive decline: Complementary findings from UK Biobank and meta-analyses. Elife. 2022;11 doi:10.7554/eLife.73138.
52. Chan MY, Han L, Carreno CA, Zhang Z, Rodriguez RM, LaRose M, et al. Long-term prognosis and educational determinants of brain network decline in older adult individuals. Nat Aging. 2021;1 11:1053-67. doi:10.1038/s43587-021-00125-4.
53. Busby N, Newman-Norlund S, Sayers S, Newman-Norlund R, Wilmskoetter J, Rorden C, et al. Lower socioeconomic status is associated with premature brain aging. Neurobiology of aging. 2023;130:135-40. doi:10.1016/j.neurobiolaging.2023.06.012.
54. Foster HME, Celis-Morales CA, Nicholl BI, Petermann-Rocha F, Pell JP, Gill JMR, et al. The effect of socioeconomic deprivation on the association between an extended measurement of unhealthy lifestyle factors and health outcomes: a prospective analysis of the UK Biobank cohort. Lancet Public Health. 2018;3 12:e576-e85. doi:10.1016/S2468-2667(18)30200-7.
55. Cohen J. A power primer. PsycholBull. 1992;112 1:155-9.
56. Goto M, Abe O, Miyati T, Inano S, Hayashi N, Aoki S, et al. Accelerated hippocampal volume reduction in post-menopausal women: an additional study with Atlas-based method. Radiol Phys Technol. 2011;4 2:185-8. doi:10.1007/s12194-011-0120-7.
57. Goto M, Abe O, Miyati T, Inano S, Hayashi N, Aoki S, et al. 3 Tesla MRI detects accelerated hippocampal volume reduction in postmenopausal women. J Magn Reson Imaging. 2011;33 1:48-53. doi:10.1002/jmri.22328.
58. Lu W, Guo W, Hou K, Zhao H, Shi L, Dong K, et al. Grey matter differences associated with age and sex hormone levels between premenopausal and perimenopausal women: A voxel-based morphometry study. J Neuroendocrinol. 2018;30 12:e12655. doi:10.1111/jne.12655.
59. Manly JJ, Merchant CA, Jacobs DM, Small SA, Bell K, Ferin M, et al. Endogenous estrogen levels and Alzheimer's disease among postmenopausal women. Neurology. 2000;54 4:833-7. doi:10.1212/wnl.54.4.833.
60. Boccardi M, Ghidoni R, Govoni S, Testa C, Benussi L, Bonetti M, et al. Effects of hormone therapy on brain morphology of healthy postmenopausal women: a Voxel-based morphometry study. Menopause. 2006;13 4:584-91. doi:10.1097/01.gme.0000196811.88505.10.
61. Ghidoni R, Boccardi M, Benussi L, Testa C, Villa A, Pievani M, et al. Effects of estrogens on cognition and brain morphology: involvement of the cerebellum. Maturitas. 2006;54 3:222-8. doi:10.1016/j.maturitas.2005.11.002.
62. Kim TH, Kim B, Kim YR, Jeong CW and Lee YH. Gray matter differences associated with menopausal hormone therapy in menopausal women: a DARTEL-based VBM study. Scientific reports. 2023;13 1:1401. doi:10.1038/s41598-023-28673-2.
63. Depypere H, Vergallo A, Lemercier P, Lista S, Benedet A, Ashton N, et al. Menopause hormone therapy significantly alters pathophysiological biomarkers of Alzheimer's disease. Alzheimers Dement. 2023;19 4:1320-30. doi:10.1002/alz.12759.
64. Xiong J, Kang SS, Wang Z, Liu X, Kuo TC, Korkmaz F, et al. FSH blockade improves cognition in mice with Alzheimer's disease. Nature. 2022;603 7901:470-6. doi:10.1038/s41586-022-04463-0.
65. Mishra A and Brinton RD. Inflammation: Bridging Age, Menopause and APOEepsilon4 Genotype to Alzheimer's Disease. Front Aging Neurosci. 2018;10:312. doi:10.3389/fnagi.2018.00312.
66. Scheyer O, Rahman A, Hristov H, Berkowitz C, Isaacson RS, Diaz Brinton R, et al. Female Sex and Alzheimer's Risk: The Menopause Connection. J Prev Alzheimers Dis. 2018;5 4:225-30. doi:10.14283/jpad.2018.34.

67. Davey DA. Alzheimer's disease, dementia, mild cognitive impairment and the menopause: a 'window of opportunity'? Womens Health (Lond). 2013;9 3:279-90. doi:10.2217/whe.13.22.
68. Mishra A, Wang Y, Yin F, Vitali F, Rodgers KE, Soto M, et al. A tale of two systems: Lessons learned from female mid-life aging with implications for Alzheimer's prevention & treatment. Ageing Res Rev. 2022;74:101542. doi:10.1016/j.arr.2021.101542.
69. Middleton LE and Yaffe K. Promising strategies for the prevention of dementia. Arch Neurol. 2009;66 10:1210-5. doi:10.1001/archneurol.2009.201.
70. Marder K and Sano M. Estrogen to treat Alzheimer's disease: too little, too late? So what's a woman to do? Neurology. 2000;54 11:2035-7. doi:10.1212/wnl.54.11.2035.
71. Resnick SM and Henderson VW. Hormone therapy and risk of Alzheimer disease: a critical time. JAMA. 2002;288 17:2170-2. doi:10.1001/jama.288.17.2170.
72. Yaffe K. Estrogens, selective estrogen receptor modulators, and dementia: what is the evidence? Ann N Y Acad Sci. 2001;949:215-22. doi:10.1111/j.1749-6632.2001.tb04024.x.
73. Yaffe K, Haan M, Byers A, Tangen C and Kuller L. Estrogen use, APOE, and cognitive decline: evidence of gene-environment interaction. Neurology. 2000;54 10:1949-54. doi:10.1212/wnl.54.10.1949.
74. Nerattini M, Jett S, Andy C, Carlton C, Zarate C, Boneu C, et al. Systematic review and meta-analysis of the effects of menopause hormone therapy on risk of Alzheimer's disease and dementia. Front Aging Neurosci. 2023;15:1260427. doi:10.3389/fnagi.2023.1260427.
75. Marek S, Tervo-Clemmens B, Calabro FJ, Montez DF, Kay BP, Hatoum AS, et al. Reproducible brain-wide association studies require thousands of individuals. Nature. 2022;603 7902:654-60. doi:10.1038/s41586-022-04492-9.
76. Barth C and de Lange AG. Towards an understanding of women's brain aging: the immunology of pregnancy and menopause. Front Neuroendocrinol. 2020;58:100850. doi:10.1016/j.yfrne.2020.100850.
77. Barth C, Villringer A and Sacher J. Sex hormones affect neurotransmitters and shape the adult female brain during hormonal transition periods. Frontiers in neuroscience. 2015;9 37 doi:10.3389/fnins.2015.00037.
78. Luders E, Sundström Poromaa, Barth C and Gasser G. (2025) A Case for Estradiol: Younger Brains in Women with Earlier Menarche and Later Menopause. (2025) A Case for Estradiol: Younger Brains in Women with Earlier Menarche and Later Menopause. [Computer software]. Software Heritage, https://archive.softwareheritage.org/swh:1:snp:e059c7d873e8e86a633b08c79bfba017ba716549;origin=https://github.com/ChristianGaser/BrainAGE-UKBiobank.
79. Luders E, Sundström Poromaa, Barth C and Gasser G. (2025) A Case for Estradiol: Younger Brains in Women with Earlier Menarche and Later Menopause. [DOME-ML Annotations]. DOME-ML Registry, https://registry.dome-ml.org/review/rpt86qjsd5.
80. UK Biobank Apply for Access. https://www.ukbiobank.ac.uk/enable-your-research/apply-for-access. Accessed 1 May 2025.
81. von Elm E, Altman DG, Egger M, Pocock SJ, Gøtzsche PC, Vandenbroucke JP, et al. The Strengthening the Reporting of Observational Studies in Epidemiology (STROBE) statement: guidelines for reporting observational studies. J Clin Epidemiol. 2008;61 4:344-9. doi:10.1016/j.jclinepi.2007.11.008. PMID: 18313558.

Figure Legends

Figure 1. Flowchart of sample selection.

Figure 2. Correlations with BrainAGE at the initial brain scan. The x-axes show the reproductive span (age, respectively) in years. Of note, age in the UK Biobank has been rounded to the year, so we added a small random jitter to the x-axes to give a better overview about the age distribution. The y-axes show the BrainAGE index in years, with negative values indicating that brains are estimated younger than their chronological age and positive values indicating that brains are estimated older than their chronological age. Panel A displays a negative link between the BrainAGE index and the reproductive span (the longer the reproductive span, the younger the estimated brain age). Panel B displays a positive link between the BrainAGE index and the age at menarche (the earlier the onset of menarche, the younger the estimated brain age). Panel C displays a negative link between the BrainAGE index and the age at menopause (the later the onset of menopause, the younger the estimated brain age). The squares in the density plot represent the individual measures (n=1,006); hot colors indicate a larger overlay of measures; cool colors indicate a smaller overlay. The shaded band is the 95% confidence interval.

Figure 3. Correlations with BrainAGE over 2.35 years (Δ BrainAGE). Panel A displays a negative link between the BrainAGE index and reproductive span (the longer the reproductive span, the smaller the estimated brain age). Panel B displays a positive link between the BrainAGE index and the age at menarche (the earlier the onset of menarche, the younger the estimated brain age). Panel C displays a negative link between the BrainAGE index and the age at menopause (the later the onset of menopause, the younger the estimated brain age). The squares in the density plot represent the individual measures (n=1,006); hot colors indicate a larger overlay of measures; cool colors indicate a smaller overlay. The shaded band is the 95% confidence interval.

Tables

Table 1. Sample characteristics

Variable	Descriptive Statistics
Age at the initial brain scan	mean ± SD: 63.20 ± 6.42 years
Age at the follow-up brain scan	mean ± SD: 65.54 ± 6.37 years
Age at menarche	mean ± SD: 13.02 ± 1.53 years
Age at menopause	mean ± SD: 51.41 ± 3.23 years
Reproductive span	mean ± SD: 38.39 ± 3.55 years
Number of live births	mean ± SD: 1.75 ± 1.16
Number of women with hormone replacement therapy	yes: 306 (30.42%) | no: 700 (69.58%)
Number of women with hysterectomy	yes: 48 (4.77%) | no: 958 (95.23%)
Number of women with bilateral oophorectomy	yes: 37 (3.68%)| no: 969 (96.32%)

SD = standard deviation

Table 2. Associations with BrainAGE at the initial brain scan

	**Main Analyses**					**Sensitivity Analyses***				
	**R^2**	**r**	**p**	**Slope**	**95% Confidence Interval**	**R^2**	**r**	**p**	**Slope**	**95% Confidence Interval**
Reproductive Span	0.01	-0.11	<0.001	-0.11	-0.17 to -0.05	0.01	-0.11	<0.001	-0.11	-0.17 to -0.05
Age at Menarche	0.02	0.14	<0.001	0.32	0.18 to 0.46	0.02	0.14	<0.001	0.33	0.19 to 0.47
Age at Menopause	0.01	-0.09	<0.005	-0.10	-0.17 to -0.03	0.01	-0.09	<0.01	-0.09	-0.16 to -0.03

*While removing the variance associated with the number of live births, hormone replacement therapy, hysterectomy, bilateral oophorectomy, body mass index, diastolic and systolic blood pressure, diabetes, education, income, and a composite lifestyle factor.

Table 3. Associations with changes in BrainAGE over 2.35 years

	Main Analyses					Sensitivity Analyses*				
	R^2	r	p	Slope	95% Confidence Interval	R^2	r	p	Slope	95% Confidence Interval
Reproductive Span	0.01	-0.12	<0.001	-0.06	-0.10 to -0.03	0.01	-0.11	<0.001	-0.06	-0.09 to -0.03
Age at Menarche	<0.01	0.06	<0.05	0.08	0.0 to 0.16	<0.01	0.06	n.s.	0.08	0.0 to 0.16
Age at Menopause	0.01	-0.12	<0.001	-0.07	-0.10 to -0.03	0.01	-0.12	<0.001	-0.07	-0.11 to -0.03

*While removing the variance associated with the number of live births, hormone replacement therapy, hysterectomy, bilateral oophorectomy, body mass index, diastolic and systolic blood pressure, diabetes, education, income, and a composite lifestyle factor.
n.s. = not significant

Click here to access/download;Figure;Figure 1.png

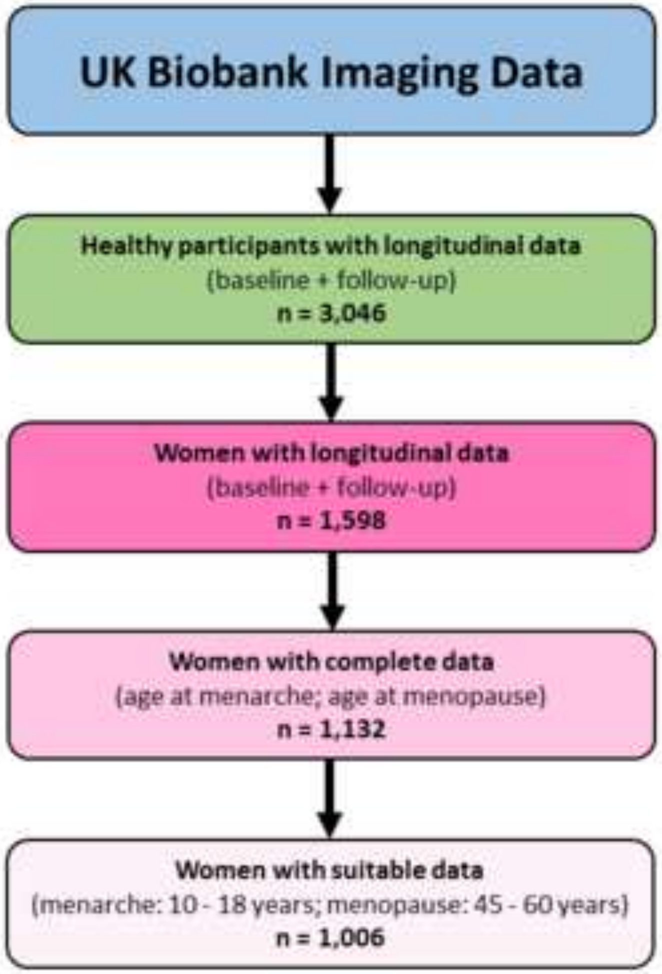

Figure 2

Click here to access/download;Figure;Figure 2.png

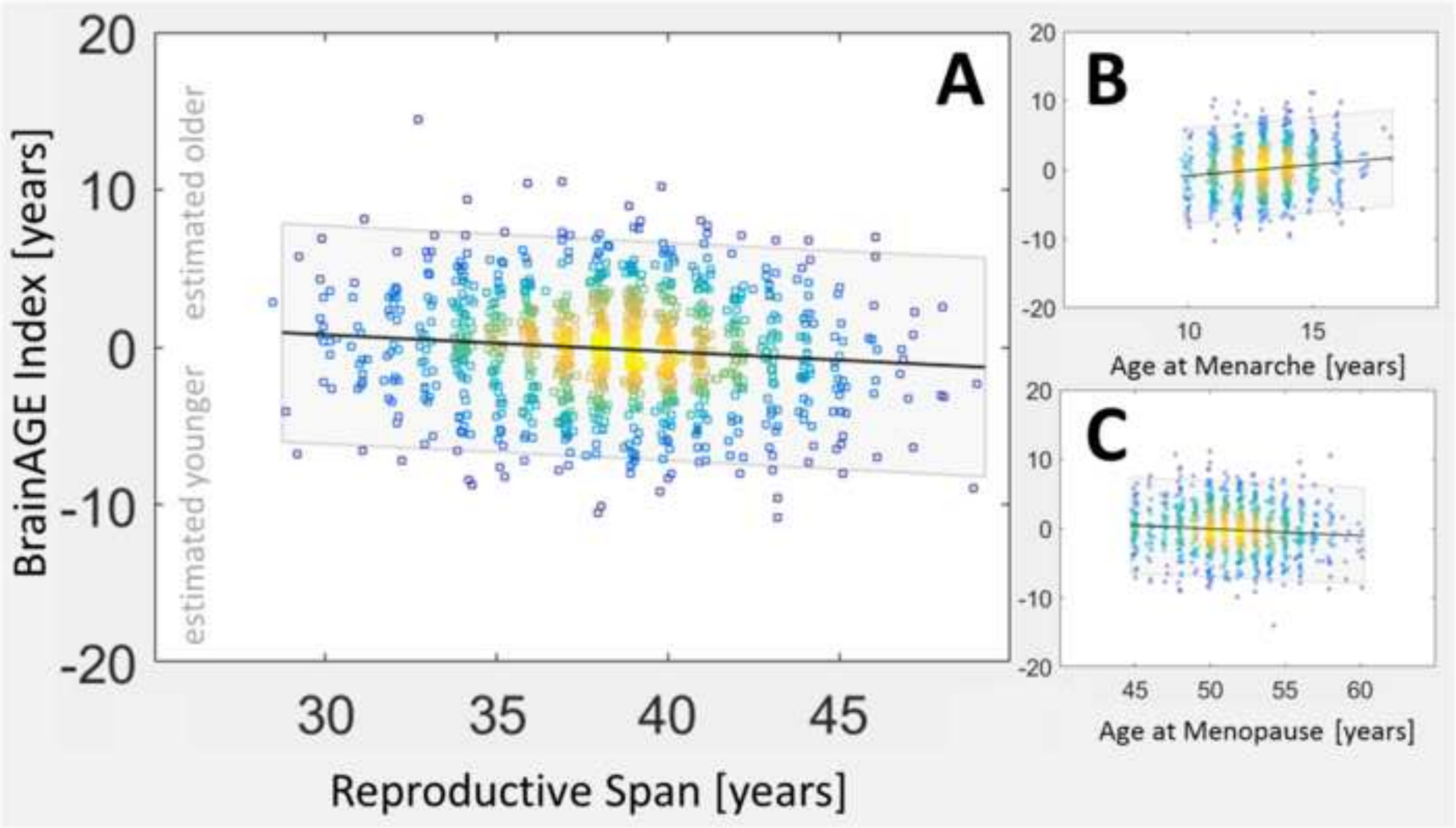

Figure 3

Click here to access/download;Figure;Figure 3.png

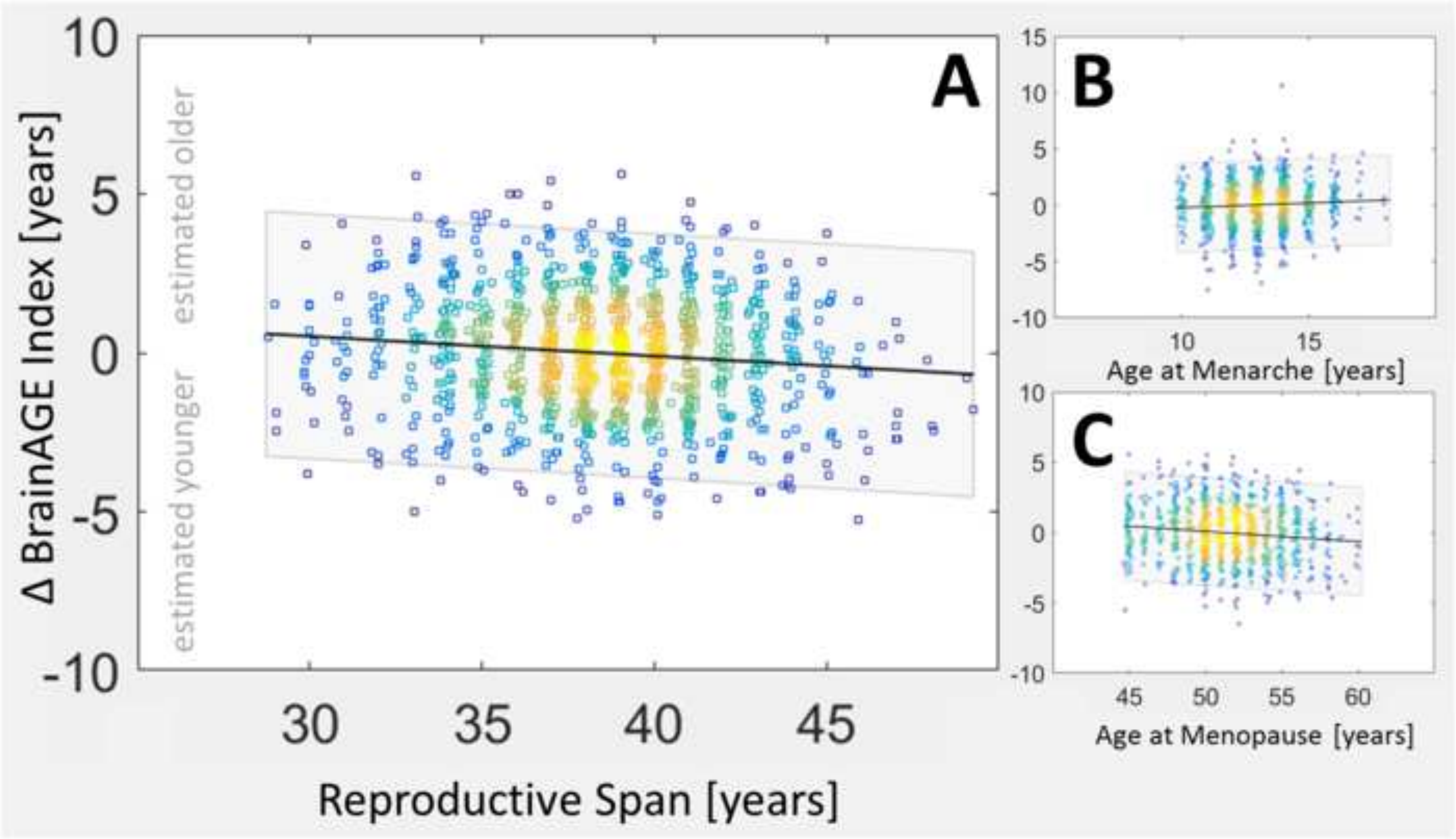

Supplementary Tables

Click here to access/download
Supplementary Material
R_Supplemental Tables.docx
