# Supplementary material for: A Case for estradiol: younger brains in women with earlier menarche and later menopause
Source: Gigascience. 2025 May 23;14:giaf060. doi: 10.1093/gigascience/giaf060 (PMC12099614; doi:10.1093/gigascience/giaf060)
Supplement: giaf060_Reviewer_1_Report_original_submission — Leonardo Bertolin Furstenau -- 12/15/2024 [file giaf060_reviewer_1_report_original_submission.pdf]

Reviewer Report

Title: A Case for Estradiol: Younger Brains in Women with Earlier Menarche and Later Menopause

Version: Original Submission **Date:** 12/15/2024

Reviewer name: Leonardo Bertolin Furstenau

Reviewer Comments to Author:

Overall Rating
This study explored the link between brain aging in women and estradiol-associated events, including menarche and menopause. Results show that estradiol may play a role in women's brain health. However, follow-up research is required because the effects in the current study were small. Specifically, estradiol was not directly explored as a central variable, which must be highlighted and validated in future studies. Nevertheless, the authors performed reliable research, and the data is open for access and may be replicable. This study should be accepted by GigaScience with major reviews. However, the authors still need to put effort into showing the main contributions and the literature gaps they fill with this piece of evidence and better explaining methodological procedures to support replicability. Besides, since the sample was conducted with most British women, caution should be taken regarding its generalization. Data collection was clearly conducted and curated, but the authors must explain further several points regarding methodological procedures, especially using the machine learning technique (e.g., hyperparameters, cross-validation, training and validation). I have conducted studies using technologies in healthcare systems and consultancy projects, but I lack expertise in the field of women's brain aging and reproductive areas. Thus, I strongly recommend another reviewer with expertise in this specific field to assess more nuances regarding brain aging. Below are my recommendations for improvements and points to be clarified.
i) The abstract is well-written, but some improvements could be made. For example, the authors mention dementia and other related diseases in the context but do not emphasize brain aging; this seems disconnected from the study's objective. Also, if the research problem is unclear, consider pointing out research gaps in the literature or limitations of related work. This will help justify this research. In fact, the context should also be used to indicate previous studies using machine learning to identify similar relationships in order to justify the use of such techniques. Besides, more information should be provided about the methodological procedures used, especially those related to the machine learning technique used and the type of algorithm/framework (Gaussian Process Regression). The research strategy should move place (before presenting the results). The results should be more specific, e.g., "We observed less brain aging in women…" how much? The discussion should be rewritten to the importance of this study; currently, it does not sound positive. In this perspective, consider using "machine learning" in this study title, as it may help increase visibility.
ii) This study is very important, and I think it deserves a better introduction. For example, I found the introduction to be way too short and confusing at some points. Consider providing better context about the relationship between brain aging and estradiol-associated events, approaches used by previous studies and their key findings. Yes, the authors cited some works, but superficially. Keep in mind that

this will better support your research problem since I have found the problem to be addressed unclear. Besides, an entire paragraph should be developed to show how machine learning has been used for similar purposes and highlight the innovative approach used in this study. I also found the research problem obscure since it is unclear what specific gaps the study aims to fill. The BrainAGE should be better explained to show why it is an important metric.
iii) Figures and Tables (also in sup. material) require some attention and enhancements. The criteria for including and excluding participants seem arbitrary and are not grounded on scientific findings. Figure 3 should be significantly improved as neither the text nor the Figure specifies each step of the sample workflow in depth. Table 3 also requires some attention, as the variable should be better explained regarding the possibility of bias. Great efforts should be made to improve the description of steps to deploy the machine learning algorithm (GPR), such as hyperparameter decision-making, overfitting techniques, data split between training and validation, etc. Check Boff Medeiros et al. as a good reference for this. Consider also checking Figure 1 as well as benchmarking to be used to describe the method. Single texts in subsections could be separated, e.g., PCA and GPR. The same should be applied to the "removed the linear age" - how did authors perform this? Consider explaining these procedures to support replication and transparency. PS: Sensitivity analysis also requires some improvements in Sup. Table 4.
iv) For the results, consider providing a context about BrainAGE values (e.g., what constitutes "young" and "old") and interpret the practical relevance of changes in BrainAGE or âˆ†BrainAGE. In addition to this, it would be important to better clarify the significance of slope values in tables, especially for those readers without statistical backgrounds. Additionally, it may be helpful to add sample size information and a quantitative scale for density in figures and tables while explaining the impact of adjustments for confounding variables on the results. In the discussion, the review calls for a deeper quantitative comparison with previous studies (this also has to be present in the introduction), distinguishing those directly related to estradiol and BrainAGE from those focused on broader cognitive outcomes. I recommend linking discussions to the presented results (finding X with Table Z or Figure Y). The conclusions should be split into three subsections showing contributions for practice, theory limitations and future works. In fact, limitations regarding ethnic diversity (e.g., predominance of British participants) should be highlighted and discussed, as well as how it may impact bias and choices for future works.
Abstract
i) The context clearly shows that the lack of estradiol may have played a significant role in causing/affecting mental illnesses like dementia. Still, I think that authors jump directly to the objective without highlighting the research problem or literature gap. For example, is this the first study investigating the link between brain aging and estradiol-associated events? Did previous studies try similar approaches using machine learning as a central technique? If so, what are the limitations that this study could support in advancing the field of research to fill out a literature gap? how does it differ from or build on previous studies? Consider providing more information for readers about it. In addition to this, I could not find anything related to brain aging since this will be the variable (central for this study) to be investigated with estradiol. Yes, the context mentions dementia and other related illnesses, but brain aging is very specific. Thus, consider making the context more focused on brain aging.
ii) Besides, a "well-validated machine learning approach" is vague. Is this related to a specific machine

learning technique, e.g., XGboost, SVM, etc? Or has a framework previously been validated in other similar contexts? Consider providing more information about the methodological procedures related to machine learning to enhance the method's credibility.
iii) What kind of medical exams were used to assess brain aging? e.g., MRI and/or other exams? Besides, in the sentence "We observed less brain aging in women", how much less (%)? In addition, the notion of cross-section and longitudinal study should be described before the results, as this was your research strategy.
iv) Consider excluding this sentence, "effects were small and no direct measures of estradiol were obtained in the current study," or explain better because it does not sound good, which almost invalidates this research as it sounds abrupt and may give the impression that the study is insignificant. Instead, highlight how these small observed effects and the absence of direct measurements of estradiol contribute to the literature, e.g., fill research gaps and open avenues for future research such as...???
v) The title could be improved to "using machine learning to explore the impact of estradiol on brain aging: insights from menarche to menopause". Keep in mind that the use of "machine learning" will definitely increase visibility.
Introduction
i) Consider expanding results of postmenopause studies (e.g., 3-6) that show dementia in women is known to increase around menopause. For example, cite the authors et al., their approaches and key findings. The way it is presented gives too much effort for readers to look into these papers and check the main results.
ii) Before presenting the research problem, an entire paragraph should show more context about these studies above and 4,7-14,18-20. In this regard, expand the problems related to dementia and what are the conclusions of previous research so far. In addition, it provides the main approaches used to understand the link between estradiol and dementia in women.
iii) Regarding the research problem, it is unclear what specific gaps the study aims to fill. For example, the authors do not explicitly state whether previous studies have failed to use longitudinal approaches or whether there is controversy about the relationship between estradiol and brain health. I wonder, if there is no controversy, what is the need for this study?
iv) The BrainAGE algorithm should be better explained to show readers what it means or why it is an important metric, especially for those unfamiliar with the concept, as this can lead to confusion.
v) Although I have no experience in this area, I still found the argumentative structure somewhat non-linear. For example, the introduction switches between different topics (risk of dementia, menarche, menopause, estradiol, brain age) without building a fluid narrative that connects all the elements cohesively.
vi) The authors mention the machine learning technique to estimate brain age, which, in my opinion, is an important innovative factor of this research. However, it is mentioned superficially. A full paragraph should be developed explaining why this approach is relevant and what related work has been developed using similar techniques. This is crucial to strengthen the justification for this study. For example, DE LANGE, Ann-Marie et al. Cumulative estrogen exposure, APOE genotype, and women's brain aging-a population-based neuroimaging study. bioRxiv, p. 826123, 2019.
Method
i) Supplemental Table 3 presents the ethnic background of women with longitudinal MRI data used in

this study. Consider including percentages to show the proportion of each group in relation to the total. I wonder if the authors should report factors that could impact the study, such as age, socioeconomic status, or previous health conditions, which could vary between ethnic groups and affect the results. Also, "British" seems very generic. Could different parts of the UK show nuances that should be considered?

ii) I got curious about the inclusion and exclusion of participants - is there a reference to justify age at menarche was younger than ten or older than 18, or whose age at menopause was younger than 45 or older than 60? If so, consider providing a source or a brief explanation to support replication. Keep in mind that these ranges may seem arbitrary without a clear justification.

iii) Figure 3 shows the workflow of sample collection. Consider providing the criteria for inclusion and exclusion used, such as previous diagnosis, medication use, or other conditions during each stage. Besides, separate into stages 1, 2, etc. In addition, instead of just presenting absolute numbers (n), excluding percentages between each stage would give a clearer idea of the proportion of participants removed at each stage, e.g., "36% of women with longitudinal data were excluded for missing X and Y reasons.". This information could be presented on the right side of each step, and new arrows between existing ones ->.

iv) Table 3 presents the descriptive characteristics of the sample of women included in the study, detailing variables related to age, among others. As a recommendation for improvement, consider including the percentage of women who met each inclusion criterion, e.g., how many live births, hysterectomies or oophorectomies were distributed among the final participants (n=1,006)? This could help to check for important biases. In addition, the authors could explain better why these variables are considered for analysis for a broader audience. For example, I am interested in the results of this study because of the use of machine learning techniques. Still, I lack expertise in brain aging and estradiol, so it is hard to understand some issues. For example, In Table 3, the variable related to the number of women with hormone replacement therapy (yes: 306 | no: 700) makes me wonder if this difference could impact the results somehow, i.e., HRT could affect brain aging and introduce variation in outcomes. If not, make this explicit after calling Table 3 in the text, showing the importance of each variable for the study. The same should be applied to other variables like the number of women with hysterectomies (yes: 48 | no: 958), etc.

v) The authors mention the application of PCA for data reduction but lack detail on how the principal components were selected. For example, there is no mention or description of how the data were normalized before PCA, which is essential for the components to be comparable.

vi) Regarding the machine learning (Gaussian Process Regression) applied in this study, please explain the reason for specifically selecting this model and not others. Furthermore, were the hyperparameters (e.g., 100 for the constant mean function and -1 for the likelihood function) set? Also, should the procedures regarding the 10-fold approach be expanded, e.g., how the data was split between training and validation? Were any techniques applied to avoid overfitting, such as cross-validation, etc? Besides, is there any chance of sharing the dataset on GitHub to assess and allow replication? How was data split into data training and validation? Check the methodological procedures of the following paper as a reference for good practice regarding these details: BOFF MEDEIROS, NatÃ¡lia et al. Predicting the length-of-stay of pediatric patients using machine learning algorithms. International Journal of Production Research, p. 1-14, 2023.

vii) Supplemental Table 4 addresses the adjustment for a factor called "GLS", which was used to adjust the results in the sensitivity analyses. This table could be improved in some ways. For example, the authors did not justify why the 16 specific variables were chosen to represent the participants' lifestyles - selection criteria based on literature or statistical relevance? Furthermore, I wonder how the data were adjusted, given that the variables have different units of measurement. Also, consider showing how each variable weighs on the participants' lifestyles, e.g., physical activity may be more impactful than watching TV.

Results

i) In Figure 1, the authors could improve the interpretation of the magnitude of the Y-axis scale (BrainAGE index) by indicating the ranges of practical relevance. For example, I wonder how much represents a "young" value (-10 or -5)? Likewise, how much represents an "old" value (+5 or +10)? It would also be important to add information about the sample size in each ratio (number of points used in each panel - A, B and C). Perhaps show examples of how these values translate to brain health or aging, e.g., does -10 years mean X% reduction in some risk?

ii) Furthermore, there is no quantitative scale to show the meaning of the colors (e.g., points per area on graphs), although the legend indicates that the colors represent density. Consider including some of this information (e.g., n. of samples) in Table 1.

iii) Not much to add to Figure 2 and Table 2, but the same improvements that I have provided in Figure 1 and Table 1. Still, as I mentioned in Figure 1, the authors can explain what a change of -5 or +5 years in the âˆ† BrainAGE Index represents. I wonder if there is some cutoff or threshold (e.g., +10 or -10 years) that has clinical or scientific relevance... Without this practical interpretation, the values on the Y-axis are just abstract numbers. Remember that a reader may be uncertain about the magnitude of the effects, e.g., is -5 years in BrainAGE meaningful for brain health? Are values like +1 year or -1 year considered important, or are they normal margins of error of the model (similar to my question about the BrainAGE index)?

iv) In Supplemental Table 1 and Sup. In Table 2, the authors could briefly explain the slope values in practical terms. For example, what does a change of -0.11 or -0.06 in BrainAGE mean for each additional year in the reproductive period? Furthermore, for analyses that were adjusted for multiple variables (e.g., BMI, blood pressure), the authors could make clear how these adjustments impacted the results - did the coefficients change significantly after adjustment?

Discussion

i) The authors mention related work such as 8,10,13,14, etc, showing similar findings. However, there is no quantitative data demonstrating alignment with studies, e.g., how similar are the results of this study to previous findings? e.g., correlations, effect sizes, etc. Consider providing a new table showing approaches, key findings, and quantitative differences and similarities between this study and the existing literature. The lack of an in-depth comparison makes understanding how this study fills a literature gap/limitations difficult.

ii) Furthermore, these studies should be clear about which ones directly support the association between estradiol and BrainAGE and which of these studies studied dementia or cognition without considering the BrainAGE index and used machine learning as a central technique. In fact, this should also be explicit in the introduction of this study, as I mentioned above.

iii) Authors should put effort into linking the discussions with their results. For example, "both an earlier

menarche and a later menopause were significantly associated with less brain aging, according shown in Figure X, or Table Y"
iv) Supplemental Table 3 highlights a limitation of the study: ethnic representation. The predominance of British women limits the applicability of the results to more diverse populations, which may be relevant in studies of brain health and reproductive factors. Consider further developing these study limitations, showing how future work could be developed in this perspective. Furthermore, the conclusion could be divided into three subsections: a) Contributions for practice (how this study can support clinicians in real-life healthcare settings, e.g., how might these results influence the use of hormone replacement therapy? OR are there any recommendations for monitoring reproductive or brain health?, b) Contributions for theory (how this study fills the literature gaps - this requires more clarification) and c) Limitations and future works.
Note:
I previously reviewed this paper previously in Research Hub and you can read these comments via the Research Hub review page here: https://www.researchhub.com/paper/8496577/a-case-for-estradiol-younger-brains-in-women-with-earlier-menarche-and-later-menopause/grants#threadId=54938.
The original preprint linked to the Research Hub review is here: https://www.medrxiv.org/content/10.1101/2024.04.20.24306043v1.full.

Methods

Are the methods appropriate to the aims of the study, are they well described, and are necessary controls included? Choose an item.

Conclusions

Are the conclusions adequately supported by the data shown? Choose an item.

Reporting Standards

Does the manuscript adhere to the journal's guidelines on minimum standards of reporting? Choose an item.

Choose an item.

Statistics

Are you able to assess all statistics in the manuscript, including the appropriateness of statistical tests used? Choose an item.

Quality of Written English

Please indicate the quality of language in the manuscript: Choose an item.

Declaration of Competing Interests

Please complete a declaration of competing interests, considering the following questions:

- Have you in the past five years received reimbursements, fees, funding, or salary from an organisation that may in any way gain or lose financially from the publication of this manuscript, either now or in the future?
- Do you hold any stocks or shares in an organisation that may in any way gain or lose financially from the publication of this manuscript, either now or in the future?
- Do you hold or are you currently applying for any patents relating to the content of the manuscript?
- Have you received reimbursements, fees, funding, or salary from an organization that holds or has applied for patents relating to the content of the manuscript?
- Do you have any other financial competing interests?
- Do you have any non-financial competing interests in relation to this paper?

If you can answer no to all of the above, write 'I declare that I have no competing interests' below. If your reply is yes to any, please give details below.

I declare that I have no competing interests.

I agree to the open peer review policy of the journal. I understand that my name will be included on my report to the authors and, if the manuscript is accepted for publication, my named report including any attachments I upload will be posted on the website along with the authors' responses. I agree for my report to be made available under an Open Access Creative Commons CC-BY license (http://creativecommons.org/licenses/by/4.0/). I understand that any comments which I do not wish to be included in my named report can be included as confidential comments to the editors, which will not be published.

Choose an item.

To further support our reviewers, we have joined with Publons, where you can gain additional credit to further highlight your hard work (see: https://publons.com/journal/530/gigascience). On publication of this paper, your review will be automatically added to Publons, you can then choose whether or not to claim your Publons credit. I understand this statement.

Yes Choose an item.
